# Single-residue physicochemical characteristics kinetically partition membrane protein self-assembly and aggregation

**DOI:** 10.1074/jbc.RA119.011342

**Published:** 2019-12-16

**Authors:** Ankit Gupta, Radhakrishnan Mahalakshmi

**Affiliations:** Molecular Biophysics Laboratory, Department of Biological Sciences, Indian Institute of Science Education and Research, Bhopal 462066, India

**Keywords:** membrane protein, protein aggregation, protein denaturation, ultraviolet-visible spectroscopy (UV-Vis spectroscopy), spectroscopy, molecular imaging, circular dichroism (CD), imaging, amyloid, neurodegeneration, cross-β aggregates, self-association, thermal perturbation

## Abstract

Ninety-five percent of all transmembrane proteins exist in kinetically trapped aggregation-prone states that have been directly linked to neurodegenerative diseases. Interestingly, the primary sequence almost invariably avoids off-pathway aggregate formation, by folding reliably into its native, thermodynamically stabilized structure. However, with the rising incidence of protein aggregation diseases, it is now important to understand the underlying mechanism(s) of membrane protein aggregation. Micromolecular physicochemical and biochemical alterations in the primary sequence that trigger the formation of macromolecular cross-β aggregates can be measured only through combinatorial spectroscopic experiments. Here, we developed spectroscopic thermal perturbation with 117 experimental variables to assess how subtle protein sequence variations drive the molecular transition of the folded protein to oligomeric aggregates. Using the *Yersinia pestis* outer transmembrane β-barrel Ail as a model, we delineated how a single-residue substitution that alters the membrane-anchoring ability of Ail significantly contributes to the kinetic component of Ail stability. We additionally observed a stabilizing role for interface aliphatics, and that interface aromatics physicochemically contribute to Ail self-assembly and aggregation. Moreover, our method identified the formation of structured oligomeric intermediates during Ail aggregation. We show that the self-aggregation tendency of Ail is offset by the evolution of a thermodynamically compromised primary sequence that balances folding, stability, and oligomerization. Our approach provides critical information on how subtle changes in protein primary sequence trigger cross-β fibril formation, with insights that have direct implications for deducing the molecular progression of neurodegeneration and amyloidogenesis in humans.

## Introduction

Membrane proteins intrinsically display a high aggregation load. With only 5.6% of membrane proteins free of aggregation, these indispensable biomolecules also cause numerous neurodegenerative diseases (1–4). Numerous excellent studies have addressed the global mechanism of amyloid formation (reviewed in Refs. 5 and 6); the molecular morphology and architecture of amyloid fibrils obtained from ∼50 peptides and proteins, as well as their nucleation and growth mechanism have been characterized (6–10). Yet, efforts toward deducing molecular and thermodynamic elements of the protein sequence that drive the switch from a well-defined folded state to disease causing fibrillar aggregates are scarce. Surprisingly little is known in membrane protein aggregation pathways. Specifically, β-barrel outer membrane proteins (OMPs)^2^ are readily prone to aggregation because they exist in kinetically trapped high energy states (6, 11, 12). Simply put, an OMP is readily susceptible to forming β-sheet–rich fibrillar aggregates once its protein-lipid interaction is disrupted. Currently, the molecular elements that control stability of a folded scaffold and nucleate the process of aggregation in membrane proteins are only poorly understood, and demand advanced methodologies and approaches (13). In interesting contrast, and despite our urgent need to understand protein aggregation, we continue to lack atomistic information on the nature of interactions established by amino acid side chains in membrane proteins, which ultimately plays the most prominent role in forming cross-β structures.

The self-assembly of a polypeptide to a folded bioactive protein occurs through controlled, yet rapid formation of thermodynamically favorable native contacts (7–9, 11, 12). Nonnative interactions induce frustrated folding kinetics, causing protein misfolding, aggregation, and often resulting in amyloidogenesis (3, 7–9, 11, 13, 14). The formation of such extended, symmetric aggregates with ordered β-sheet structures has a common cellular and molecular mechanism, and is controlled by the physicochemical property of the protein's primary sequence (4, 14–16). With membrane proteins, aromatic amino acids contribute substantially to both protein stability and aggregation. Indeed, analysis of aggregation-prone polypeptide sequences shows a remarkably high occurrence of aromatic residues, indicating that nonnative contacts are established primarily through Phe, Tyr, or Trp (10, 15, 17). Aromatic residues induce polypeptide self-assembly and stabilize the fibrillar structure by establishing a network of hydrophobic interactions. Additionally, aromatics promote inter-sheet packing of cross-β structures with minimal entropic cost (5, 10, 17). The extent of amyloidogenic aggregates thus formed, and their aggregation kinetics, is controlled by the polypeptide's primary sequence, and minor changes in this sequence drastically alter the protein's overall aggregation propensity (10, 15, 17, 18).

Aromatic residues such as Trp, Phe, or Tyr are well-represented in OMPs (19). Hence, the question remains whether such conserved aromatics would drive aggregation in a folded transmembrane protein. Put simply, would a single residue be able to manifest substantial differences in the overall behavioral phenotype of a β-rich OMP, and dictate the switch between a folded and an aggregated state? Furthermore, which property of this single aromatic residue (π-stacking, hydrophobicity, β-sheet propensity) would be responsible for the aggregation of a folded protein? The increasing occurrence of neurodegenerative diseases demands a better understanding of amyloid formation, for the development of potent therapeutics and inhibitors of protein aggregation to prevent these pathologies. It is therefore of utmost interest to both obtain a universal mechanism of protein aggregation, and develop newer and reliable tools to accurately deduce the kinetic barriers linking membrane protein unfolding with aggregation (6, 13, 20).

Given that the common underlying cross-β fibrillar architecture is shown by a variety of amyloidogenic proteins, it is evident that membrane proteins such as OMPs with a tendency to aggregate in solution can be used as tools to understand the most pressing question of molecular mechanism of protein aggregation. The tremendous molecular complexity and multistep nature of protein aggregation in the environment of the cell makes it nearly impossible to quantitatively measure the energetics of amyloid fibril formation *in vivo*. A quantitative measure of the physicochemical factors regulating the molecular mechanisms of protein self-association can only be obtained using a combination of several spectroscopic methods. Here, we develop spectroscopic thermal perturbation as an effective and rigorous tool to successfully map the molecular process of protein aggregation. OMPs from human origin are structurally large (minimum scaffold is 16-stranded), with a complex topology, and present appreciable oligomerization tendency under native conditions (21). Thus, human OMPs are poor models to develop thermal perturbation strategies to identify how a single amino acid controls protein aggregation. On the other hand, many bacterial OMPs have a small 8-stranded topology, simple architecture, and are monomeric. Nevertheless, they form fibrillar aggregates, and cause pathological neurodegenerative conditions similar to human membrane proteins (22, 23). The study of cross-β fibrillation of bacterial OMPs also has implications in understanding biofilm formation (22, 24). Hence, bacterial OMPs are simple model systems with well-defined structure and topology, to deduce a global mechanism for membrane protein aggregation.

Here, we chose the attachment invasion locus (Ail) protein, an OMP from the category A pathogen *Yersinia pestis*, as our model, to develop molecular tools for the study of membrane protein aggregation. Most interestingly, the simple 8-stranded β-barrel topology of folded Ail also possesses an intrinsic aggregation tendency. Ail is also pharmacologically relevant, as it causes biofilm formation and pathogenesis by autoaggregating in the *Yersinia* outer membrane (25). Ail acts as an excellent model to study protein aggregation because (i) it is a small barrel, (ii) has a simple topology, (iii) is kinetically stabilized, (iv) undergoes oligomerization as a part of its function in the Ail outer membrane, and (v) the two unique evolutionarily conserved tryptophan residues Trp^42^ and Trp^149^ positioned at the solvent interface of Ail serve as ideal sites to address how physicochemical factors of vital interface aromatics dictate the stability *versus* aggregation propensity of an OMP. Using Ail, we developed a combinatorial spectroscopic thermal perturbation toolbox of spectroscopic, scattering, and calorimetry measurements to deduce how the chemical nature of a single residue regulates the aggregation propensity of a membrane protein. We find an unexpected regulation of the kinetic factors that contribute to barrel stability and aggregation, by the physicochemical nature of the residues at the 42^nd^ and 149^th^ positions. We also find that three-dimensional side chain–side chain interactions are critical for OMP stability, providing us with a universal mechanism of protein aggregation and the formation of β-sheet–rich amyloid-like fibrillar aggregates. Our results reveal that Ail aggregation proceeds through the formation of partially unfolded yet structured states that are promoted by Tyr and Phe, but retarded by Trp. Our study sheds light on how the evolution of thermodynamically compromised sequences works in favor of aggregation-prone sequences avoiding cross-β fibrillation *in vivo*.

## Results

### Strategically generated Ail mutants exhibit complete folding

**Figure 1.**
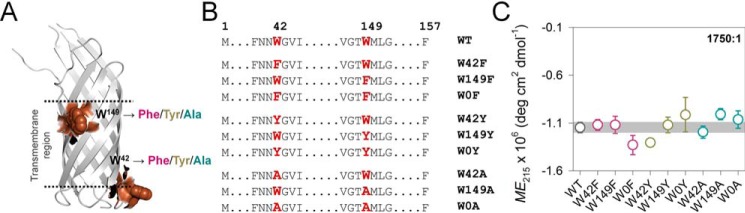
**Ail-WT and its mutants are well-structured.** *A,* cartoon representation of Ail (PDB code 3QRA), highlighting both tryptophans (Trp^42^ and Trp^149^, *brown spheres*) on Ail structure. Here, Trp^149^ is located at the outer leaflet and is highly conserved in a large number of 8-stranded OMPs. Trp^42^ is located at the interface of the inner leaflet and is largely unique to Ail. *B,* list of Trp mutants generated in this study (single mutants and double mutants). The substituted residue (Trp → Phe/Tyr/Ala) is highlighted in *red*. The *number above* the sequence shows the position of Trp in the Ail sequence. *C,* comparison of the secondary structure content using *ME*_215_ of the various Ail mutants folded in LDAO at a DPR of 1750:1. A simplified color pattern is used here: WT (*gray*), Phe mutants (*pink*), Tyr mutants (*yellow*), Ala mutants (*cyan*). Errors are S.D. derived from a minimum of 2–3 independent experiments. The *shaded area* represents the S.D. obtained for Ail-WT. Despite the mutation, all proteins exhibit comparable folding and adopt similar secondary structure content (complete data in Figs. S4–S11).

Aromatic amino acids contribute significantly to both membrane protein stability and protein aggregation. The calculated order of aggregation of the three aromatics follows Tyr > Phe > Trp (17), suggesting that mutational analysis of aromatics can provide information on aggregation characteristics of Ail. The Ail scaffold has 12 tyrosines, 12 phenylalanines, and 2 tryptophans. Although Phe exhibits a near-equal distribution toward the inner leaflet of the outer membrane and the lipopolysaccharide leaflet, Tyr is distributed preferentially toward the lipopolysaccharide leaflet, and both the tryptophans are located at membrane-water interface. Here, both Phe and Tyr show high variability in composition and polarity in their 8 Å vicinity, whereas both indoles are located in chemically similar environments (Fig. 1*A* and Fig. S1). The measurement of aggregation kinetics for a membrane protein requires diverse biophysical studies involving spectroscopic, calorimetry, and scattering measurements, in different lipid-protein ratios and temperatures, which introduces considerable variability in the data. Consistency in the measurements of Ail aggregation can only be obtained when changes are restricted to distinct sites with similar environments and chemically controlled interactions. Therefore, we chose the two Trp residues at positions 42 and 149 (Fig. 1*A*) as our host sites to investigate the molecular mechanism of Ail aggregation.

First, we systematically generated an Ail mutant library where each Trp was substituted with Phe, Tyr, or Ala through site-directed mutagenesis (Fig. 1*B*). Next, we ensured that the starting folded structure of Ail mutants did not influence our measurements by exhaustive screens of lipidic and detergent conditions that supported the correct folding of Ail-WT and all its mutants. Phosphocholine lipid vesicles did not support the complete folding of Ail (Figs. S2 and S3), and could not be used further. Additionally, acidic or neutral pH conditions showed detectable levels of Ail aggregation. Of our screening experiments, the two lipidic detergents that successfully supported the complete folding of Ail were LDAO (*N*,*N*-dimethyldodecylamine *N*-oxide) and DPC (*n*-dodecylphosphocholine) micelles, and in alkaline pH. Ail folds readily in LDAO with no detectable aggregation (Figs. S4 and S5), and retains its folded state (80% monomers, ∼20% oligomers in solution) for >2 days despite incubation at 25 °C. Furthermore, the LDAO headgroup shares similarity in structure and chemical properties with the well-known chemical chaperone trimethylamine *N*-oxide (26). We reasoned that the chaperone-like behavior of the LDAO headgroup will further facilitate our study of OMP aggregation. Therefore, we folded Ail WT and all its Trp mutants in varying detergent-to-protein ratios (DPRs) of 700:1, 1750:1, and 3500:1 of LDAO:28 μm protein. The LDAO concentrations used here are 20-, 50-, and 100-fold above its critical micelle concentration (critical micelle concentration of LDAO in water is ∼1.0 mm). At these LDAO concentrations, Ail exhibits complete folding without the presence of any aggregated species (Figs. S5–S11).

Folded Ail shows several biophysical properties that are distinct from its unfolded counterpart. This allowed us to use independent methods to verify that our starting protein preparations, despite the mutation, were folded reliably and to comparable extents (Fig. 1*C*; complete data in Fig. S4–S11). For example, (i) folded Ail exhibits retarded electrophoretic mobility shift on cold SDS-PAGE gels and (ii) resists cleavage by robust proteases such as proteinase K. (iii) A blue-shifted Trp fluorescence emission spectrum with an emission maximum (λ_em-max_) centered at ∼336 nm is also characteristic of folded Ail. Ail also exhibits (iv) higher fluorescence anisotropy (*r*) and (v) higher lifetime values (〈τ〉) when compared with its unfolded counterpart. (vi) The secondary structure content of folded Ail shows a signature β-sheet structure with high negative ellipticity centered at 215 nm. (vii) We obtain well-dispersed ^1^H and ^15^N resonances in HSQC-TROSY NMR spectra of folded Ail. Overall, folded Ail-WT and all its mutants exhibit comparable spectroscopic parameters (i–vii) in all three DPRs of LDAO (Fig. 1*C* and Figs. S4–S11). These results confirm that within our experimental conditions, Ail attains a well-folded β-barrel conformation in LDAO micelles, the mutation does not affect the final folded state of the protein, and the ensemble of starting conformational states for Ail-WT and its mutants is comparable.

The use of alkaline buffer can affect the ionic state of charged amino acids located in the extra-membranous loop regions of Ail (27, 28). Considering how Ail stability is regulated primarily by residues in the transmembrane domain, the magnitude of protein-LDAO interactions at positions 42 and 149 is greater than intra-protein electrostatic interactions (see Fig. S1), minimizing the influence of charged residues on Ail folding. Additionally, Ail possesses an intrinsic tendency to aggregate. Hence, we subjected all protein preparations to high speed centrifugation to remove aggregates that may form in the folding process. Additionally, scattering measurements (*A*_320_ ≈ 0) and aggregation index calculations (*AI*_340_ ≈ 0) together confirmed that the folded protein samples contain no detectable levels of protein aggregates.

Finally, we minimized artifacts in the spectroscopic measurements that may arise from marginal heterogeneity in sample preparations by (i) verifying that oligomeric species do not show substantial sample-to-sample variation using electrophoretic mobility shift assays, (ii) using only preparations with *A*_320_ and *AI*_340_ ≈ 0 in all our experiments, (iii) considering only data that shows a substantially significant difference in the spectroscopic parameter, and (iv) verifying the measurements across 117 different parameters (described in the next section). In particular, we ensured that our inference is reliable, by drawing conclusions from a statistically significant number of independent parameters and independent experiments.

### Global analysis of 117 biophysical variables provides reliable assessment of protein stability and aggregation

Typically, transmembrane β-barrel proteins are kinetically trapped structures in the membrane, with a high energy barrier separating the transition from folded to the unfolded state (1, 2, 29). For example, unfolding of Ail, which is folded in micelles, requires temperatures >55 °C (Fig. S12); interestingly, this process is irreversible. Put simply, Ail aggregates when it is unfolded by heating. Other β-barrel OMPs such as OmpA, OmpLA, OmpT, OmpW, OmpX, and human mitochondrial porins (12, 29) show similar behavior, which arises from the unusually high kinetic stability of these protein scaffolds. Therefore, kinetically stabilized OMPs are aggregation-prone.

Ail exhibits a cooperative loss in secondary structure content and denatures irreversibly upon heating to form temperature-induced aggregates (Fig. S12). These aggregates display cross-β fibrillar morphology similar to those typically seen in amyloidogenic aggregates from debilitating neurodegenerative diseases (3, 5). We used the aggregation property, achieved upon perturbation of Ail structure by temperature, as a direct measure to both identify and quantify physicochemical features of the protein primary sequence that cause membrane protein aggregation. Here, we applied three different spectroscopic methods (far-UV circular dichroism (CD), light scattering using UV spectrometry (scattering), and differential scanning microcalorimetry) to monitor 16 independent parameters (Table 1) that can together represent changes in protein structure, protein aggregation, and enthalpic changes upon protein unfolding and aggregation. Notably, the slow aggregation event of Ail, which occurs in days, is accelerated by the use of temperature. In addition, the use of temperature-induced denaturation allows the use of multiple ramp rates for each process, providing information on the kinetics of the aggregation event. Furthermore, we carried out all our experiments in the three DPRs used to fold Ail, namely 700:1, 1750:1, and 3500:1 (corresponding to ∼9, ∼23, and ∼45 micelles, respectively, per protein), which provides information on the role of lipid content on protein aggregation. Put together, we obtained a total of 117 experimental variables that now provide a reliable molecular measure of Ail stability, unfolding, and aggregation.

**Table 1:** **Major biophysical parameters measured in spectroscopic thermal perturbation**

Parameter measured*^a^*		Process or characteristic monitored	Protein characteristic deduced
*ME*	*ME*_215_	Secondary structure (β-sheet) content	Extent of protein folding
*T_m_*_-start_	*ME*_215_	Temperature at which protein unfolding is initiated	Protein stability
	*A*_340_	Temperature at which protein aggregation is initiated	Protein stability
*T_m_*	*ME*_215_	Midpoint of protein unfolding and loss in β-sheet structure	Protein stability
	*A*_340_	Midpoint of protein oligomerization or aggregation	Stability of folded monomer and aggregation tendency of folded protein
*T_m_*_-end_	*A*_340_	End point temperature of protein aggregation	Protein stability and aggregation propensity
*E*_act_	*ME*_215_	Activation energy of unfolding and aggregation	Protein stability
Δ*T_m_*	*A*_340_	Difference between *T_m_*_-end_ and *T_m_*_-start_; monitors cooperativity of protein aggregation	Indirect measure of the rate of aggregation
Δ*A*_340_	*A*_340_	Difference in *A*_340_ before and after protein oligomerization or aggregation	Aggregation propensity
*T_m_*_-UF_	*C*_p_	Midpoint temperature of the global unfolding of the barrel	Protein stability
*T_m_*_-Agg_	*C*_p_	Midpoint temperature of global aggregation of the barrel	Aggregation propensity
*AI*	*A*_320_	Aggregation index measured at 320 nm	Aggregation propensity
	*A*_340_	Aggregation index measured at 340 nm	Aggregation propensity
ThT	λ_480_	Amount of β-sheet–rich fibrillar aggregates	Aggregation propensity
*ME*_215-B/A_	*ME*_215_	Difference in *ME*_215_ before and after thermal denaturation	Extent of protein aggregation
*P*_sol_	*A*_340_	Concentration of folded protein in solution after thermal denaturation	Extent of protein aggregation

*^a^ ME,* molar ellipticity measured using CD spectropolarimetry; *A*_340_ or *A*_320_, absorbance measured using UV absorbance spectroscopy at 340 or 320 nm; *C*_p_, molar heat capacity measured using DSC; ThT, thioflavin T fluorescence measured at 480 nm.

It is evident that our spectroscopic studies provide a substantial number of independent measurements that can be studied analytically, and transformed to biophysical constraints to denote protein aggregation. A sequential individualized analysis of each fitted parameter provides the fractional contribution of each measurement to Ail aggregation. However, such an analysis is also complicated by contributions from subtle changes that each methodology monitors, or minor variations in the folded protein state, sample preparation, or the redistribution of local interaction networks upon point mutations. A solution to this is a generalized compartmental analysis of the global unfolding and aggregation process that utilizes shared fitting parameters and distributed variables. Such a global data analysis will together provide molecular information on each step of the unfolding and aggregation processes. Additionally, this analysis technique accounts for fractional contributions only when they are significant, whereas also overcoming artifacts and errors associated with the measurement of each biophysical parameter. Therefore, we applied a global comparison of our 16 thermal parameters (Table 1) and 117 experimental variables (global analysis methodology is detailed under “Experimental procedures”), to obtain the molecular elements that link aromatics with protein aggregation. In the subsequent sections, we describe our findings from this global analysis.

### Ail scaffold stability is regulated independently by the chemical nature of interfacial aromatics

**Figure 2.**
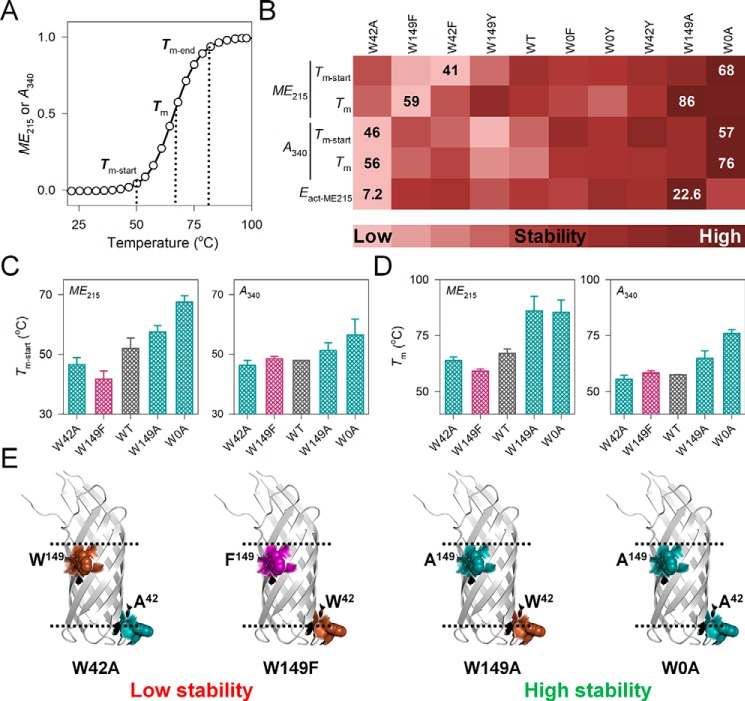
**Spectroscopic measurements reveal Ail stability is controlled by the physicochemical nature of interface residues.** *A,* schematic representation of thermal parameters measured using *ME*_215_ or *A*_340_. The start-point (*T_m_*_-start_), midpoint (*T_m_*), and end point (*T_m_*_-end_) of unfolding and aggregation are mapped on the profile. *B,* global comparison of thermal parameters (*T_m_*_-start-ME_, *T_m_*_-start-A340_, *T_m_*_-ME_, *T_m_*_-A340_, and *E*_act-ME215_) at the DPR of 1750:1 and temperature ramp rate of 1 °C/min. Proteins are arranged from low stability (*light shade*; left extreme) to high stability (*dark shade*; right extreme), as deduced from global analysis. *Numbers* within the heat map indicate the lowest and highest value of each parameter (complete data in Figs. S16, S17, and S20). Comparison of (*C*) *T_m_*_-start_ and (*D*) *T_m_* measured using *ME*_215_ (*left panel*) and *A*_340_ (*right panel*), for the most and least stable Ail mutants deduced from the global analysis shown in Fig. 2*B*. Data for Ail-WT are included for comparison. A simplified color pattern is used here: WT (*gray*), Phe mutants (*pink*), Ala mutants (*cyan*). *Error bars* represent S.D. derived from a minimum of three independent experiments. *E,* residue pairs that confer the highest and lowest stability to Ail structure are depicted on the cartoon representation of the protein. Trp^42^ and Trp^149^ are colored as: Trp, *brown*; Phe, pink; Ala, *cyan*. Ail-W42Y, W149A, and W0A show the highest stability.

Protein aggregation is a complex phenomenon, and detailed understanding requires a systematic experimental approach that measures several interdependent and independent variables. As seen with most aggregation-prone proteins, the unfolding and aggregation processes in Ail are coupled, and manifest as a two-state transition. For simplicity, we first analyzed all the thermal parameters at the DPR of 1750:1 and a temperature ramp rate of 1 °C/min. The thermal parameters corresponding to protein stability are listed in Table 1 (see Fig. 2 for the results of global analysis; also see Figs. S13–S15). Here, we first deduced if the mutation of interface indoles Trp^42^ and Trp^149^ can affect the Ail scaffold. Ail has two interface indoles at positions 42 and 149 (see Fig. 1*A*). Global comparison of the major differences in thermal parameters reveals that Trp substitution affects both Ail stability and aggregation propensity. WT Ail shows moderate thermal stability, as it is positioned at the middle of the heat map (Fig. 2*B*). Site-selective Trp → Ala substitution (W149A and W0A (W42A/W149A double mutant)) increases barrel stability (right extreme of the heat map in Fig. 2*B*, and Figs. S16 and S17), whereas Ala is less favored at position 42 (left extreme of the heat map in Fig. 2*B* and Figs. S16 and S17). At the other extreme are the Phe mutants, which exhibit lowered stability at both positions 42 and 149. The Tyr mutants exhibit moderate stability, as seen for Ail-WT. Thermal measurements show that both tryptophans can be substituted to aliphatic or aromatic residues despite the reduction in hydrophobicity at the interface (Trp^42/149^ → Tyr or Ala) (Fig. 2, *B–E*, and Figs. S16 and S17). Considering how Trp is a metabolically expensive residue, our observation raises the evolutionary need for Trp, or other aromatics in general, in β-barrel sequences. In addition, conserved Trp → Phe/Tyr substitutions differentially influence Ail stability.

To further confirm that the observed effect is not exclusively due to Trp removal, we compared the stability of all W42*X* with W149*X* mutants (here *X* = Phe/Tyr/Ala), using all the thermal parameters. Indeed, we find a substantial difference in the thermal stability of the W42*X* and W149*X* mutants (Fig. 2*B*), with a position-specific effect at both 42^nd^ and 149^th^ residues. For example, Ala is more stabilizing at the 149^th^ position (W42A is at the left extreme and W149A is at the right extreme of the heat map in Fig. 2*B*); furthermore, the effect is nonadditive with stability of W149A > W0A > W42A. In contrast, Trp → Phe substitution is deleterious for the barrel, as both Ail W42F and W149F show lowered stability (W42F and W149F are on the left extreme of the heat map in Fig. 2*B*). Interestingly, in contrast to Ala and Phe, the Trp → Tyr substitution is tolerated (moderately stabilizing) at both 42^nd^ and 149^th^ positions (the Tyr mutants are in the middle of the heat map in Fig. 2*B*). Furthermore, the nonadditive effect of mutations on Ail stability (varying stability of Ail Trp^42,149^ → Phe/Tyr/Ala as compared with W42*X* and W149*X*) confirms that Trp, Phe, Tyr, and Ala show position-specific barrel stabilization., Minor differences in the local environment of both residues (see Fig. S1) could alter local protein–detergent interaction dynamics, and also contribute to our observations. Our results confirm that the variation observed in Ail stability is the cumulative result of Trp substitution and the substituted residue, and that the chemical nature of the amino acid at the 42^nd^ and 149^th^ positions plays an important role.

### Stability of Ail is linked to activation energy barrier of unfolding and aggregation

Next, to deduce how interface aromatics regulate the stability and aggregation of Ail, we monitored the activation energy barrier (*E*_act_) separating the native and aggregated protein states. Most OMPs are kinetically trapped in high-energy states (1, 2, 29). This allows the β-barrel to resist unfolding under adverse cellular conditions. Therefore, detailed dissection of the aggregation kinetics requires that kinetics specific to the metastable assemblies be demarcated from the activation energy barriers in the free energy landscape separating the relevant states (5, 7–9, 11, 30). In Ail, the unfolding and aggregation processes are coupled, and the protein exhibits Arrhenius behavior with two-state aggregation kinetics. A distinct lag phase is absent in the kinetic traces (Fig. S18), and we observe that the rate of unfolding (*k*_u_) increases linearly with temperature (Fig. S19). Transient intermediates in the aggregation pathway appear as non-Arrhenius behavior profiles at high temperatures only for some mutants and only in low DPRs (Figs. S13 and S19).

The global analysis of *E*_act_ suggests that similar to stability, the *E*_act_ is also affected by Trp substitution. Interestingly, proteins with high stability also show high *E*_act_ (see right extreme in the heat map in Fig. S20). Similarly, proteins with low stability show low *E*_act_ (Fig. S20). Hence, the thermal stability of Ail depends on the activation energy barrier of its unfolding and aggregation. In addition, Trp, Tyr, Phe, and Ala show position-specific stabilization of the barrel and a nonadditive effect on the *E*_act_ (see Fig. S20 for differences in *E*_act_ of Trp^42,149^ → Phe/Tyr/Ala when compared with W42*X* and W149*X*). The latter finding validates our deduction that the stability of Ail depends on both tryptophan substitution and the chemical nature of the substituted residue.

Next, to address the role of stability and *E*_act_ in Ail aggregation, we asked: (i) what is the final state of thermally-denatured Ail, and (ii) what is the correlation between Ail stability, activation energy, and aggregation propensity. To address these questions, we monitored the effect of temperature ramping rate on the parameters that define Ail stability (*T_m_*_-start-ME_, *T_m_*_-ME_) and *E*_act_, *versus* Ail aggregation (*A*_340_ measurements: *T_m_*_-start-A340_, *T_m_*_-A340_) (Table 1). We find that the loss in secondary structure is independent of ramping rate but the barrel oligomerization and aggregation of Ail show a linear dependence on the increase in ramp rate (Fig. S21). Hence, whereas the initial folded state of WT-Ail and the mutants is nearly identical (Figs. S4, S6–S8, S10, and S11), changes in barrel oligomerization rates are a consequence of the mutation affecting the transition state to protein unfolding. In turn, this affects the final aggregated state of the protein. Hence, the stability and aggregation of Ail are under kinetic control. Additionally, this aggregation process is regulated by the chemical nature of the amino acid (Trp/Phe/Tyr/Ala) present at both the sites, and in a position-specific manner. We also surmise based on these results that the moderate stability of WT-Ail is a likely consequence of an optimal balance of stability and aggregation, which confers structural plasticity to the barrel for effective function.

### Kinetic partitioning of Ail scaffold stability and aggregation

Next, to obtain the global information on Ail unfolding and aggregation, we combined the data from stability, scattering, and aggregation measurements. For simplicity, we again used the DPR of 1750:1 and temperature ramp rate of 1 °C/min for our initial analysis. The thermal parameters corresponding to aggregation kinetics and aggregation propensity are listed in Table 1. Briefly, we monitored the (i) end point (*T_m_*_-end-A340_) and cooperativity of aggregation (Δ*T_m_*_-A340_), (ii) increase in *A*_340_ due to aggregation (Δ*A*_340_), (iii) aggregation index (*AI*_340_), and (iv) the midpoint temperature of aggregation (*T_m_*_-Agg_). In all measurements, the reference-corrected thermal denaturation profile was used to derive the specific thermal parameter. All parameters were then normalized between 0 and 1, compared globally first at the DPR of 1750:1 and then across all DPRs, as described under “Experimental procedures.”

**Figure 3.**
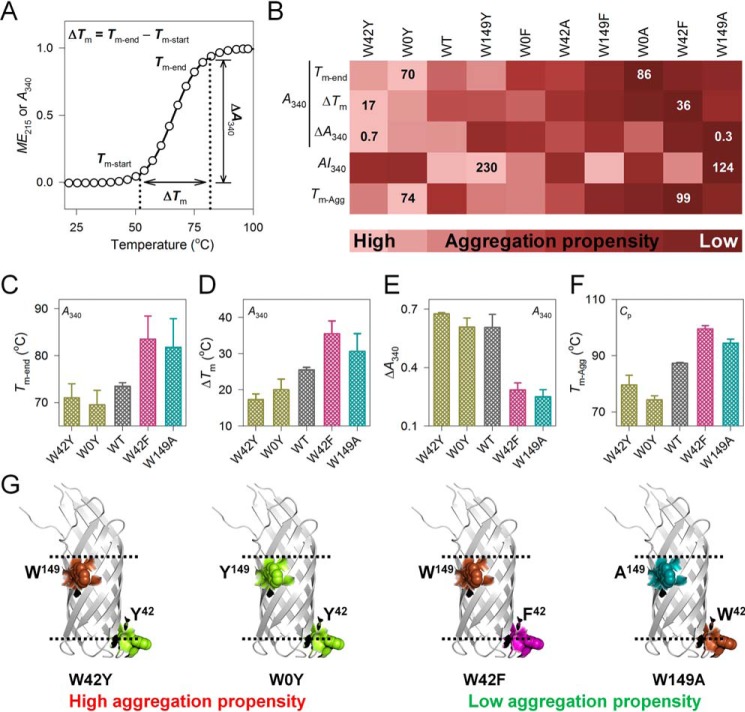
**Chemical nature of interface tyrosines increases aggregation propensity of Ail by kinetic partitioning.** *A,* schematic representing thermal parameters measured using *ME*_215_ or *A*_340_. The profile illustrates how *T_m_*_-end_, Δ*T_m_*, and Δ*A*_340_ represent. *B,* global comparison of thermal parameters (*T_m_*_-end_, Δ*T_m_*, Δ*A*_340_, *AI*_340_, and *T_m_*_-Agg_) at the DPR of 1750:1 and temperature ramp rate of 1 °C/min. The global comparison measures the aggregation propensity of folded Ail and its mutants. Proteins are arranged from high (*light shade*; left extreme) to low (*dark shade*; right extreme) aggregation propensity, as deduced from global analysis. *Numbers* within the heat map indicate the lowest and highest value of each parameter (complete data in Figs. S22–S24). Comparison of (*C*) *T_m_*_-end_, (*D*) Δ*_T_*_m_, (*E*) Δ*A*_340_, and (*F*) *T_m_*_-Agg_ for the mutants with the highest and lowest aggregation propensity, deduced from the global analysis shown in Fig. 3*B*. A simplified color pattern is used here: WT (*gray*), Phe mutants (*pink*), Tyr mutants (*yellow*), Ala mutants (*cyan*). *Error bars* represent S.D. derived from at least 3 independent experiments. *G,* residue pairs that confer the highest and lowest aggregation propensity on folded Ail barrel are shown schematically (color pattern used is: Trp, *brown*; Phe, *pink*; Tyr, *yellow*; and Ala, *cyan*). W0A, W42F, and W149A exhibit lowest aggregation propensity, whereas the Trp → Tyr mutants are highly susceptible to aggregation.

Our measurements reveal that Trp substitution substantially affects Ail aggregation (Fig. 3). Interestingly, the correlation of stability with aggregation propensity is nonlinear and we observe that those proteins with high or low stability do not correspondingly exhibit low or high aggregation propensity, respectively. Although the Phe mutants with low stability show moderate aggregation propensity, the highest aggregation propensity is seen for the Tyr mutants (note how the Tyr mutants are to the left extreme of the heat map in Fig. 3; complete data in Figs. S22–S24). In other words, the highest aggregation propensity is shown by the moderately stabilized Trp → Tyr mutants (W42Y and W0Y). The difference in aggregation propensity is nonadditive for the W42X and W149X mutants (note the differences in aggregation propensity of Trp^42,149^ → Phe/Tyr/Ala when compared with W42*X* and W149*X* in Fig. S24). Furthermore, the aggregation propensity of the mutants does not correlate well with *E*_act_ (compare complete data shown in Fig. S20 with Fig. S24), and suggests that in Ail, barrel stability and aggregation are controlled independently (Fig. 3*G*).

**Figure 4.**
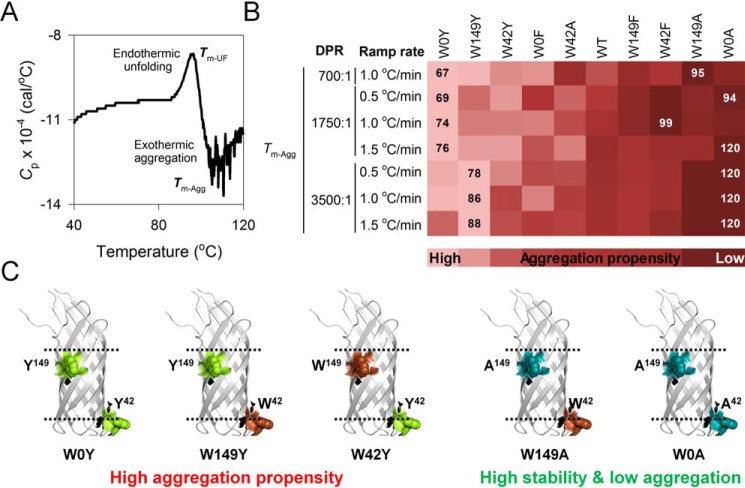
**Intrinsic aggregation tendency of Ail is augmented upon Trp → Tyr substitution.** *A,* representative differential scanning microcalorimetry profile of Ail-WT, showing the change in specific heat capacity (*C*_p_) upon thermal denaturation. The endothermic (protein unfolding) and exothermic (protein aggregation) transitions are measures of *T_m_*_-UF_ and *T_m_*_-Agg_, respectively. *B,* global comparison of *T_m_*_-Agg_ measured for Ail mutants in various DPRs and ramp rates. Mutants are arranged from high (*light shade*; left extreme) to low (*dark shade*; right extreme) aggregation propensity, deduced from the global analysis (complete data in Fig. S27). *Numbers* within the heat map represent the lowest and highest value of the measured parameter. *C,* mutants with high (or low) propensity to aggregate and residue pairs that stabilize Ail are mapped on its structure. Color pattern used is: Trp, *brown*; Phe, *pink*; Tyr, *yellow*; Ala, *cyan*. Trp → Tyr substitution augments the aggregation propensity of Ail scaffold.

To confirm our observations, we used heat capacity curves from differential scanning microcalorimetry measurements. The heat capacity profile of Ail shows contributions from the unfolding as well as aggregation processes (Fig. 4*A*, Figs. S25 and S26), and provides us with heat capacity values (*C*_p_) of the endothermic and exothermic transitions, respectively. From here, we derived the aggregation temperature (*T_m_*_-Agg_), and we compared the results using global analysis in various DPRs and ramp rates (Fig. 4; complete data in Figs. S25–S27). The results validate our observations from aggregation kinetics measurements, where the Trp → Tyr substitution (W0Y > W149Y > W42Y) shows the highest aggregation propensity (note the clustering of the Tyr mutants on the left of the heat map in Fig. 4*B*; complete with data in Fig. S27).

Overall, we observe that the stability and activation energy measurements of Ail correlate poorly with the aggregation kinetics and aggregation propensity (see Table 2). This may arise either from the differences in the molecular elements that promote Ail oligomerization and aggregation, or due to the variation in the final state of thermally denatured Ail. To examine this further, we compared the effect of temperature ramping (Fig. S28) and the rate of aggregation (Fig. S29). Four parameters primarily carry this information, which are *T_m_*_-end_, *T_m_*_-Agg_, Δ*T_m_*_-A340_, and Δ*A*_340_ (see Table 1). Interestingly, Ail aggregation propensity correlates inversely with increase in the temperature ramping rate (Fig. S28). Additionally, we observe a position-specific contribution of Trp, Tyr, Phe, and Ala on the rate of Ail aggregation (Fig. S29), with proteins with the highest aggregation propensity (Trp → Tyr mutants) (Fig. 4) also showing the highest rate of aggregation (Fig. S29). Because Ail is a kinetically stabilized barrel, this kinetic partitioning regulates barrel stability and aggregation propensity through a mechanism similar to how amyloids are formed (30, 31). We conclude that the cooperativity and the rate of aggregation dictate the fate of the barrel, with our results confirming the importance of the kinetic component on the stabilization of folded Ail.

**Table 2:** **Correlation between thermal stability and aggregation of various Trp mutants**

Mutation	Stability	Activation energy	Aggregation propensity	Rate of aggregation
Trp → Phe	Low	Moderate	Low–moderate	Low–moderate
Trp → Tyr	Moderate	Moderate–high	High	High
Trp → Ala	High	Moderate–high	Low	Low–moderate

### Ail aggregates form self-associated structured oligomers

**Figure 5.**
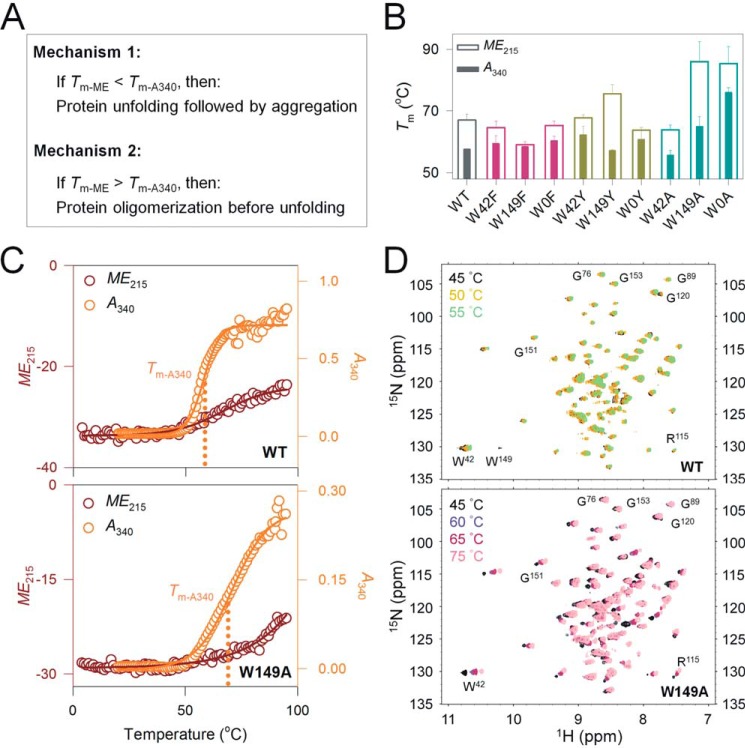
**Ail aggregation occurs through structured oligomers.** *A,* two possible mechanisms of protein aggregation interpreted from *T_m_*_-ME215_ and *T_m_*_-A340_. *Mechanism 1* requires complete protein unfolding, which is followed by aggregation. *Mechanism 2* supports the formation of structured aggregates from partially unfolded proteins. *B,* histogram comparing *T_m_*_-ME215_ and *T_m_*_-A340_, to deduce the mechanism of Ail aggregation. *Error bars* represent S.D. derived from at least 3 independent experiments. The data supports *Mechanism 2*, with a *T_m_*_-ME215_ > *T_m_*_-A340_ indicating that protein oligomerization and aggregation occurs before complete unfolding or loss in secondary structure (complete data in Figs. S30 and S31). *C,* comparison of thermal denaturation profiles of Ail measured using far-UV CD (*ME*_215_) (*maroon*) and UV-visible spectroscopy (*A*_340_) (*orange*). Representative profiles for WT (*top panel*) and W149A (*bottom panel*) at a temperature ramp rate of 1 °C/min and a DPR of 1750:1 are shown (see Fig. S31 for complete data of all mutants). Ail shows cooperative aggregation but slow unfolding. Here, at the temperature nearing the aggregation midpoint (*T_m_*_-A340_, *dotted line*), Ail does not show significant loss in its secondary structure (*ME*_215_), and the barrel remains folded. Put simply, at *T_m_*_-A340_, the unfolded fraction of Ail is ≈0. *D,* representative ^1^H-^15^N HSQC-TROSY spectra of folded Ail-WT (*left*) and W149A (*right*) (DPR of 1750:1), at various temperatures. Well-dispersed resonances observed for both proteins even at temperatures near to their midpoint of thermal denaturation (∼65 °C for Ail-WT and ∼75 °C for W149A), suggests that the Ail remains structured even at midpoint temperatures for aggregation (complete data in Fig. S32). Therefore, Ail aggregation occurs through the association of structured intermediates.

Protein unfolding largely precedes aggregation (18) (Fig. 5*A, Mechanism 1*), but proteins that self-associate in their partially folded states and lead to aggregation have also been documented (29) (Fig. 5*A, Mechanism 2*). To understand which process is predominant in Ail, we compared our measurements from stability and aggregation. Here, *ME*_215_ measurements (*T_m_*_-ME_) provide information on coupled unfolding and aggregation, whereas *A*_340_ measurements (*T_m_*_-A340_) provide information on only the aggregation process (Fig. 5*A*). We find that all the proteins show *T_m_*_-A340_ < *T_m_*_-ME_, where the *T_m_*_-ME_ is 1–20 °C higher than *T_m_*_-A340_ for the different mutants (Fig. 5, *B* and *C*; complete data in Figs. S30 and S31), suggesting that Ail oligomerization occurs before the complete loss in protein secondary structure. In other words, at *T_m_*_-A340_, an unfolded fraction of Ail ≈ 0 (as judged from *ME*_215_; Fig. 5*C*). Hence, Ail aggregation occurs by the association of partially unfolded proteins (Fig. 5*A, Mechanism 2*).

To confirm that Ail retains secondary structure at temperatures nearing the thermal denaturation midpoint (*T_m_*), we measured backbone structural changes with ^1^H-^15^N HSQC-TROSY. If Ail is largely unfolded at these temperatures (expected for Mechanism 1), the NMR data would be typically characterized by resonances that are poorly dispersed and centered ∼8.0 ± 0.15 ppm in the ^1^H and 115–125 ppm for non-Gly backbone resonances in the ^15^N dimensions (32). Furthermore, the spectra would present poor signal-to-noise and poor resolution, if Ail is misfolded or aggregated. However, we obtained well-dispersed resonances at all temperatures between 40 and 75 °C for Ail (Fig. 5*D*, complete data in Fig. S32), indicating that the secondary structure content of Ail is preserved even at temperatures where aggregation is observed, supporting Mechanism 2 (Fig. 5*A*).

We note here that Mechanism 1 would require unfolding to precede aggregation, and would yield *T_m_*_-ME_ values that are considerably lower than *T_m_*_-A340_. Furthermore, the well-dispersed resonances in the NMR spectra supports our conclusion that Ail aggregation follows Mechanism 2 (Fig. 5*A*), and proceeds through the formation of structured oligomers. Additionally, we show that the kinetics of Ail aggregation is determined by the physicochemical nature of the interface residue, and the overall process is accelerated by Tyr.

**Figure 6.**
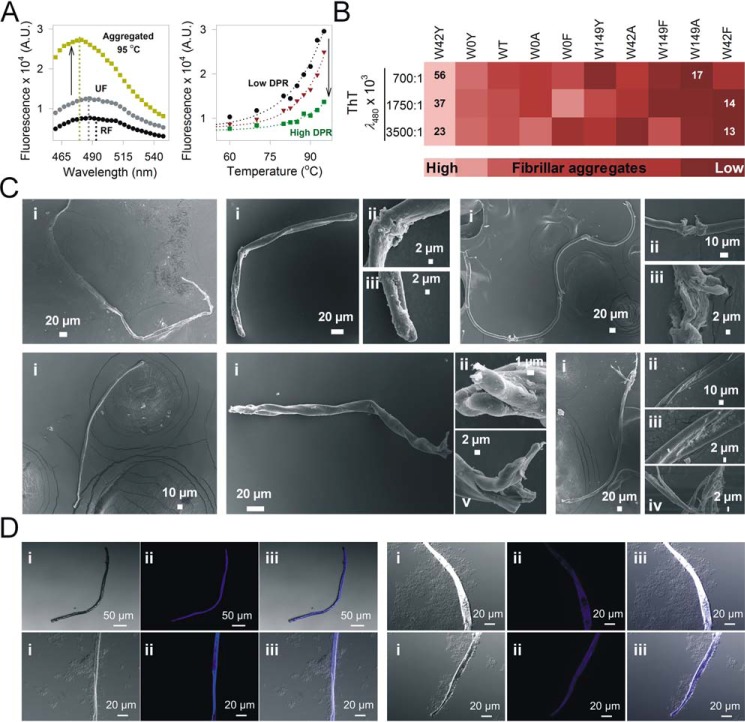
**Fibrillar cross-β aggregate morphology of Ail binds ThT.** *A, left panel*: representative ThT fluorescence emission profiles of 10 μm ThT added to folded (*RF*), unfolded (*UF*), and aggregated Ail-WT. ThT binds β-rich protein aggregates and shows enhanced fluorescence emission (blue-shifted fluorescence with λ_em-max_ ≈ 480 nm), as is seen here for aggregated Ail-WT. The emission spectra are poor in the case of both folded and unfolded proteins, as both proteins bind poorly to ThT. *Right panel*, representative data illustrating the effect of DPR on ThT fluorescence of aggregated Ail at λ_em-max_ = 480 nm. We observe a decrease in ThT fluorescence (*arrow*) upon increasing the DPR (700:1, *black circles*; 1750:1, *red inverted triangles*; and 3500:1, *green squares*). *B,* global comparison of ThT fluorescence for all proteins, in all three DPRs. Mutants are arranged from high (*light shade*, left extreme) to low (*dark shade*, right extreme) ThT fluorescence (complete data in Fig. S33). The high ThT fluorescence represents an increase in fibrillar aggregates in solution. *Numbers* represent the lowest and highest value of ThT fluorescence. *C,* representative SEM images of heat-denatured fibrillar aggregates of folded Ail-WT, at low (*panel i*) and high (*panels ii–iv*) magnification. Ail forms both amyloid-like fibrillar aggregates and amorphous aggregates (see additional images in Figs. S34–S35). *E,* representative images of WT (*left panel*) and W0Y (*right panel*); showing differential interference contrast (*i*), fluorescence (*ii*), and overlay (*iii*) images. Note how only the fibrillar aggregates (and not amorphous aggregates) bind ThT dye (complete data in Fig. S36).

We characterized the final state of Ail aggregates by mapping the morphology of the aggregated samples using ThT dye binding, SEM imaging, and fluorescence microscopy. Ail aggregates exhibit the ability to bind to the amyloid-specific dye thioflavin T (ThT), and show fluorescence emission centered at λ_em-max_ ≈ 480 nm (Fig. 6*A*). Additionally, ThT binding and the measured fluorescence intensity varies across the mutants and correlates well with our aggregation propensity measurements (Fig. 6*B*). Here, the Trp → Ala/Phe mutants, which form fewer fibrillar aggregates, proportionately exhibit lower ThT binding and fluorescence, whereas the highest fluorescence is seen in the Trp → Tyr mutants (Fig. 6*B*; also see Fig. S33). We confirmed the presence of fibrillar morphology, which is the characteristic of amyloid-like β-sheet–rich structures in the aggregates, using SEM (Fig. 6*C*; complete data in Figs. S34 and S35), and fluorescence microscopy (Fig. 6*D*; complete data in Fig. S36). The variations we observe for the imaging and fluorescence measurements of Trp → Phe/Tyr/Ala mutants correlate proportionately with the amount of structured fibrillar aggregates formed by Ail, which, in turn, depends directly on the amino acid at 42^nd^ and 149^th^ positions (note that the initial folded state is similar for all the Trp mutants; Fig. 1*C*). Similar observations have been made for the human mitochondrial voltage-dependent anion channel (VDAC) protein barrel (29).

### Are Ail tryptophans evolutionarily conserved to balance stability and oligomerization?

Our results from aggregation studies of Ail suggests that (i) Ail stability is regulated by kinetic partitioning, which controls Ail unfolding and aggregation; (ii) once aggregation is nucleated, the rate and cooperativity of Ail aggregation dictates the overall aggregation propensity; (iii) chemical nature of the amino acid at 42^nd^ and 149^th^ positions changes the activation energy barrier, controls the aggregation kinetics, and affects the final aggregated state of the protein. Ail also shows an inherent tendency to form amyloid-like β-sheet–rich aggregates, which is similar to other transmembrane β-barrels OmpA (12) and human VDAC (29).

In the cell, biochemical factors such as molecular overcrowding, protein-lipid interaction, and macromolecular dynamics regulate protein folding, stability, and aggregation (31). In addition, in the case of OMPs such as Ail, overexpression changes the effective lipid-protein ratio in the bacterial membrane, and may lead to unfavorable protein association. Hence, we varied the detergent-Ail ratio in our experiments to understand (i) the effect of molecular overcrowding, and (ii) the importance of DPRs on Ail aggregation. Our findings from thermal stability measurements show a detergent concentration-dependent stabilization of Ail barrel, with a proportional increase in thermal stability with increasing detergent content (Fig. S37). In addition, aggregation propensity, aggregation rate, and the extent of formation of fibrous aggregates also decrease with increasing detergent content (see Figs. S33 and S38). Overall, these results suggest important roles of macromolecular overcrowding and changes in the detergent-to-protein ratio or lipid-protein ratios (*in vivo*) as physicochemical factors that promote Ail aggregation. Although it is plausible that our results in pH 8.5 may differ from physiological conditions, the intrinsic ability of Ail to form fibrillar aggregates is retained under various conditions (data not shown). Hence, we speculate that our findings might indeed by relevant physiologically.

Optimal stability, and not high stability, is the ultimate goal of evolution, and this is often associated with the acquisition of new functions (33). By combining our results from stability and aggregation measurements, we demonstrate that Ail intrinsically possesses moderate stability (Fig. 2) and moderate aggregation propensity (Figs. 3 and 4). The latter is also evident from our global stability analysis, where we show that Ail-WT resists unfolding (high *T_m_*_-UF_) (Fig. S39). By measuring the *ME*_215-B/A_ (which measures the extent of protein aggregation) and *P*_sol_ (a measure of the folded protein content after temperature-mediated unfolding), we find that the extent of aggregation is lowest for Ail-WT (Figs. S40–S43). Highly stabilizing (Trp → Ala) and aggregation-prone (Trp → Phe/Tyr) sequences were not favored during evolution, because the Ail-Ala sequences would exhibit lowered folding efficiency at low lipid-protein ratios (see Fig. S5), and the Ail-Phe/Tyr mutants can promote Ail homo-oligomerization in the membrane, which would lower the functional efficacy of the protein. We postulate based on our results that interface tryptophans were evolutionarily selected to balance optimal folding and stability, with structural plasticity and controlled oligomerization of the barrel for its proper functioning in the *Yersinia* membrane. Further studies in this direction will shed new light on the evolutionary selection of the protein primary sequence in bacterial pathogenicity and survival.

## Discussion

Molecular factors responsible for protein stability and protein misfolding or aggregation are important to understand, as they are associated with a variety of debilitating neurodegenerative diseases and amyloidogenesis (1–3, 6, 20). In bacteria, biofilm formation, colonization, targeted binding to host proteins, and pathogenesis are also caused by protein self-association (7, 14, 22–24). The immediate need is to develop newer methods to identify the molecular regulators of the initial stages of protein aggregation, and deduce mechanisms that ultimately culminate in the formation of irreversible cross-β fibrils. The phenomenon of protein aggregation has a common cellular and molecular mechanism (4, 14–16). Yet, the initial events that trigger aggregation *in vivo* cannot be realistically mapped at the atomistic level for a single protein, under physiological conditions. Therefore, *in vitro* measurements that address the outcome of per-residue variations in the protein sequence on aggregation kinetics are essential. Here, we obtain a detailed understanding of the molecular steps involved in protein aggregation and fibril formation using *Y. pestis* Ail as our model system. Ail shows an intrinsic self-oligomerization ability and physicochemical properties that favors its kinetic stability, allowing us to develop spectroscopic and calorimetric thermal perturbation methods to deduce protein aggregation mechanisms. Our results from the thermal perturbation studies of Ail illustrate how kinetic stability, whereas working favorably as a stabilizing factor for OMPs *in vivo*, is additionally a sizeable deterrent in promoting aggregation.

**Figure 7.**
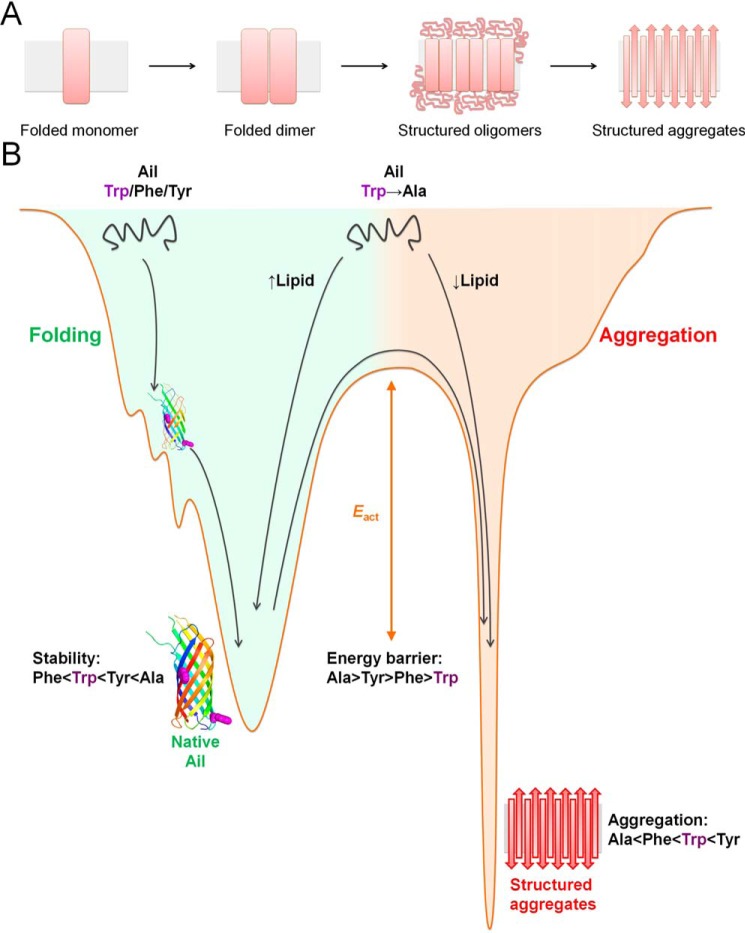
**Understanding the interplay of kinetic stability in the global mechanism of protein aggregation.** *A,* schematic illustrating the proposed mechanism of Ail aggregation. Our findings suggest that folded Ail oligomerizes to directly form structured aggregates without the accumulation of detectable levels of unfolded species. The similarity between the aggregation mechanism for Ail (this study), and the human mitochondrial porin VDAC (29), suggests that OMPs from bacteria and humans might indeed possess similar aggregation pathways. *B,* schematic of the energy landscape depicting kinetic partitioning in Ail folding (*green*, *left*) and aggregation (*red*, *right*). The Trp → Ala mutants show poor folding efficiency in low DPRs, exhibit lipid-dependent folding, and require suitable folding conditions and sufficient [LDAO] for complete folding. Although the Trp → Phe/Tyr mutants are less LDAO-dependent for their folding, the stability of the folded protein is lowest for the Phe variants, whereas the activation energy barrier for aggregation is lowest for the Tyr variants. Overall, tryptophans are preferred at both interface positions in Ail, as the optimal folding efficiency at diverse lipidic environments, moderate stability, and oligomerization propensity conferred by the interface indoles are required to maintain the structural elasticity of the barrel and its metastable scaffold for complete function.

Our findings on Ail aggregation allows us to propose a plausible mechanism of fibrillation in OMPs through structured oligomers as seeding agents (Fig. 7*A*). The availability of sufficient micelles (9–45 micelles per protein) allows us to preclude the association of multiple protein molecules in one micelle. Whether alterations in micelle aggregation number or size cause Ail aggregation is not clear. What is evident from our findings is that Ail retains substantial secondary structure content at higher temperatures. Therefore, the formation of structured oligomers could indicate a loss in protein–micelle interaction prior to protein aggregation. Overall, we find that Ail aggregation proceeds through the formation of structured oligomers (Fig. 7*A*), which give rise to higher order aggregates. We additionally find that interface aromatics, which are abundant in membrane proteins (19), act as local modulators of the magnitude and kinetics of the aggregation event. For the first time, we show that the substitution of a single interface aromatic residue in a membrane protein sequence can substantially alter the stability of the protein's folded scaffold and favor the aggregation pathway (Fig. 7*B*). Trp/Phe/Tyr/Ala show a position-specific effect on Ail stability and aggregation, indicating the likely contribution of local interactions to the nonadditive nature of the mutation (34, 35) (Fig. S1). Additionally, kinetically stabilized protein structures (such as Ail) are both sensitive to mutational effects and are implicated in protein misfolding diseases (2, 6, 11, 30).

To summarize, our study provides five important aspects pertaining to membrane protein aggregation (Fig. 7*B*). (i) Kinetically stabilized proteins are likely to show aggregation. (ii) Physicochemical properties of a specific residue in the protein primary sequence can drive protein aggregation, and modulate the extent of aggregation; this is protein-dependent and does not directly correlate with the hydropathy of the residue. (iii) Aromatic residues show a context-dependent contribution to the stability and aggregation propensity of a membrane protein. In particular, protein stability and aggregation propensity are not necessarily correlated, and can be regulated independently by kinetic partitioning in the protein structure. Furthermore, whereas kinetically stabilized transmembrane β-barrels possess an inherent tendency to form cross-β aggregates, the protein sequence and folding pathway are evolved to minimize such deleterious self-association events during unfolded OMP transport across the periplasm. (iv) We demonstrate how OMPs are excellent tools to study the general mechanism of how β-sheet–rich fibrillar aggregates are formed from partially unfolded protein structures. (v) Our toolbox of 117 spectroscopic thermal perturbation methods provide a highly effective readout to identify molecular elements that cause aggregation in any membrane protein.

Protein sequences with compromised stability, *i.e.* metastable proteins, are often associated with the evolutionary ability to acquire new functions (33). The high thermal stability conferred by Ala (Trp → Ala mutation increases the thermal stability by ∼20 °C and the activation energy barrier of the barrel by ∼10 kcal mol^−1^) may not be preferred in mesophiles such as Ail (33). The evolutionarily need for conserved aromatics at membrane interfaces (despite their lowered stability) might help achieve metastable proteins that have conformational flexibility and the ability to oligomerize without self-aggregation (6, 11, 12). We speculate that the turnover of proteins with moderate kinetic partitioning and moderate activation energy barrier to unfolding should be energetically favorable *in vivo*, and this property may be important for Ail recycling in the *Y. pestis* outer membrane.

The increasing occurrence of neurodegeneration in humans and the likely existence of an evolutionary pressure to balance protein stability and function with turnover highlights the urgent need to investigate biophysical basis for assembly of toxic protein aggregates. For example, the human mitochondrial VDACs co-aggregate with Aβ, Parkin, α-synuclein, Tau, and other amyloidogenic proteins and accelerate the progression of neurodegeneration (29, 36). Despite obvious differences in the protein source, sequence, and microenvironment, we observe similarities in the molecular mechanism of aggregation of *Yersinia* Ail (Fig. 7*A*) and human VDAC (29). Our thermal perturbation tools can readily be applied to other membrane proteins to deduce whether most membrane proteins aggregate to cross-β fibrils through a common mechanism and also identify key molecular contributors for cross-β fibrillization. The availability of molecular details of membrane protein aggregation can open avenues for the design of aggregation blockers, with substantial bearing in the pharmaceutical industry.

## Experimental procedures

### Protein folding

Single and double mutants of the *ail* gene cloned in pET3a vector without the signal sequence were generated using transfer-PCR. All proteins expressed in *Escherichia coli* C41 cells as inclusion bodies, were purified using cation exchange chromatography (34, 37). Ail folding was carried out in LDAO micelles (34). Here, 1400 μm Ail unfolded in 8.0 m GdnHCl, 20 mm Tris-HCl, pH 8.5, was diluted 10-fold into the folding reaction containing 100, 250, or 500 mm LDAO. Following a two-step dialysis over ∼18 h at 25 °C, the sample was diluted 5-fold to achieve a final concentration of 28 μm Ail in a DPR of 700:1, 1750:1, and 3500:1, 20 mm Tris-HCl, pH 8.5, and ∼1.6 mm GdnHCl. Trace amounts of misfolded/aggregated protein were removed by high speed centrifugation (18,500 × *g*). Details are in the supporting “Materials and methods”.

### Electrophoretic mobility shift assay, far-UV CD, fluorescence, and NMR

Complete folding of Ail was established using a electrophoretic mobility shift assay using cold SDS-PAGE and resistance to proteolysis by proteinase K (34, 38). Next, the secondary structure content of all folded samples was measured using far-UV CD (38). Trp fluorescence emission profiles and Trp anisotropy, and average Trp lifetime were additionally recorded to establish Ail folding in LDAO (39). All data were corrected for contributions from LDAO, buffer, and 1.6 mm GdnHCl. Details are described in the supporting “Materials and methods”.

HSQC-TROSY measurements were recorded at 45–75 °C (10 °C increments) using uniformly ^15^N-labeled samples (0.1 mm labeled protein, 175 mm LDAO, DPR = 1750:1), on a 700 MHz NMR spectrometer equipped with a cryoprobe (38). 1024 points in *t1* dimension and 256 *t2* increments were acquired, and data were processed using NMRPipe. Details are described in the supporting “Materials and methods”.

### Thermal denaturation and aggregation kinetics

The folded Ail (28 μm) in a DPR of 700:1, 1750:1, and 3500:1 was used for aggregation studies. Here, the folded protein was denatured by heating, and the change in *ME*_215_, *A*_340_, and *C*_p_ was monitored with increasing temperatures, and various thermal parameters listed in Table 1 were measured. In addition, isothermal unfolding and aggregation kinetics at defined temperatures were measured by monitoring the change in *ME*_215_ to derive *E*_act-ME_. Details are described in the supporting “Materials and methods”.

### Aggregation index calculation

The aggregation index was calculated for the folded protein at various temperatures between 40 and 95 °C using *AI*_340(320)_ = 100 × (*A*_340(320)_/(*A*_280_-*A*_340(320)_)).

### Imaging and characterization of Ail aggregates

ThT binding was monitored for all the aggregated samples obtained post-denaturation. Aggregated samples were also imaged using scanning EM (SEM), differential interference contrast, and fluorescence microscopy. Protein aggregates in buffer and folded proteins were used as controls in each case. Details are described in the supporting “Materials and methods”.

### Global analysis

Data from the 16 unique thermal parameters described in Table 1, and 117 biophysical variables, were analyzed globally. For the global analysis, we first divided all the thermal parameters into subsets on the basis of the phenomenon they describe, and independently normalized each parameter between 0 and 1 for all the mutants. Next, we globally sorted the normalized values in each subset between 0 and 1, and rendered the data as heat maps. Finally, we interpreted the most significant differences, as represented by the heat maps. This global analysis offers the immense advantage that only substantial variations across multiple thermal parameters in large datasets are used to derive meaningful conclusions, such as what we report here from 117 individual biophysical variables. Details are described in the supporting “Materials and methods”.

## Author contributions

A. G. and R. M. data curation; A. G. and R. M. formal analysis; A. G. and R. M. validation; A. G. visualization; A. G. and R. M. writing-original draft; R. M. conceptualization; R. M. supervision; R. M. funding acquisition; R. M. investigation; R. M. methodology; R. M. writing-review and editing.

## Supplementary Material

Supporting Information

## Footnotes

2 The abbreviations used are:

OMP: outer membrane protein
Ail: attachment invasion locus
DPR: detergent-to-protein ratio
*E*_act_: activation energy barrier
*C*_p_: heat capacity values
ThT: thioflavin T
LDAO: *N*,*N*-dimethyldodecylamine *N*-oxide
HSQC: heteronuclear single quantum coherence
TROSY: transverse relaxation-optimized spectroscopy
SEM: scanning electron microscopy
VDAC: voltage-dependent anion channel
GdnHCl: guanidine HCl.
