# Supplementary material for: Single-residue physicochemical characteristics kinetically partition membrane protein self-assembly and aggregation
Source: J Biol Chem. 2019 Dec 16;295(5):1181–94. doi: 10.1074/jbc.RA119.011342 (PMC6996891; doi:10.1074/jbc.RA119.011342)
Supplement: Supporting Information [file supp_295_5_1181__index.html]

Single-residue physicochemical characteristics kinetically partition membrane protein self-assembly and aggregation — Kinetic partitioning in OMP stability and aggregation — Single-residue physicochemical characteristics kinetically partition membrane protein self-assembly and aggregation — Kinetic partitioning in OMP stability and aggregation — Supporting Information 

# Single-residue physicochemical characteristics kinetically partition membrane protein self-assembly and aggregation

## Supporting Information

- Supporting Information (to be published online) - SI Methods and SI Figures.
