# Supplementary material for: Single-residue physicochemical characteristics kinetically partition membrane protein self-assembly and aggregation
Source: J Biol Chem. 2019 Dec 16;295(5):1181–94. doi: 10.1074/jbc.RA119.011342 (PMC6996891; doi:10.1074/jbc.RA119.011342)
Supplement: Supporting Information [file supp_RA119.011342_156256_2_supp_442695_q2cdj2.pdf]

Single-residue physicochemical characteristics kinetically partition membrane protein self-assembly and aggregation

Ankit Gupta[1] and Radhakrishnan Mahalakshmi[1,*]

[1]Molecular Biophysics Laboratory, Department of Biological Sciences, Indian Institute of Science Education and Research, Bhopal – 462066. India.

*Corresponding author: maha@iiserb.ac.in.

SUPPORTING INFORMATION

DETAILED MATERIALS AND METHODS

Cloning, expression, protein preparation, and folding – The *ail* gene was cloned in pET3a vector, without the signal sequence. The single and double Trp mutants W^{42}→F/Y/A, W^{149}→F/Y/A, and $W^{42,149}$→F/Y/A were generated by mutagenesis using transfer-PCR. All the proteins were expressed in *E. coli* C41 cells as inclusion bodies, and purified using cation exchange chromatography, with minor modifications of reported protocols (1,2). The purified protein was used for all the experiments.

Initial folding screens of Ail was carried out from urea-denatured and guanidine hydrochloride (GdnHCl)-denatured states into phosphocholine vesicles (DLPC, 1,2-dilauroyl-*sn*-glycero-3-phosphocholine; DMPC, 1,2-dimyristoyl-*sn*-glycero-3-phosphocholine; and DPPC, 1,2-dipalmitoyl-*sn*-glycero-3-phosphocholine). Here, the unfolded protein (700 – 1400 μM) was diluted 10-fold in vesicles prepared at different concentrations (70 – 140 μM), pH conditions (5.0 – 10.5), temperatures (25 °C – 37 °C), and incubation times (5 min – 48 h) (see Fig. S2-S4 for representative results) and the results were analyzed using electrophoretic mobility shift assay and protease protection assay (described below). However, folding efficiency was poor in all these conditions, and further studies were carried out in N, N-dimethyldodecylamine N-oxide (LDAO) micelles from the GdnHCl–denatured state of the protein.

For all the final spectroscopic experiments, Ail folding was carried out in N, N-dimethyldodecylamine N-oxide (LDAO) micelles, as described previously (1). Here, the unfolded protein (~1400 μM Ail in 8.0 M GdnHCl, 20 mM Tris-HCl pH 8.5) was rapidly diluted 10-fold into the folding reaction, consisting of 100 mM, 250 mM, and 500 mM LDAO prepared in 20 mM Tris-HCl pH 8.5. The folded stock contains ~140 μM Ail in 100 mM, 250 mM, and 500 mM LDAO micelles prepared in 20 mM Tris-HCl pH 8.5 and 800 mM GdnHCl. The folded protein was then subjected to two rounds of 10-fold dialysis against 20 mM Tris-HCl at 25 °C for 12 h and 6 h each, such that the ideal GdnHCl concentration in the reaction would reach 8 mM. The dialyzed sample was diluted 5-fold in LDAO micelles to a final concentration of 28 µM Ail in a detergent-to-protein ratio (DPR) of 700:1, 1750:1, and 3500:1. This corresponds to final detergent concentrations of 20 mM, 50 mM and 100 mM, respectively. The critical micelle concentration (CMC) is a very important parameter in detergent micelles, and the CMC of LDAO is ~1.0 mM. Therefore, all experiments were carried out in LDAO concentrations that were 20–, 50– and 100–fold above its CMC. The final folded stock Ail protein and its mutants were quantified by A_{280} using the respective molar extinction coefficients for each protein, and checked for complete folding using electrophoretic mobility shift (described below). All stock protein preparations were subjected to high-speed centrifugation (18,000 x *g* at 4 °C for 1 h) to remove any trace amounts of protein aggregates formed in the folding process or dialysis steps. Samples before and after dialysis were additionally checked using electrophoretic mobility and protease protection assay, scattering measurements (A_{320}), and aggregation index analysis (AI_{320}) (described later). Comparison of the electrophoretic and spectroscopic parameters ($A_{320} \approx 0$; $AI_{320} \approx 0$) confirmed that the physico-chemical characteristics of folded Ail did not change during sample processing.

Electrophoretic mobility shift assay, far-UV CD, fluorescence, and NMR – To check for complete folding of the samples, five independent methods were used: electrophoretic gel mobility shift assay, protease resistance, far-UV circular dichroism, Trp fluorescence, NMR (HSQC) measurements. In the electrophoretic mobility shift assays, the folded proteins (28 μM folded Ail or its mutants in DPRs of ~700:1, ~1750:1, or ~3500:1) were checked for folding on 15% SDS-PAGE gels using electrophoretic mobility shift assay, as described previously (1,3). Here, unboiled samples exhibit a difference in electrophoretic migration between the folded and unfolded protein, which is used as a reliable indicator of percent folding of Ail. The folding efficiency was estimated using densitometry within each lane as folded band intensity / total intensity of the lane. Samples were also assayed for resistance to proteolysis using proteinase K. Proteinase K is a robust protease that cleaves partially folded, unfolded, or aggregated Ail to small peptide fragments. Hence, we incubated folded samples with 70 μM PK for 1 min. Note that at these PK concentration, the enzyme cannot be inhibited using PMSF. Samples were directly mixed with gel loading dye and checked on 15% SDS-PAGE.

We also measured the secondary structure content and fluorescence properties of folded Ail WT and all the folded Trp mutants at 25 °C. The secondary structure content was measured using far-UV circular dichroism (CD) on a J-815 CD spectropolarimeter (JASCO Inc.), using reported parameters (3). The fluorescence emission spectrum (λ_{em}) and anisotropy (r) values were recorded using FluoroMax 4 spectrofluorimeter (Horiba Jobin Yvon Ltd.), and the average Trp lifetime ($<\tau>$) was measured on a DeltaFlex TCSPC system (Horiba Jobin Yvon Ltd.) (4). Here, the data obtained for the sample was corrected for contributions from LDAO, buffer, and trace GdnHCl (blanks were prepared using all constituents of the sample, namely, buffer, LDAO, and 1.6 mM GdnHCl anticipated in the sample post-dialysis and dilutions). Note that all Ail constructs can tolerate up to 800 mM GdnHCl in the preparation without any change in the folded conformation of the protein and in barrel stability (1,5). Hence, trace amounts of GdnHCl (~1.6 mM) do not interfere in the spectroscopic measurements or sample stability. The final data were compared across the proteins in various DPRs using global analysis (described below).

We also recorded the HSQC-TROSY experiments of the folded uniformly ^{15}N-labeled proteins, on a 700 MHz NMR spectrometer equipped with a cryoprobe, using reported parameters (3). We used a 3.5-fold higher concentration of both the protein and micelles (0.1 mM protein and 175 mM LDAO) without changing the DPR (1750:1) to obtain a good signal-to-noise ratio in the NMR measurement. All NMR experiments were recorded at 45 °C. The data were processed using NMRPipe and plotted using Sparky (3,6). Details are provided in a subsequent section (see below).

Thermal denaturation experiments using far-UV CD – All the CD thermal denaturation measurements were carried out using 10 mm path length, step size of 100 nm/min and data integration time of 1 s, as described previously (5). Briefly, the folded protein was denatured by heating from 4 °C to 95 °C, and the change in molar ellipticity at 215 nm (ME_{215}) was monitored with increasing temperature. The change in ME_{215} was normalized to fraction unfolded (f_U), and the average data from 2–3 experiments were fitted to a sigmoidal function to derive the mid-point of thermal denaturation (T_m). We also calculated the start-point of thermal denaturation and aggregation ($T_{m\text{-start}}$). $T_{m\text{-start}}$ and T_m denote the temperature at which protein unfolding is initiated and 50% complete, respectively, and are indicators of protein stability.

We also measured the extent of the protein aggregation by calculating the ratio of ME_{215} before ($ME_{215\text{-B}}$) and after ($ME_{215\text{-A}}$) thermal denaturation ($ME_{215\text{-B/A}}$), using the following formula:

$$(ME_{215\text{-B/A}}) = (ME_{215\text{-B}}) / (ME_{215\text{-A}})$$

$ME_{215\text{-B/A}}$ represents the extent of secondary structure retained after thermal denaturation, and is used to calculate the extent of protein denaturation and aggregation. As Ail unfolding was not completed even at 95 °C, the end-point temperature of unfolding ($T_{m\text{-end}}$) could not be measured. The CD thermal denaturation measurements were carried out at various ramp rates of 1 °C/min, 5 °C/min, and 10 °C/min to measure the contribution of the kinetic component to Ail stability. All thermal parameters were analyzed globally (described below) to obtain the overall stability and aggregation propensity of Ail.

Thermal denaturation measured using UV light scattering – Scattering measurements were carried out on a TMSPC-8 T_m analysis system using a UV-Vis spectrophotometer 1800 from Shimadzu Scientific Instruments, as described previously (5). Ail folded in various DPRs (700:1, 1750:1, and 3500:1) was heat denatured from 20 – 95 °C, and the change in absorbance due to protein oligomerization and aggregation was measured at 280 nm (A_{280}), 320 nm (A_{320}), and 340 nm (A_{340}). The thermal denaturation profiles were corrected for contributions of LDAO micelles, buffer, and 1.6 mM GdnHCl. Here, the change in absorbance was recorded for buffer/LDAO preparations (20, 50, or 100 mM LDAO containing 20 mM Tris-HCl and ~1.6 mM GdnHCl) from 20 – 95 °C. Measurements obtained from the samples were then corrected for buffer/LDAO contributions across the complete temperature range. Note that we have preferentially used A_{340} for the analysis, as it displays minimal interference from A_{280}. This data was used

to calculate start-point ($T_{m\text{-start-A340}}$), mid-point ($T_{m\text{-A340}}$), end-point ($T_{m\text{-end-A340}}$), and cooperativity of the oligomerization and aggregation ($\Delta T_{m\text{-A340}}$). The $T_{m\text{-A340}}$ was calculated by fitting the thermal denaturation profiles to a sigmoidal function, whereas the cooperativity or rate of the oligomerization and aggregation was calculated as $\Delta T_{m\text{-A340}} = T_{m\text{-end-A340}} - T_{m\text{-start-A340}}$ (see schematic in Fig. 3A). Additionally, we measured the difference in the absolute absorbance at the final and initial temperatures (ΔA_{340}) to obtain the extent of aggregation (see schematic in Fig. 3A). At least 2–3 independent scattering measurements were carried out at different ramp rates (0.5 °C/min, 1.0 °C/min, 2.0 °C/min, and 5.0 °C/min) for all the proteins, and the standard deviation was derived from the data. The thermal parameters thus measured were used for global analysis (described below) to obtain the stability and aggregation propensity of Ail.

Isothermal unfolding coupled aggregation kinetics using far-UV CD – Isothermal unfolding and aggregation kinetics were measured using far-UV CD, as described previously (5). Briefly, folded samples in various DPRs were incubated at various pre-set temperatures between ~40 °C – 95 °C and the loss in ME_{215} upon protein unfolding and aggregation was measured at various temperatures. The change in ME_{215} was then fitted to single exponential rise function, and the combined rate for unfolding and aggregation was calculated. The observed rates were plotted against temperature, and the activation energy ($E_{act\text{-}ME}$) was calculated by fitting the data to a polynomial function using the equation: slope = $E_{act\text{-}ME}$ / R, where R = gas constant. The $E_{act\text{-}ME}$ for various mutants in varying DPRs were compared globally across the various Ail Trp mutants (described below).

Calculation of aggregation index using UV absorbance spectroscopy – To derive the aggregation index, the folded protein at different DPRs was heated for 10 min at various temperatures between ~40 °C – 95 °C, to induce aggregation. Heat-denatured samples were allowed to cool gradually to 25 °C, and the absorbance at 280 nm (A_{280}), 320 nm (A_{320}), and 340 nm (A_{340}) was recorded, and used to calculate the aggregation index at 320 nm (AI_{320}) and 340 nm (AI_{340}) using the formula:

$$AI_{340} = 100 \text{ x } (A_{340} / (A_{280} - A_{340}))$$
$$AI_{320} = 100 \text{ x } (A_{320} / (A_{280} - A_{320}))$$

Note that we have preferentially used AI_{340} for the analysis, as A_{340} displays minimal interference from A_{280}. Also note that AI calculation measures the monomers, oligomers, and the aggregated samples at various temperatures, across the proteins, and represents the overall aggregation propensity.

Additionally, the amount of stably folded sample in the solution (P_{sol}) was also determined. For this, aggregated protein was removed by high speed centrifugation at 18,000 x *g* at 4 °C for 1 h, and protein recovered in the soluble fraction was quantified as P_{sol}. Here, a higher value for P_{sol} indicates a highly stable protein with low aggregation propensity. All the samples (before and after centrifugation) were also checked using SDS-PAGE for sample integrity (data not shown).

Differential scanning microcalorimetry (DSC) – We carried out DSC measurements to study thermal stability and aggregation of Ail and its Trp mutants using MicroCal VP-DSC System from Malvern Analytical Ltd.. Folded Ail at various DPRs were heated from 20 °C – 120 °C at low gain mode and a constant filtering period of 10 s, at a varying ramp rate of 0.5 °C/min, 1.0 °C/min, and 1.5 °C/min. Ail heat capacity curves show contributions from both denaturation/unfolding (endothermic transition) and subsequent aggregation (exothermic transition) processes. Such aggregation events occlude the direct interpretation of enthalpies from specific (molar) heat capacity values (C_p). The unfolding and aggregation profiles obtained were therefore corrected for LDAO and buffer contributions, and the raw C_p values for both the exothermic and endothermic processes were compared across all proteins. As Ail shows irreversible thermal denaturation, our thermodynamic analysis involved the calculation of mid-point temperature of unfolding ($T_{m\text{-UF}}$) and aggregation temperature ($T_{m\text{-Agg}}$). Here, the endothermic and exothermic transitions were analyzed separately to understand unfolding and aggregation, respectively.

The thermal parameters measured ($T_{m\text{-}UF}$ and $T_{m\text{-}Agg}$) were compared globally at various temperature ramp rates and DPRs, across the various mutants.

Assessment of Ail conformation using NMR spectroscopy – HSQC-TROSY (heteronuclear single quantum coherence – transverse relaxation optimized spectroscopy) measurements of the uniformly ^{15}N-labeled folded Ail was carried out at a constant DPR of 1750:1 (0.1 mM protein and 175 mM LDAO), on a Bruker AVANCE- III 700MHz FT NMR spectrometer equipped with a 5 mm TCI cryoprobe (3). All samples were first subjected to high-speed centrifugation (18,000 x *g* at 4 °C for 1 h) to remove possible aggregates formed in the folding reaction. Samples additionally contained 10% D_2O for frequency locking. For data acquisition, 1024 points from 256 scans in the *t2* and 256 increments in the *t1* dimensions were used. Data was acquired from 45 °C – 75 °C in 10 °C increments using identical parameters to ensure that the obtained S/N was directly proportional to the absolute concentration of the folded protein in solution. The data were processed using NMRPipe using a linear prediction applied to *t1* to 512 points, and data were plotted using Sparky (3,6).

Characterization of Ail aggregates using thioflavin T fluorescence – Aggregated protein samples of various Ail Trp mutants obtained after isothermal unfolding were checked for their efficiency to bind thioflavin T (ThT) dye, as described previously (5). Briefly, protein aggregates were generated by heating the folded proteins at ~40 °C – 95 °C for 10 min. Aggregated samples were then diluted 3.5-fold with 20 mM LDAO prepared in Tris-HCl pH 8.5, and ThT was added to a final concentration of 10.0 μM. Samples were incubated at 25 °C for 15 min, and ThT fluorescence was recorded using a $\lambda_{ex\text{-}max}$ = 430 nm and λ_{em} = 460-550 nm, at a fixed slit width of 5 nm. The fluorescence emission maximum of ThT changes upon binding to the aggregated protein. Additionally, the ThT fluorescence of unfolded (in 8 M GdnHCl) and folded (in LDAO), as well as aggregated proteins (lyophilized protein powder in the buffer) were also recorded as negative and positive controls, respectively. The fluorescence spectrum of all the samples was corrected for LDAO and buffer contributions, and the corrected data was used for analysis. Here, the ThT fluorescence at 480 nm was compared globally across the Ail Trp mutants, to obtain information on the amount of β-sheet rich fibrillar aggregates.

Scanning electron microscopy (SEM) of Ail aggregates – SEM imaging was done using a high resolution field emission scanning electron microscope (Zeiss UltraPlus FE-SEM), as described previously (5). Aggregation of the protein folded in LDAO micelles was induced by heating the sample at 95 °C for 10 min. The aggregated sample was drop coated onto SEM stubs and allowed to air dry overnight at 25 °C, followed by drying under a nitrogen stream for 10 min. Samples were gold coated using a Q150R rotary-pumped sputter coater instrument (Quorum Technology), and imaged using an accelerating voltage of 10 kV. SEM images were also obtained for the protein aggregates in buffer, to confirm that the observed aggregates are not an artifact of heating or LDAO.

DIC and fluorescence microscopy of Ail aggregates – The heat-induced aggregates of Ail (prepared by heating the folded sample at 95 °C for 10 min) were incubated with ThT at a final of 10.0 μM, at 25 °C for 15 min. Samples thus stained were centrifuged at 18,000 x *g* at 4 °C for 15 min and the pellet faction containing the aggregated protein was washed once with 20 mM Tris-HCl pH 8.5. 10 μl of the washed samples was transferred on a glass slide, mounted with a coverslip, and used for imaging. We used bright field and fluorescence channel (DAPI and Calcofluor white 2MR filters) to acquire the image of ThT–bound Ail aggregates, on an Axio Imager M2 microscope (Zeiss, Jena, Germany) equipped with an oil-immersion objective.

Global analysis – Global analysis was performed by selecting 16 unique thermal parameters described in Table 1, and 117 biophysical variables. Global analysis was first calculated for the DPR of 1750:1 and a temperature ramp rate of 1.0 °C/min. First, the measured thermal parameters were divided into subsets on the basis of the phenomenon they describe. For example, (i) $T_{m\text{-}start}$, T_m, and E_{act} derived

from ME_{215} and A_{340} are a measure Ail stability; (ii) $T_{\text{m-end}}$, ΔT_{m}, ΔA_{340}, and AI_{340} calculated using A_{340}, as well as $T_{\text{m-Agg}}$ derived using C_{p} indicate the aggregation propensity of the folded barrel; (iii) ΔT_{m} monitors the rate of oligomerization or aggregation; (iv) $ME_{\text{215-B/A}}$ and P_{sol} calculated using A_{340} measures the extent of protein aggregation. After segregating all the thermal parameters, the global analysis was done as follows:

1. All thermal parameters were independently normalized between 0 and 1 for all the mutants. Here, 0 represents the lowest stability as well as highest aggregation – both being measures of properties less favored in the protein; 1 represents highest stability and lowest aggregation.
2. We globally sorted the normalized values of various thermal parameters in each subset from lowest to largest. The sorted data were color coded to generate the heat maps.
3. The most significant differences, represented by the mutants present at the leftmost and rightmost extremes of the heat maps, were used for interpretation of the results.

This analysis allows for a direct comparison of only the major differences that are statistically significant, thereby providing reliable conclusions, and ignores minor variations in the thermal parameters in large datasets such as what we report here from 117 individual biophysical variables. The global analysis was then independently applied to data obtained from other DPRs and ramp rates.

SUPPLEMENTARY FIGURES

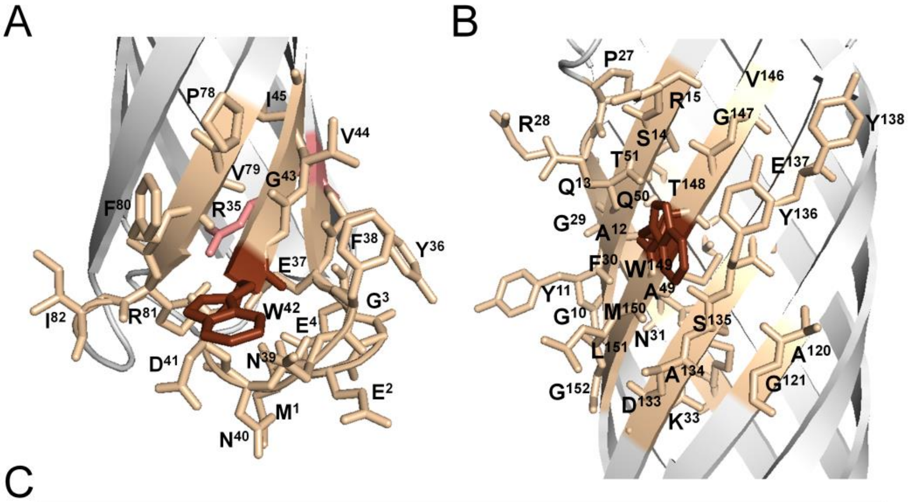

Tryptophan	Residues present in 8 Å vicinity
W^{42}	M^{1}, E^{2}, G^{3}, E^{4}, R^{35}, Y^{36}, E^{37}, F^{38}, N^{39}, N^{40}, D^{41}, G^{43}, V^{44}, I^{45}, P^{78}, V^{79}, F^{80}, R^{81}, I^{82}
W^{149}	G^{10}, Y^{11}, A^{12}, Q^{13}, S^{14}, R^{15}, P^{27}, R^{28}, G^{29}, F^{30}, N^{31}, K^{33}, A^{49}, Q^{50}, T^{51}, A^{120}, G^{121}, D^{133}, A^{134}, S^{135}, Y^{136}, E^{137}, Y^{138}, V^{146}, G^{147}, T^{148}, M^{150}, L^{151}, G^{152}

Figure S1. Residues in the vicinity of the two tryptophans of Ail. Cartoon representation of Ail structure (grey ribbons), with residues in the 8 Å vicinity of W^{42} and W^{149} (dark brown) shown in (A) and (B), respectively, in pale yellow. Both tryptophans form an interaction network with charged, polar, and hydrophobic residues. (C) Table summarizing the residues present in the 8 Å vicinity of W^{42} and W^{149}. Residues buried within the membrane are shown in green, residues in the extra-membranous region are shown in blue, and residues at the interface are shown in black. In all three panels, residues are represented using the single-letter abbreviation of each amino acid, and the residue number is shown as superscript. Note here that in order to maintain simplicity and clarity, the polar lipid/detergent headgroups are omitted from the schematic in (A) and (B). Both tryptophans are lipid solvated in their folded state and reside at the membrane interface. Hence, both indoles primarily establish interactions with the apolar lipid tail and the lipid headgroup. Also note that there are several charged and polar residues in the 8 Å vicinity of both indoles. However, the stereo-spatial geometry of Asp, Glu, and Arg side chains energetically favors interactions with the lipid/detergent headgroup in Ail, and these residues are less likely to form intra-protein side chain – side chain interactions.

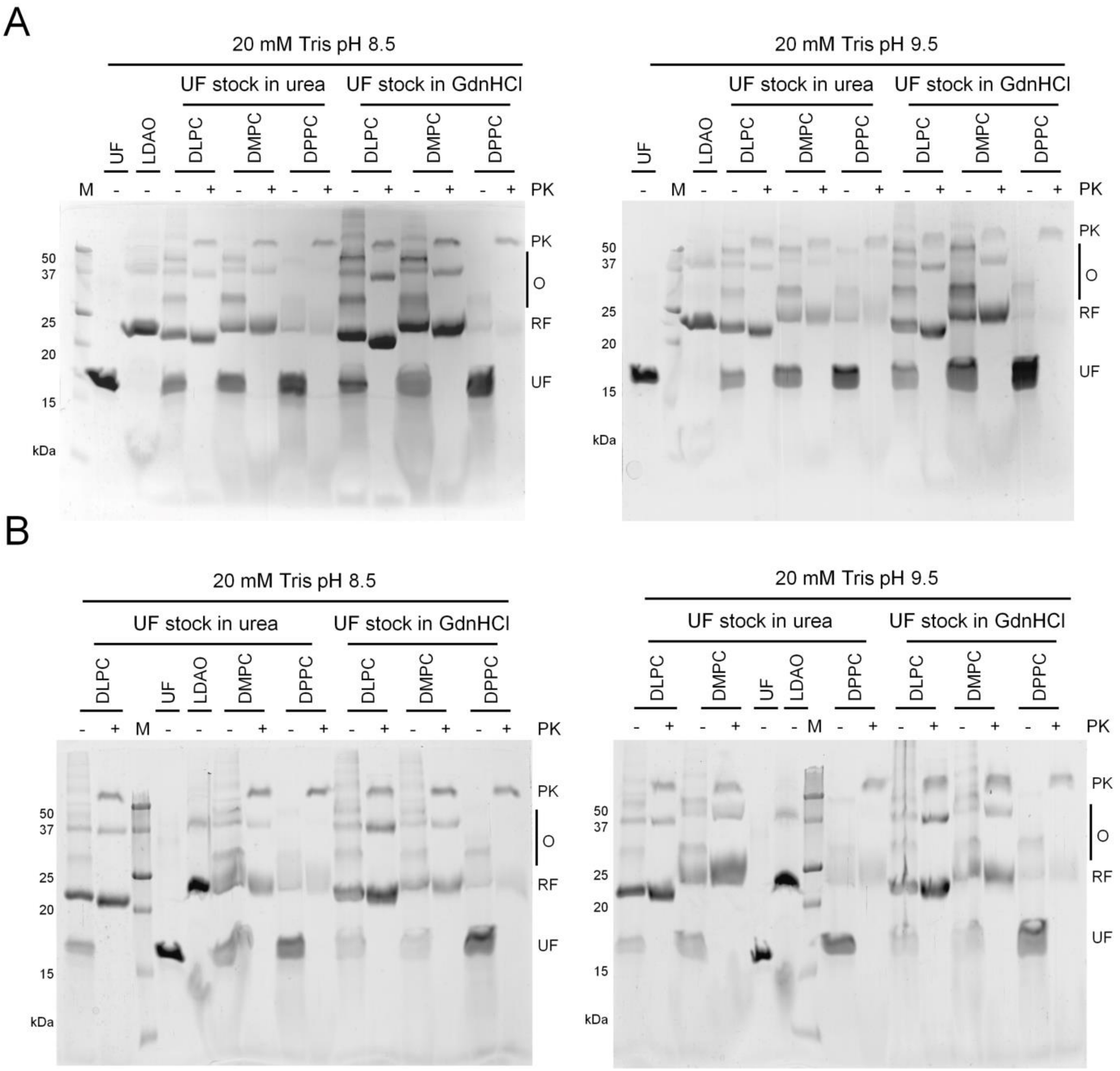

Figure S2. Folding screens of Ail–WT in phosphocholine vesicles. Representative SDS-PAGE profiles of folding screens for Ail–WT in DLPC, DMPC, and DPPC vesicles. Ail aggregates when folded in acidic or neutral pH (data not shown); therefore, folding reactions were screened in alkaline pH (20 mM Tris-HCl at pH 8.5 (left panel) and 20 mM Tris-HCl at pH 9.5 (right panel)). Here, folding was carried out by rapid dilution of 1400 µM unfolded Ail (UF stock) prepared in 8 M urea (UF stock in urea) or 8.0 M guanidine hydrochloride (UF stock in GdnHCl), containing 20 mM Tris-HCl at pH 8.5 or 9.5 into the folding reaction. The UF stock was diluted 10-fold into the folding reaction containing 15 mM DLPC, 18 mM DMPC, or 18 mM DPPC vesicles prepared in 20 mM Tris-HCl at pH 8.5 or 9.5, at 25 °C. The folded samples were incubated at 25 °C for 15 min (A) or 20 h (B), and checked for folding efficiency using electrophoretic mobility on cold SDS-PAGE gels (1,3). Folded Ail exhibits retarded mobility when unboiled samples are separated on Laemmli gels, and this folded protein is resistant to proteolysis by robust proteases such as proteinase K (PK) (1). Protein folded in lauryldimethylamine-N-oxide (LDAO) was used as the positive control, whereas Ail in 8 M urea (without LDAO or vesicle) served as the unfolded (UF) control. Ail-vesicle samples were further treated with 70 µM PK (+) or PK buffer (–) and separated on 15% SDS-PAGE gels. Note that all lipidic conditions do not support complete folding of Ail, resulting in differences in the electrophoretic migration of the folded band. Such incomplete folding

of the barrel also substantially increases the population Ail oligomers and the susceptibility of the folded protein to proteolysis. Overall, we observe that the folding efficiency of Ail is between 30-70% in phosphocholine vesicles, which is evident from the PK-susceptible unfolded band in all the samples. M: molecular weight marker; PK: proteinase K; RF: folded monomer; UF: unfolded protein; O: oligomers.

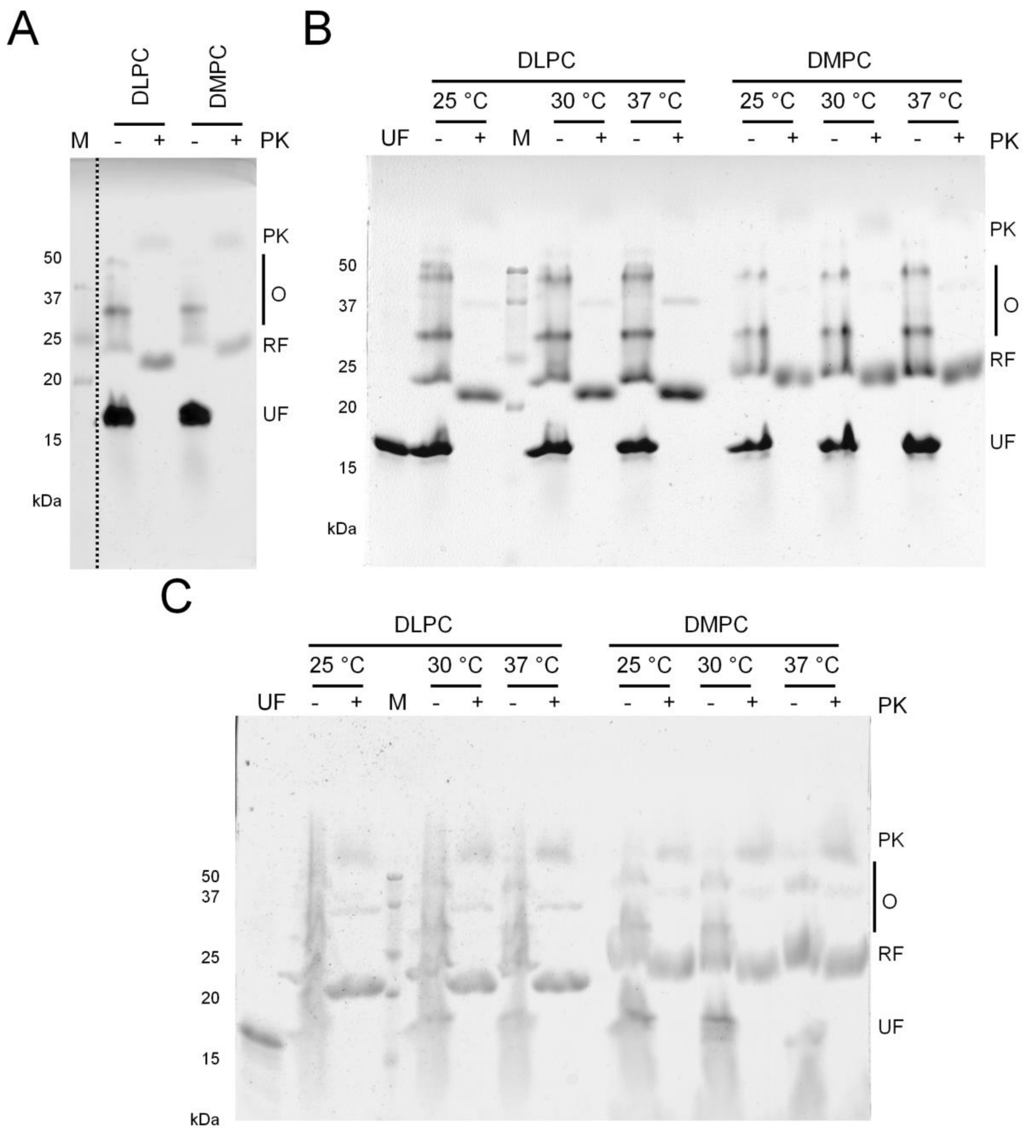

Figure S3. Folding screens of Ail–WT at various temperatures. Representative SDS-PAGE profiles of folding screens for Ail–WT in DLPC and DMPC vesicles. Folding was carried out by a rapid 10-fold dilution of 1400 µM unfolded Ail (in 8.0 M GdnHCl prepared in 20 mM Tris-HCl pH 8.5) into the folding reaction containing 15 mM DLPC or 18 mM DMPC vesicles. The buffer and lipid conditions were chosen based on the results obtained in the previous screens (Fig. S2). The samples were incubated at 25 °C, 30 °C, or 37 °C for 15 min (A), 24 h (B) and 48 h (C) and checked for folding efficiency using electrophoretic mobility (1). All samples treated with 30 µM PK (+) or PK buffer (–) were separated on 15% SDS-PAGE, without boiling. As the folding efficiency was affected by the incubation temperature, PK concentrations were proportionately lowered in the reaction. As all lipidic conditions do not support complete folding of Ail, differences in the electrophoretic migration of the folded band, increase in the population Ail oligomers, and increased susceptibility of the folded protein to proteolysis is seen. Overall, we observed incomplete folding of Ail at all temperatures and in all phosphocholine vesicle preparations (note the presence of the PK-susceptible unfolded band in all the samples). Furthermore, we also checked Ail folding by varying the pH and lowering the protein concentration (data not shown), but Ail did not exhibit complete folding in phosphocholine vesicles, in all conditions screened. M: molecular weight marker; PK: proteinase K; RF: folded monomer; UF: unfolded protein; O: oligomers.

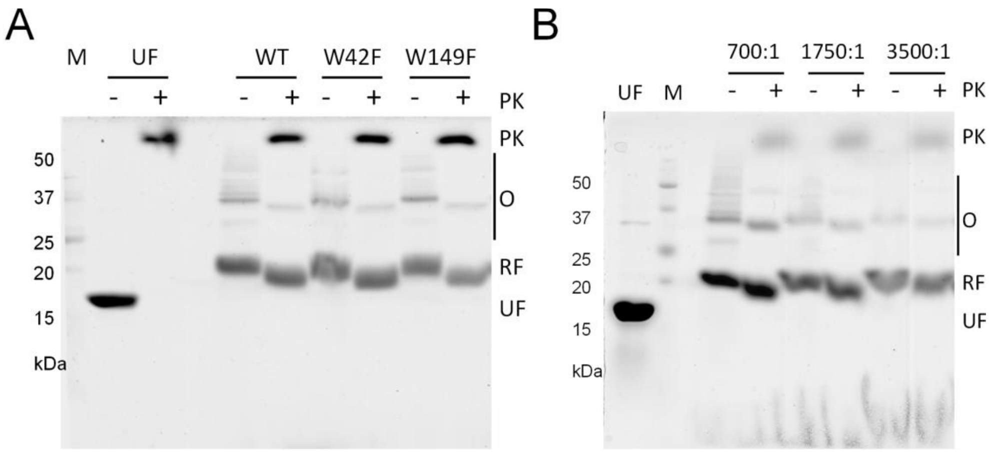

Figure S4. Gel mobility shift confirms Ail folding in LDAO. (A) Representative cold SDS-PAGE gel profiles of Ail–WT, W42F and W149F folded in a detergent-to-protein ratio (DPR) of 700:1. The final concentration of Ail in each sample is 28 μM. (B) Representative SDS-PAGE profiles Ail–WT folded in varying DPRs of LDAO. Unfolded control (UF) prepared in 8 M urea was also loaded for comparison. The final concentration of Ail in each folded sample is 28 μM, 14 μM, and 8 μM in the DPRs of 700:1, 1750:1, and 3500:1, respectively (in the gel). The folded samples were treated with 70 μM PK in (A) and 30 μM PK in (B) (+) or PK buffer (–) and loaded without boiling. All Ail samples show complete folding, and exhibit retarded gel mobility upon folding. In all conditions, the folded protein is also resistant to PK digestion. The results show that Ail–WT and its Trp mutants show complete folding in LDAO micelles and the Trp substitution does not impede folding efficiency of Ail. Ail has an intrinsic tendency to oligomerize, and we obtain ~20% oligomeric species in Ail folded in LDAO micelles. Note that all the proteins show similar % oligomerization, and the oligomerization tendency is not affected by Trp substitution. M: molecular weight marker; PK: proteinase K; RF: folded monomer; UF: unfolded monomer; O: oligomers.

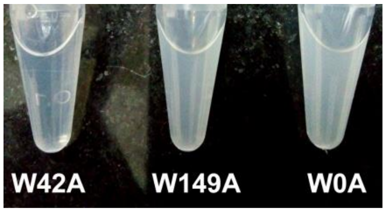

Figure S5. Selective Ail Trp→Ala substitutions affect folding efficiency only in low DPR of LDAO. Representative images of the folding reaction of Ail W42A (left panel), W149A (middle panel), and W0A (right panel), at a constant DPR of 700:1. Interestingly, only W149A and W0A show lowered folding efficiency and visible precipitation in low DPRs of LDAO. Due to aggregation, we observe some loss in the effective protein concentration in the folded sample. W42A retains higher folding efficiency even in low DPRs, suggesting the effect of selective substitution at position 149 to Ala, as the primary source of the lowered folding efficiency of Ail. At high DPRs of 1750:1 and 3500:1, both W149A and W0A exhibit complete folding, without any detectable aggregation (discussed later).

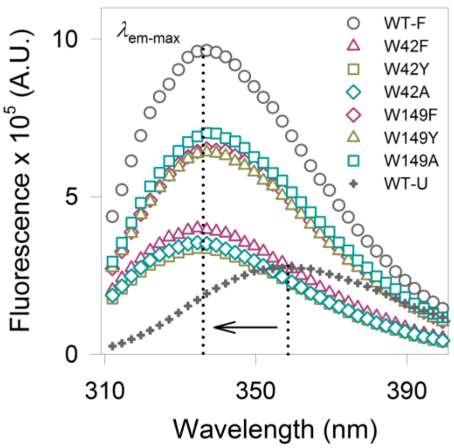

Figure S6. Folded Ail–WT and its Trp mutants show signature fluorescence emission profiles. Representative fluorescence emission profiles of folded Ail–WT (WT-F) and its Trp mutants folded in LDAO micelles at a DPR of 1750:1 and a constant protein concentration of 28 μM. The emission spectrum of unfolded WT protein (WT-U) is also shown for comparison. The folded proteins show a $\lambda_{em\text{-}max} \approx 336$ nm, whereas the unfolded protein shows a red-shifted emission centered at $\lambda_{em\text{-}max} \approx 360$ nm (dotted drop lines). This result also suggests that Ail–WT and its Trp mutants show complete folding in LDAO micelles. Notably, the fluorescence emission intensity is different for the W42X and the W149X mutants of Ail (1). Here, the W42X mutants (which retain W149) show lowered fluorescence emission intensity because of the conformational flexibility of W149 which increases non-radiative losses. On the other hand, W42 in the W149X mutants resides in a rigid local environment and shows higher radiative losses, giving rise to higher fluorescence intensity in the emission spectra.

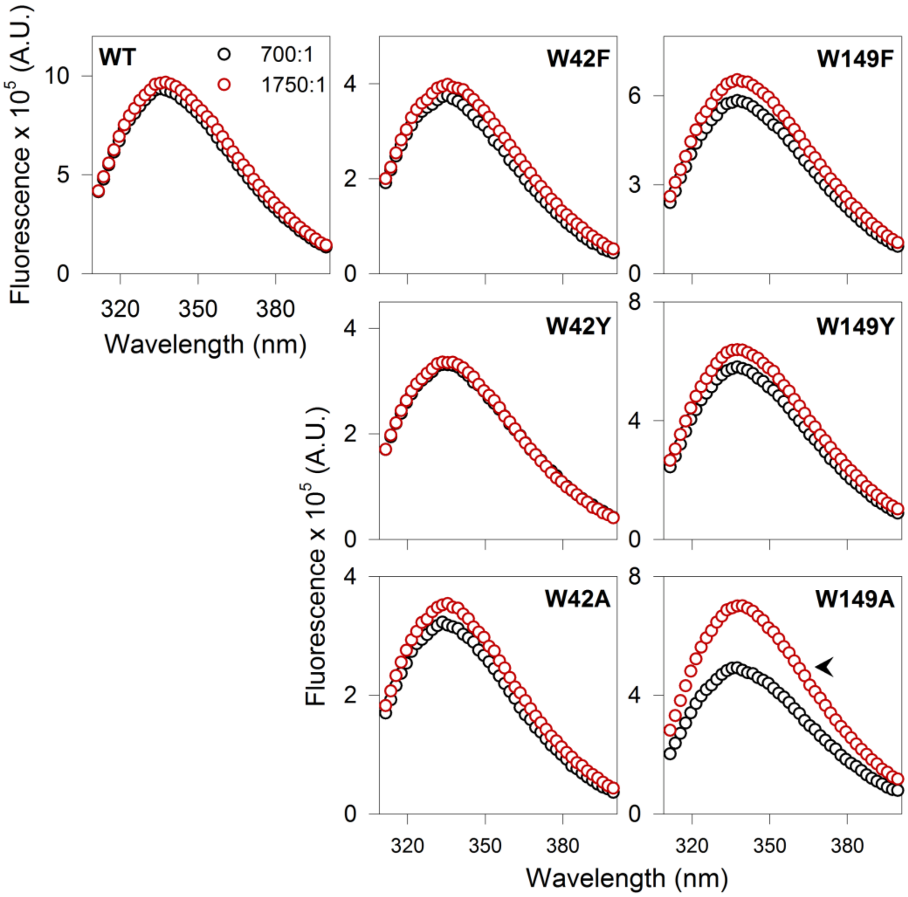

Figure S7. Spectral properties of folded Ail is unaffected by the change in DPR. Representative fluorescence emission profiles of Ail–WT and various Trp mutants folded in LDAO micelles (DPR = 700:1 (black) and DPR = 1750:1 (red)). All the folded proteins show a $\lambda_{em\text{-}max} \approx 336$ nm in both the DPRs. Further, except for W149A, all the Ail variants exhibit similar fluorescence intensity in both the DPRs. The lower fluorescence intensity observed for W149A in DPR = 700:1 is due to the poorer folding efficiency and aggregation of the protein in this condition (see Fig. S5). The difference in fluorescence intensity of all the W42X and W149X mutants is an inherent property of Ail, and is observed due to differences in the local environment of both the tryptophans (see Fig. S6). However, note that the nature of the mutation at position 42 has no effect on the fluorescence property of W^{149}; similarly, the mutation at position 149 does not affect the fluorescence emission spectrum of W^{42}.

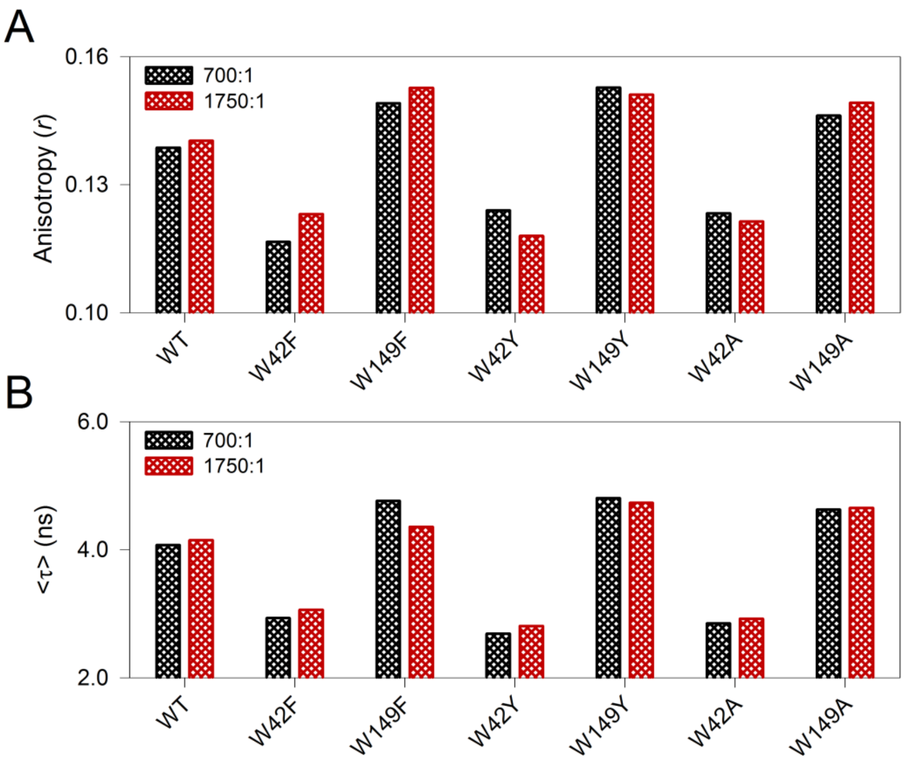

Figure S8. Anisotropy and lifetime measurements confirm complete folding of Ail in all DPRs. Comparison of (A) anisotropy (*r*), and (B) average lifetime ($<\tau>$) values of various Ail Trp mutants, folded in LDAO (shown here are two representative DPRs of 700:1 (black) and 1750:1 (red) used in this study). Ail–WT and all the Trp mutants fold efficiently in LDAO micelles and show Trp-specific spectral properties. A change in the DPR does not affect the spectral properties of folded Ail.

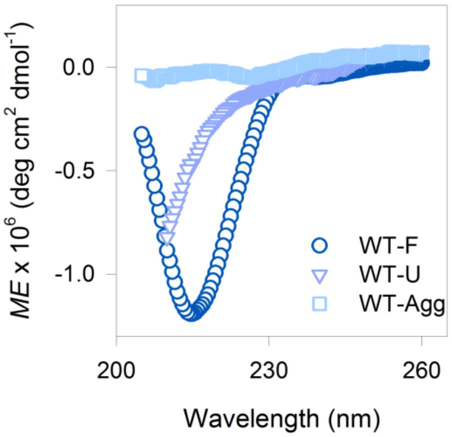

Figure S9. Folded Ail exhibits the anticipated β-sheet structure in CD measurements. Representative far-UV CD (circular dichroism) profiles of folded (F), unfolded (U) and aggregated (Agg) samples of Ail–WT. The folded protein was prepared in a DPR of 700:1, the unfolded sample was prepared in 8.0 M urea, and the aggregated protein was prepared in 20 mM Tris-HCl buffer pH 8.5 containing 400 mM urea. Folded Ail shows the signature negative exciton maximum at 215 nm in far-UV CD measurements, confirming that the protein adopts a β-sheet structure upon folding. This result, along with the electrophoretic mobility shift measurements (Fig. S4), fluorescence emission profiles (Figs. S6 and S7), as well as Trp anisotropy and lifetime measurements (Fig. S8) together confirm that Ail is well-folded and adopts a β-barrel structure in LDAO micelles.

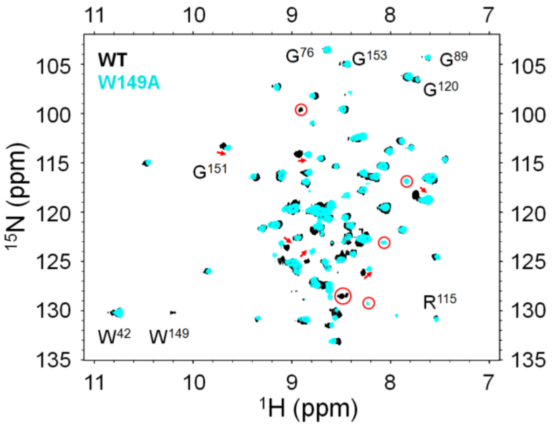

Figure S10. Heteronuclear NMR experiment measurements further confirm Ail folding in LDAO micelles. (A) Overlay of ^{1}H-^{15}N HSQC-TROSY spectra of Ail–WT and Ail W149A, folded in LDAO micelles at a DPR of ~1750:1. We specifically chose the W149A mutant for comparison, as it shows the poorest folding efficiency. HSQC spectra were recorded at 45 °C (as Ail does not show a well-dispersed HSQC spectrum at 25 °C). Well-dispersed spectra with superposed resonances for both the proteins confirm complete folding of Ail–WT and W149A in LDAO. Some of the resonances are annotated from previous reports (7). Minor spectral shift observed in few resonances (indicated by arrows), as well as the emergence of a few new resonances (circled) are a likely consequence of changes to the local environment as a consequence of the mutation. Overall, a well overlapping spectrum for both the proteins suggests that the barrel structure is unaffected by the mutation, and all the proteins are folded to a similar extent. Note that the data used here is the same as shown in Fig. 5C of the main text.

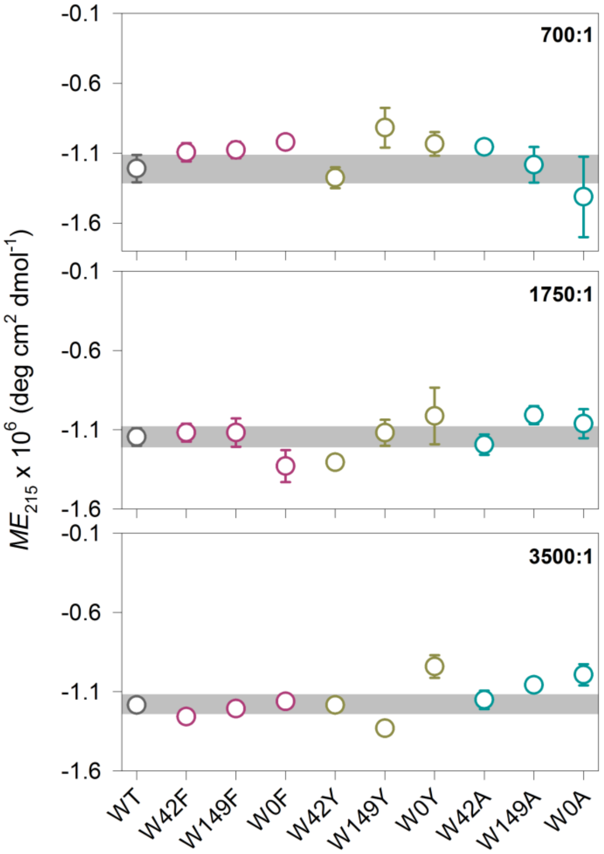

Figure S11. Ail folds at all DPRs, and the folding is not affected by Trp substitution: Inference from secondary structure measurements using far-UV CD. Comparison of secondary structure content at 215 nm (ME_{215}) of Ail–WT and the Trp mutants (28 µM) folded in various DPRs (700:1 (upper panel), 1750:1 (middle panel), and 3500:1 (bottom panel)) of LDAO. A rainbow color pattern is used here: WT (gray), Phe mutants (pink), Tyr mutants (yellow), and Ala mutants (cyan). In all plots, errors are s. d. derived from a minimum of 2–3 independent experiments, except the data for W42F and W149F in a DPR of 3500:1 which are single datasets. The shaded area in all plots represents the s. d. obtained for ME_{215} of Ail–WT. Comparable ME_{215} values across all mutants suggest similar folding efficiency for all Ail variants. The data also show that both Trp substitution and DPR do not affect the secondary structure content and folding efficiency of Ail. Note that the data used here is the same as Fig. 1C of the main text.

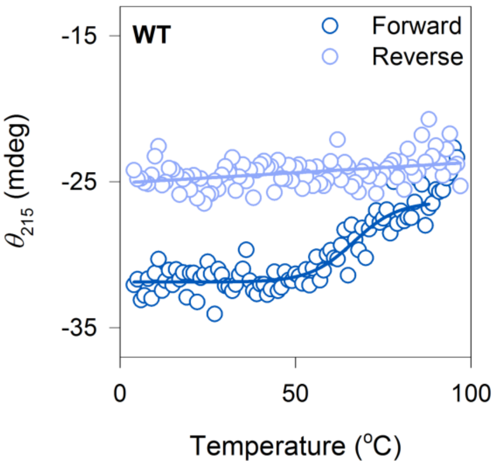

Figure S12. Ail shows irreversible thermal denaturation: Inference from thermal denaturation measurements using far-UV CD. Representative thermal denaturation profile of Ail–WT folded at a DPR of 700:1. The folded sample was heated from 4 °C – 95 °C (forward melting: dark blue) following which the sample was cooled down from 95 °C – 4 °C (reverse melting: light blue). The temperature was increased or decreased at a constant ramping rate of 1 °C/min. We observe a decrease in the raw ellipticity at 215 nm (θ_{215}) with increase in temperature; the ellipticity (which is an indicator of the secondary structure content) does not show recovery upon cooling the sample to 4 °C. We conclude from this result that Ail shows irreversible thermal denaturation. The unfolded protein does not refold upon cooling the sample, and instead undergoes aggregation upon heating.

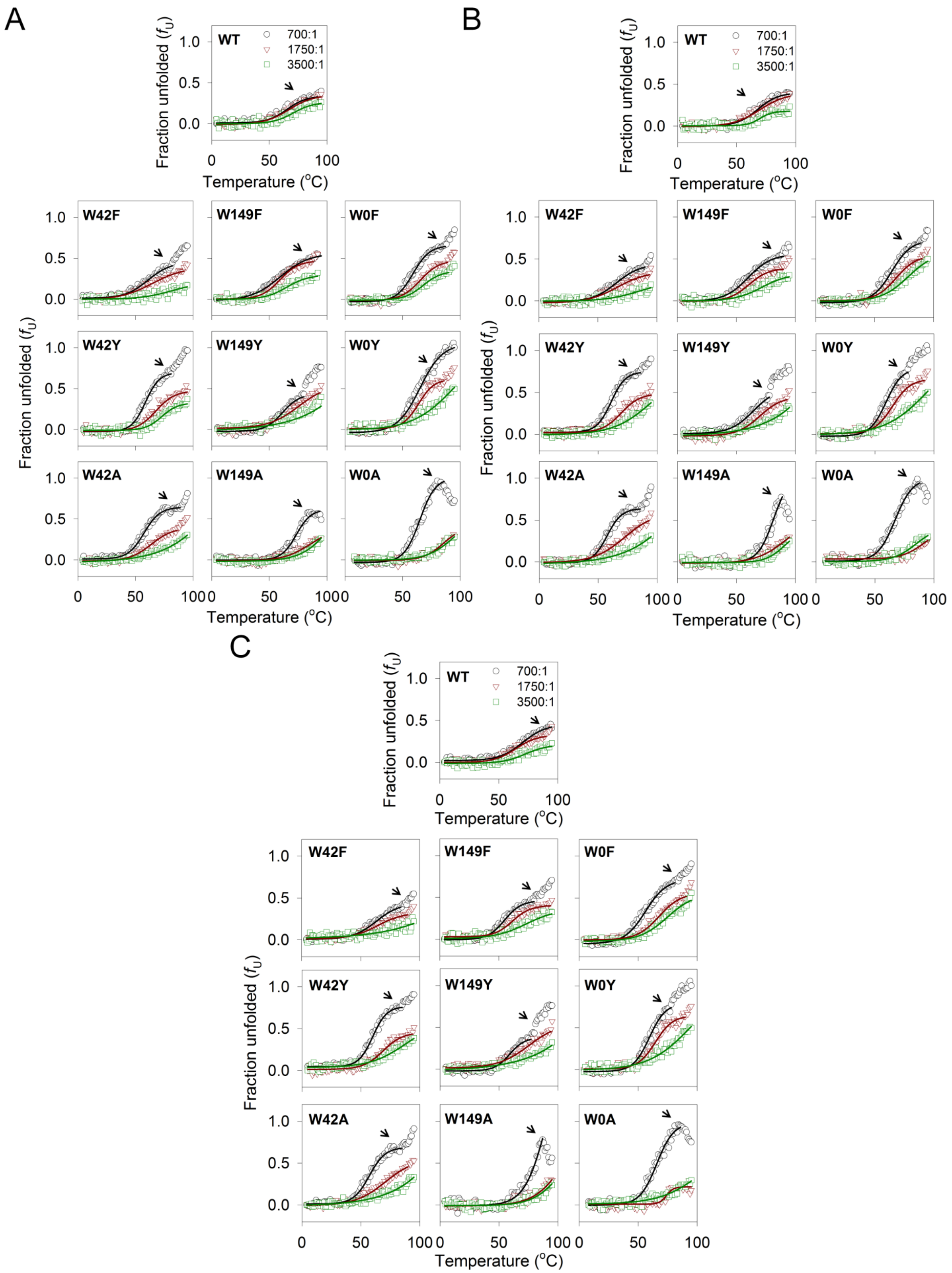

Figure S13. Ail shows deviation from two-state unfolding profiles at low DPR: Inference from thermal denaturation measurements using far-UV CD. Thermal denaturation profiles of Ail Trp mutants, at a temperature ramp rate of (A) 1 °C/min, (B) 5 °C/min, and (C) 10 °C/min monitored using

far-UV CD. To allow for direct comparison of the thermal curves across the three DPRs (700:1, black; 1750:1, red; and 3500:1, green), we have normalized the ME_{215} to fraction unfolded (f_U) values. W42Y showed the highest unfolding, as gauged by the loss in the ME_{215} value. Hence, the ME_{215} of W42Y in 20 mM LDAO at 95 °C was taken as the reference for completely unfolded protein to calculate f_U for all the proteins. The data shown here is the mean of 2–3 independent experiments, except for WT, W42F, and W149F at DPR of 3500:1, wherein representative data are shown. The error bars are hidden for clarity. The mean data were fitted to a sigmoidal function to derive the T_m (see Fig. S16 for the T_m values), and fits are shown as solid lines in each graph. Increasing the DPR lowers the extent of thermal denaturation (f_U) for all the proteins. Additionally, at higher DPRs, we observe a two-state unfolding profile for all the proteins. Interestingly, at low DPRs, we observe a deviation from the typical two-state thermal denaturation profile (indicated by a black arrow in all the graphs). Presence of such non-two-state profiles points to the existence of a transient unfolding intermediate in the Ail unfolding pathway. We also observe a decrease in f_U for W149A and W0A in all DPRs; this increase in ME_{215} is due to settling of the protein aggregates in the light path.

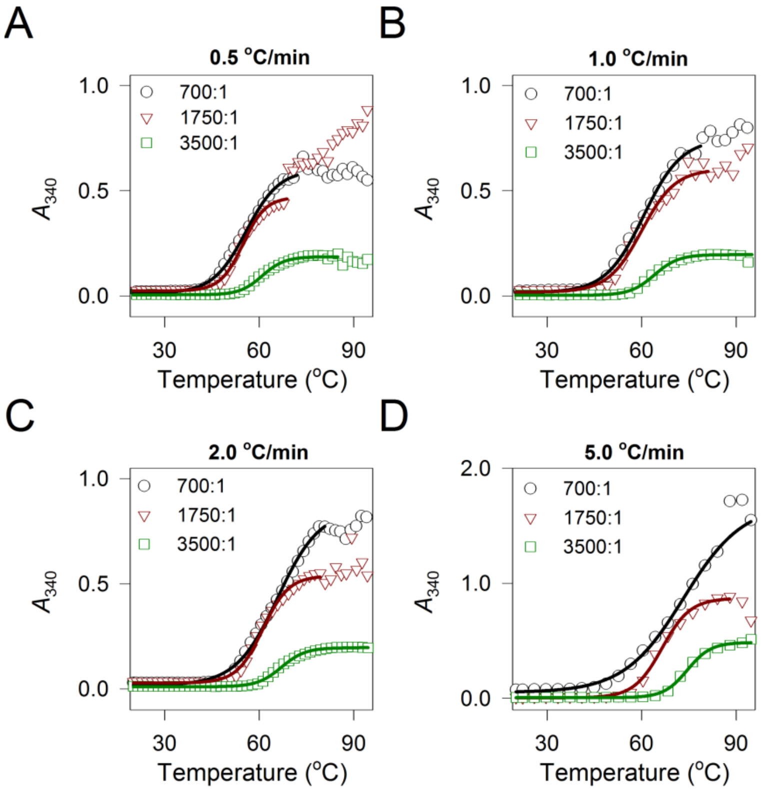

Figure S14. Ail exhibits two-state transition for oligomerization and aggregation: Inference from thermal denaturation using scattering measurements. Representative thermal denaturation profiles of Ail–WT, at varying temperature ramp rates of (A) 0.5 °C/min, (B) 1.0 °C/min, (C) 2.0 °C/min, and (D) 5.0 °C/min, monitored using absorbance at 340 nm (A_{340}). Plotted here are the blank corrected profiles of change in A_{340} with increasing temperature, across the three DPRs (700:1, 1750:1, and 3500:1). The data is fitted to a sigmoidal function to derive the T_m (see Fig. S16 for the T_m values), and the fits are shown in the respective colors as solid lines. The extent of oligomerization and aggregation decreases upon increasing the DPR (shown by increasing T_m and decrease in absolute A_{340}) at all the temperature ramp rates.

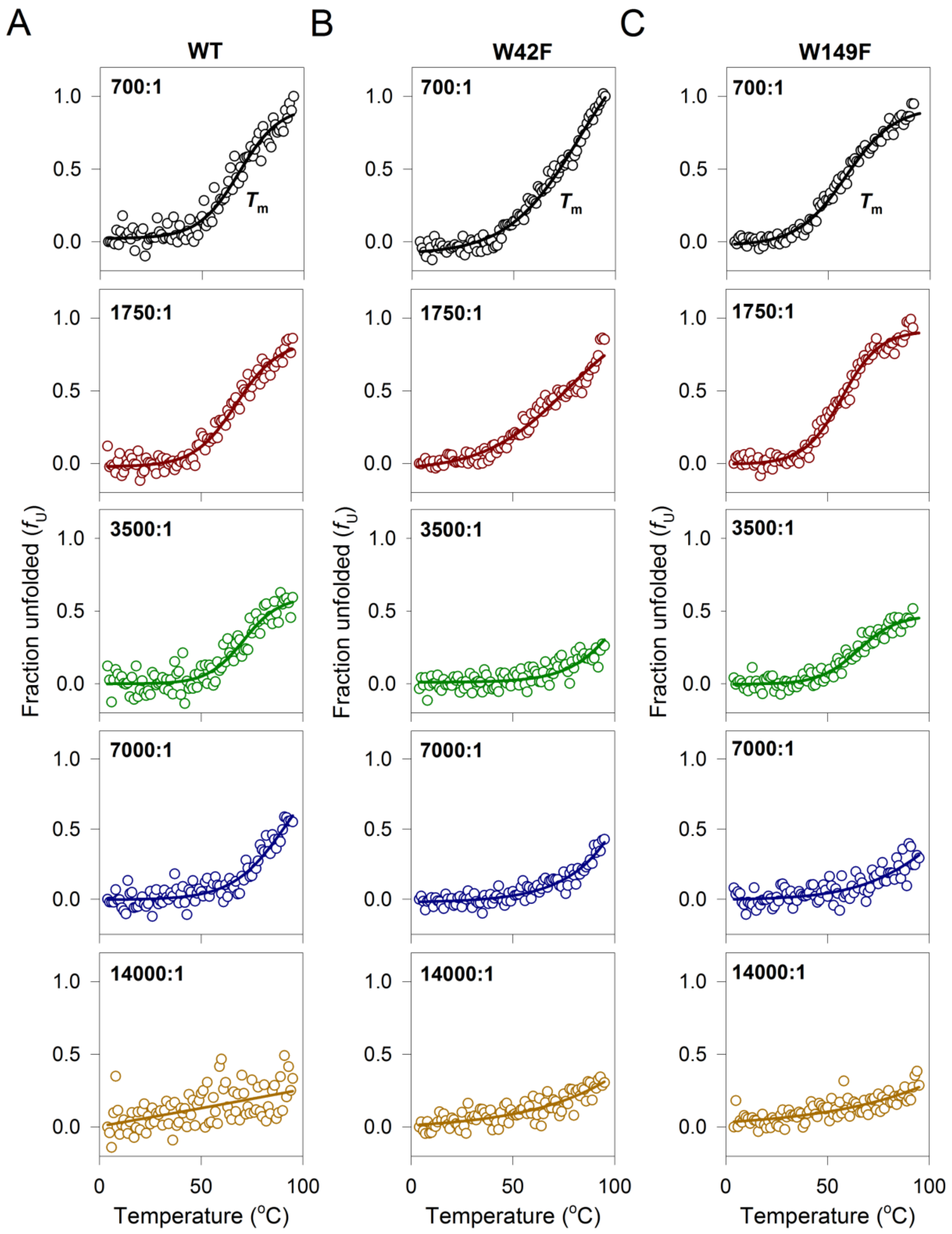

Figure S15. Ail shows DPR–dependent thermal stability: Inference from thermal denaturation measurements using far-UV CD. Representative thermal denaturation profiles of (A) Ail–WT, (B) W42F, and (C) W149F in various DPRs (700:1 – 14000:1) of LDAO. Unfolding was monitored using far-UV CD. The ME_{215} value of the respective proteins at 95 °C at the DPR of 700:1 was used to represent the completely unfolded protein, and f_U values was calculated in all cases. The extent of protein denaturation decreases as we increase the DPR, and we observe a DPR–dependent thermal stabilization of all the proteins. At moderate DPRs, Ail is optimally stable, and also shows a sigmoidal unfolding profile; this DPR of 1750:1 is therefore suitable for thermal denaturation studies (see Fig. 17 for details of the global analysis).

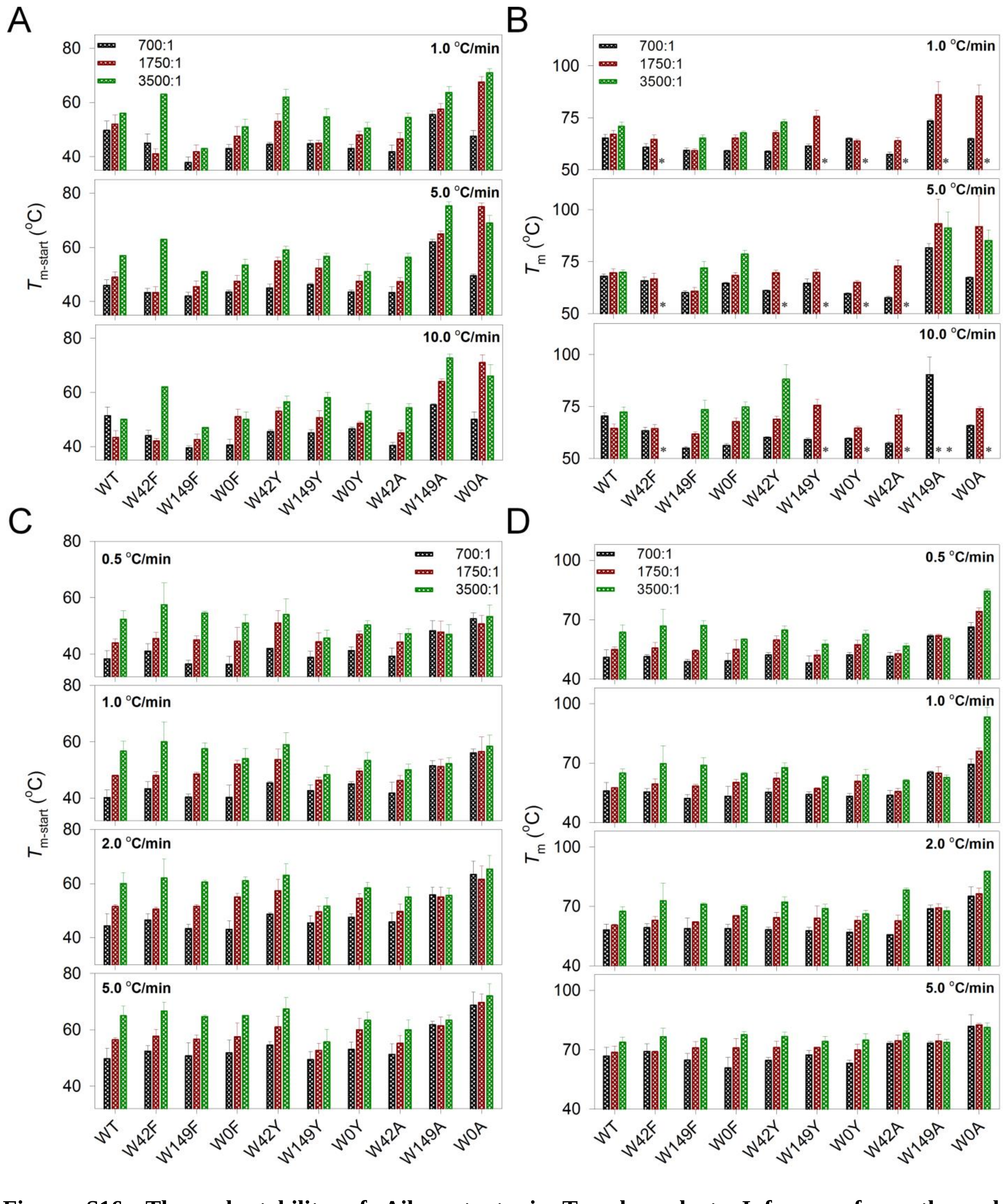

Figure S16. Thermal stability of Ail mutants is Trp–dependent: Inference from thermal denaturation study monitored using far-UV CD and scattering measurements. Comparison of thermal parameters $T_{\text{m-start}}$ (A and C) and T_m (B and D) calculated by monitoring ME_{215} (A and B) and A_{340} (C and D). The thermal parameters were calculated at various DPRs (700:1, black; 1750:1, red; and 3500:1, green) and temperature ramp rates (top to bottom panels). In all plots, errors are s. d. derived from a minimum of 2–3 independent experiments; representative data are shown only for W42F and W149F in the DPR of 3500:1, and only in the ME_{215} plots. Conditions where thermal parameters could not be

derived for some proteins at the highest DPR of 3500:1 are marked with (*). In all measurements, we observe that Trp substitution affects the thermal stability of Ail. The effect on the thermal stability also depends on the physico-chemical nature of the substituted amino acid at 42nd and 149th position (note the diverse stability of all the proteins). Furthermore, we observe that the thermal stability of Ail Trp mutants is also affected by the change in DPR and temperature ramp rates.

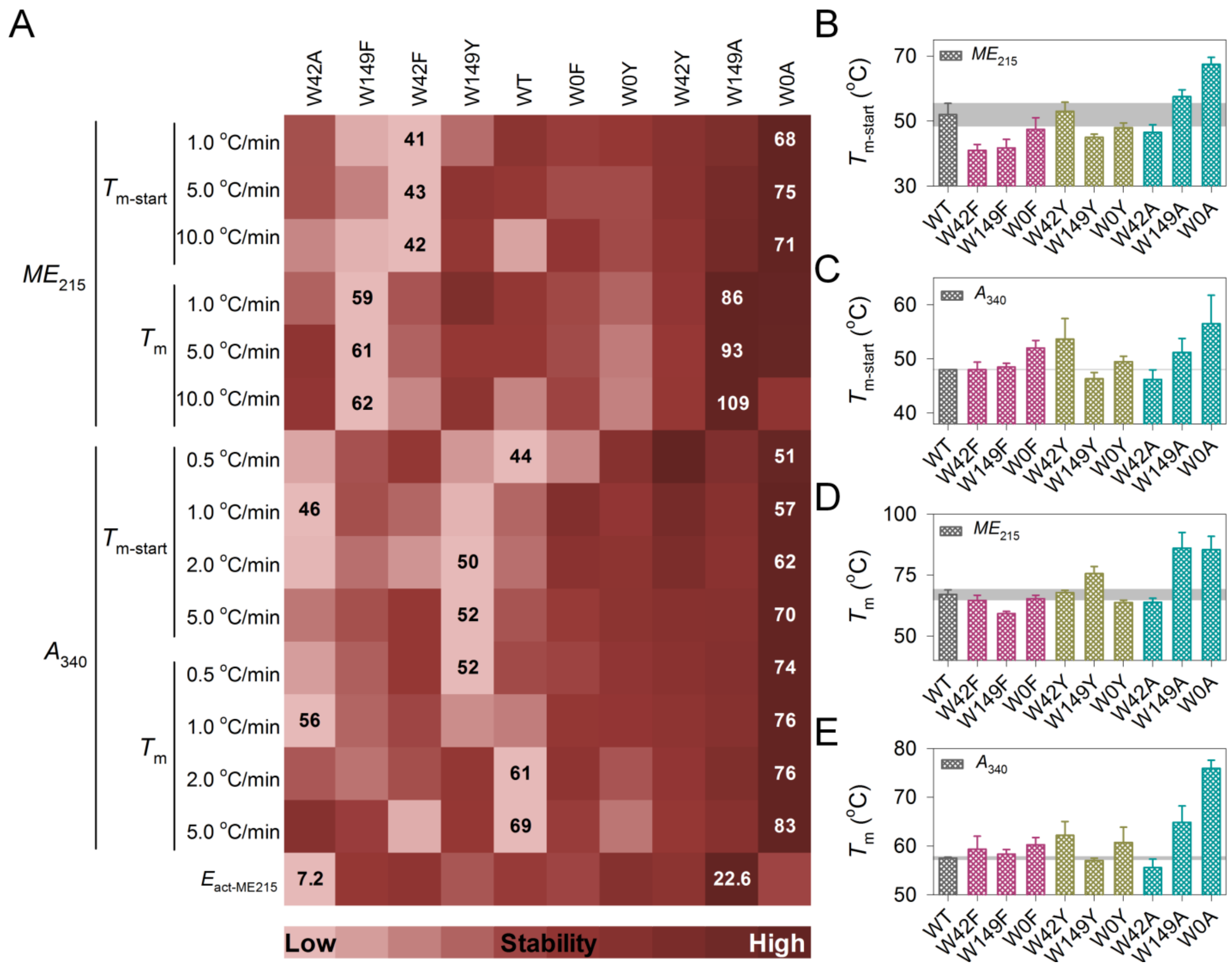

Figure S17. Trp substitution affects the thermal stability of Ail: Global comparison of thermal parameters derived using *ME*$_{215}$ and *A*$_{340}$ measurements. (A) Global comparison of thermal parameters ($T_{m\text{-start-ME}}$, $T_{m\text{-start-A340}}$, $T_{m\text{-ME}}$, $T_{m\text{-A340}}$, and $E_{act\text{-ME215}}$) at the moderate DPR of 1750:1, across Ail–WT and all the Trp mutants. The proteins are ordered from lowest stability to highest stability based on the global analysis carried out using data obtained at DPR = 1750:1 and a constant ramp rate of 1 °C/min (see section on global analysis for more details). Numbers within the global comparison plot indicate the lowest and highest mean value measured for each parameter (errors are not included). Low protein stability is indicated using a light shade of maroon, and high stability is indicated using the dark shade. Comparison of (B) $T_{m\text{-start-ME}}$, (C) $T_{m\text{-start-A340}}$, (D) $T_{m\text{-ME}}$, and (E) $T_{m\text{-A340}}$ for various Ail Trp mutants at DPR = 1750:1 and ramp rate of 1 °C/min. A simplified color pattern is used here: WT (gray), Phe mutants (pink), Tyr mutants (yellow), Ala mutants (cyan). In all plots, errors are s. d. derived from a minimum of 2–3 independent experiments. The shaded area in all plots represents the s. d. obtained for Ail–WT, and is presented to highlight those mutants having WT-like and significantly different value for the thermal parameters. Interestingly, Ail–WT shows moderate thermal stability, and Trp substitution affects the stability of Ail. Particularly, Trp→Ala substitution stabilizes the barrel (high stability of W149A and W0A proteins), whereas mutations incorporating Phe destabilizes the barrel (low stability of W42F and W149F proteins). Mutations of Trp→Tyr show moderate WT-like stability of the barrel. Overall, our results from thermal denaturation suggest that (i) aromatic residues are not required for Ail stability, and (ii) Ail stability is regulated by the chemical nature of the amino acid present at the 42nd and 149th positions. Furthermore, the varying stability we observe for W0F/Y/A when compared to W42X and

W149X proteins suggests that Phe/Tyr/Ala show position-specific barrel stabilization, as well as a non-additive effect on Ail stability.

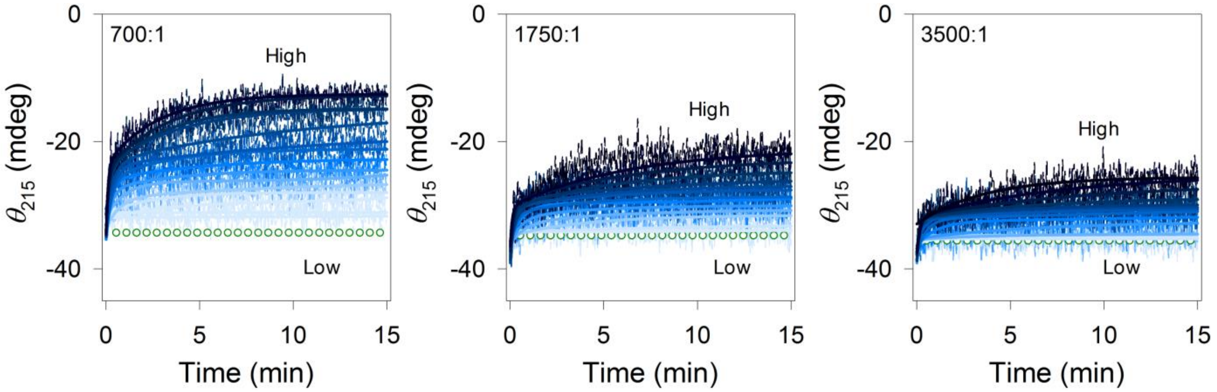

Figure S18. Aggregation kinetics of Ail–WT in various DPRs. Representative traces of Ail–WT aggregation kinetics monitored by measuring the loss in the absolute ellipticity (θ_{215}) using far-UV CD. The unfolding/ aggregation kinetics traces were monitored independently at specific holding temperatures (~40 °C – 95 °C) for 15 min, and are shown here from the low (light blue) to high (dark blue) temperature range. Each data was fitted independently to an exponential rise function (fits are shown as solid lines), to derive the rate of coupled unfolding and aggregation (k_u). The θ_{215} value for the folded protein obtained at 25 °C is shown as green symbols. Upon heating, folded Ail unfolds directly to the aggregated state. This aggregation kinetics is two-state and we find no lag phase in the kinetics traces. The rate of unfolding coupled aggregation (k_u) increases proportionately with temperature. Further, the extent of aggregation and change in θ_{215} correlates inversely as we increase the DPR (left to right panel).

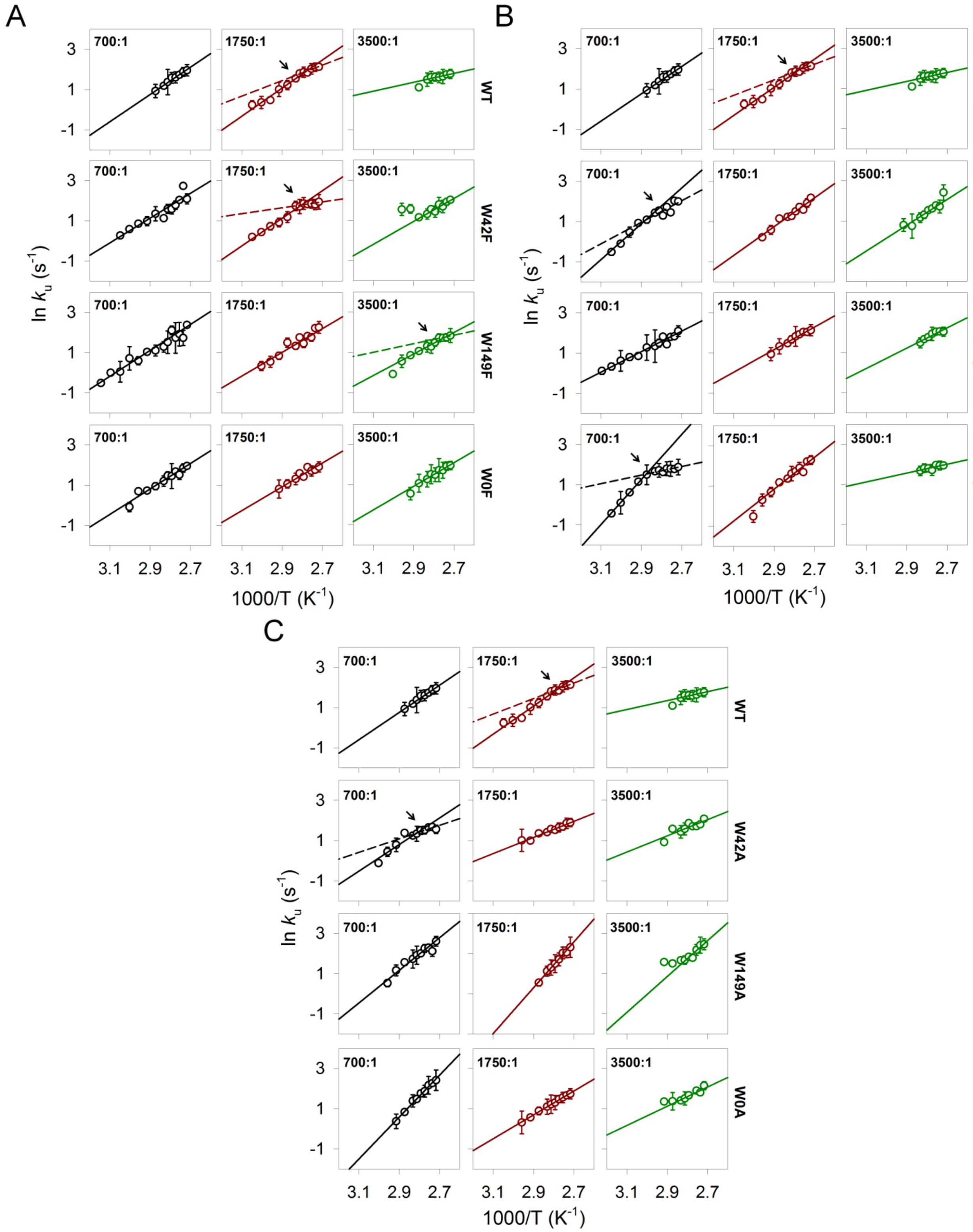

Figure S19. Arrhenius plots of various Trp mutants. Arrhenius plots of (A) Trp→Phe, (B) Trp→Tyr, and (C) Trp→Ala mutants at various DPRs (700:1, 1750:1, and 3500:1). The natural logarithm of the unfolding rate (lnk_u) shows a linear dependence on the inverse of temperature (1000/T, in units of K^{-1}). In all plots, errors are s. d. derived from a minimum of 2–3 independent experiments. The Arrhenius plot

(fits are shown as a solid line) is used to calculate E_{act} and for final analysis (5). We also observe a non-Arrhenius behavior at temperatures above 85 °C for some proteins (fits of these data are shown using dotted lines; the cross-over temperature is denoted using an arrow). Such non-Arrhenius behavior is also observed for both soluble and membrane proteins (5,8), and suggests the presence of transient intermediate(s) in unfolding and aggregation pathway of Ail. This non-Arrhenius behavior is not observed at high DPRs.

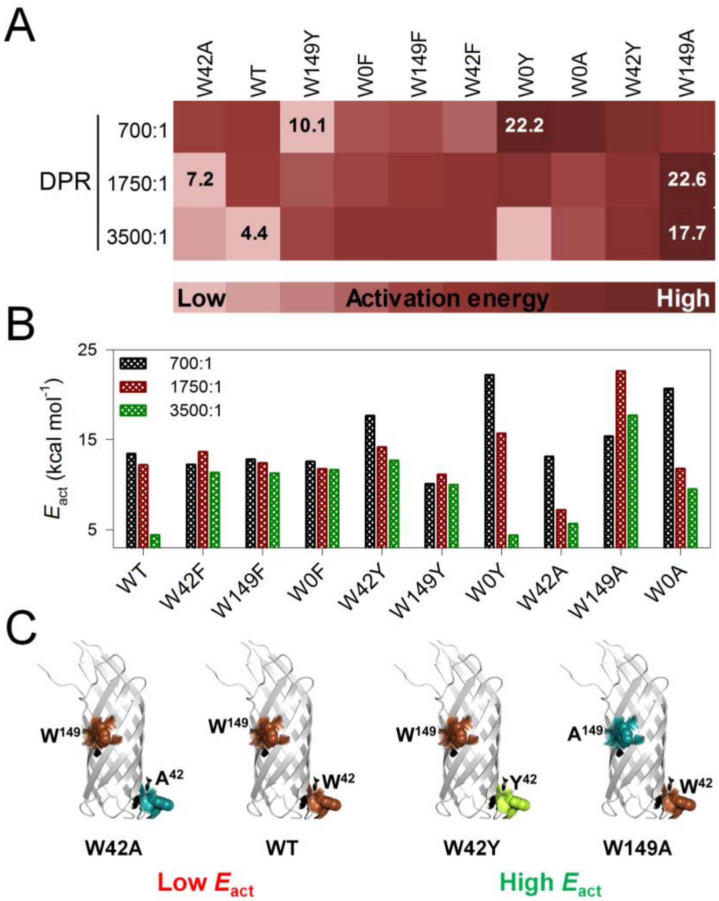

Figure S20. Trp substitution affects activation energy of Ail: Inference from isothermal unfolding measurements using far-UV CD. (A) Global comparison of E_{act} of Ail Trp mutants at all three DPRs. The proteins are arranged from lowest E_{act} (light shade) to highest E_{act} (dark shade) based on global analysis (see the section on global analysis for more details). Numbers within the global comparison plot indicate the lowest and highest value of activation energy (errors are not included for clarity). (B) Comparison of E_{act} of different Ail Trp mutants at various DPRs (700:1, black; 1750:1, red; and 3500:1, green). The E_{act} was derived by fitting the average data of Arrhenius plots. Similar to stability, the higher E_{act} observed for W149A, W42Y, and W0A, as well as the low E_{act} of W42A and W149Y, suggests that the thermal stability of Ail is dependent on the activation energy barrier for unfolding and aggregation. We also observe that Phe/Tyr/Ala show position-specific stabilization of the barrel. A non-additive effect of Trp substitution on E_{act} is also evident (shown by varying stability of W42X and W149X mutants as compared to W0X mutants). Notably, the activation energy decreases with an increase in DPR. (C) Cartoon representation of Ail showing the mutants with highest and lowest E_{act}. In line with stability measurements, W0A, W42Y, and W149A show highest E_{act}; the E_{act} is lowest in Ail–WT and W42A.

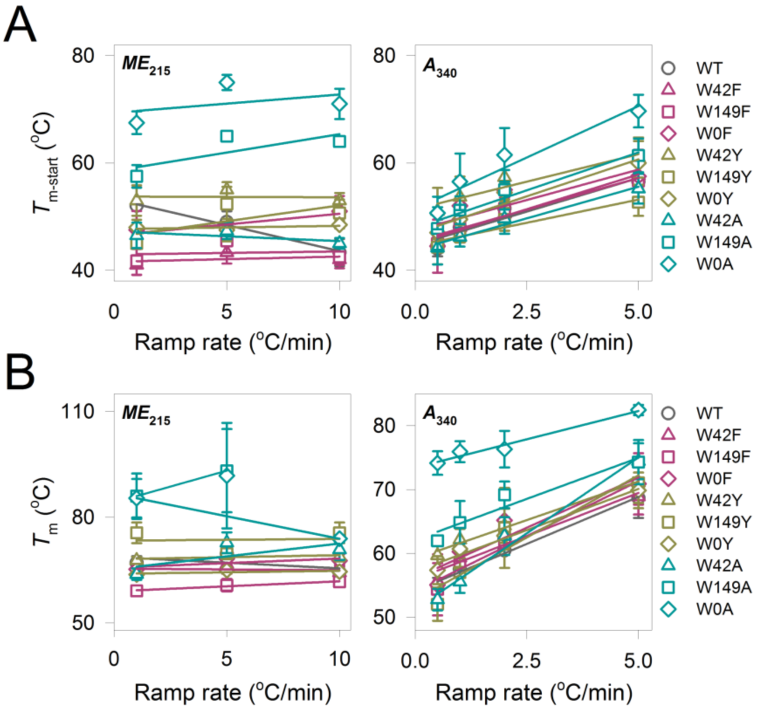

Figure S21. Ail oligomerization shows linear correlation with temperature ramp rate: Inference from thermal denaturation using far-UV CD and scattering measurements. Effect of temperature ramp rate on $T_{\text{m-start}}$ (A) and T_{m} (B), measured by monitoring ME_{215} (left panel) and A_{340} (right panel). The thermal parameters obtained at DPR = 1750:1 are compared for all the mutants. The change in thermal parameters is fitted to a polynomial equation for immediate comparison. We were not able to calculate the $T_{\text{m-ME215}}$ for W149A at the highest ramp rate of 10 °C/min due to the absence of a post-transition baseline (due to high thermal stability and resistance to unfolding and aggregation). Secondary structure loss and Ail unfolding occur independent of the temperature ramp rate (A and B; left panel), but Ail oligomerization and aggregation show a clear correlation with temperature ramp rate (A and B; right panel). The latter conclusion is supported by the linear increase in the start and mid-point temperature of oligomerization with increasing the temperature ramp rate. A simple color scheme is used here: WT (gray), Phe mutants (pink), Tyr mutants (yellow), Ala mutants (cyan). In all plots, errors are s. d. derived from a minimum of 2–3 independent experiments.

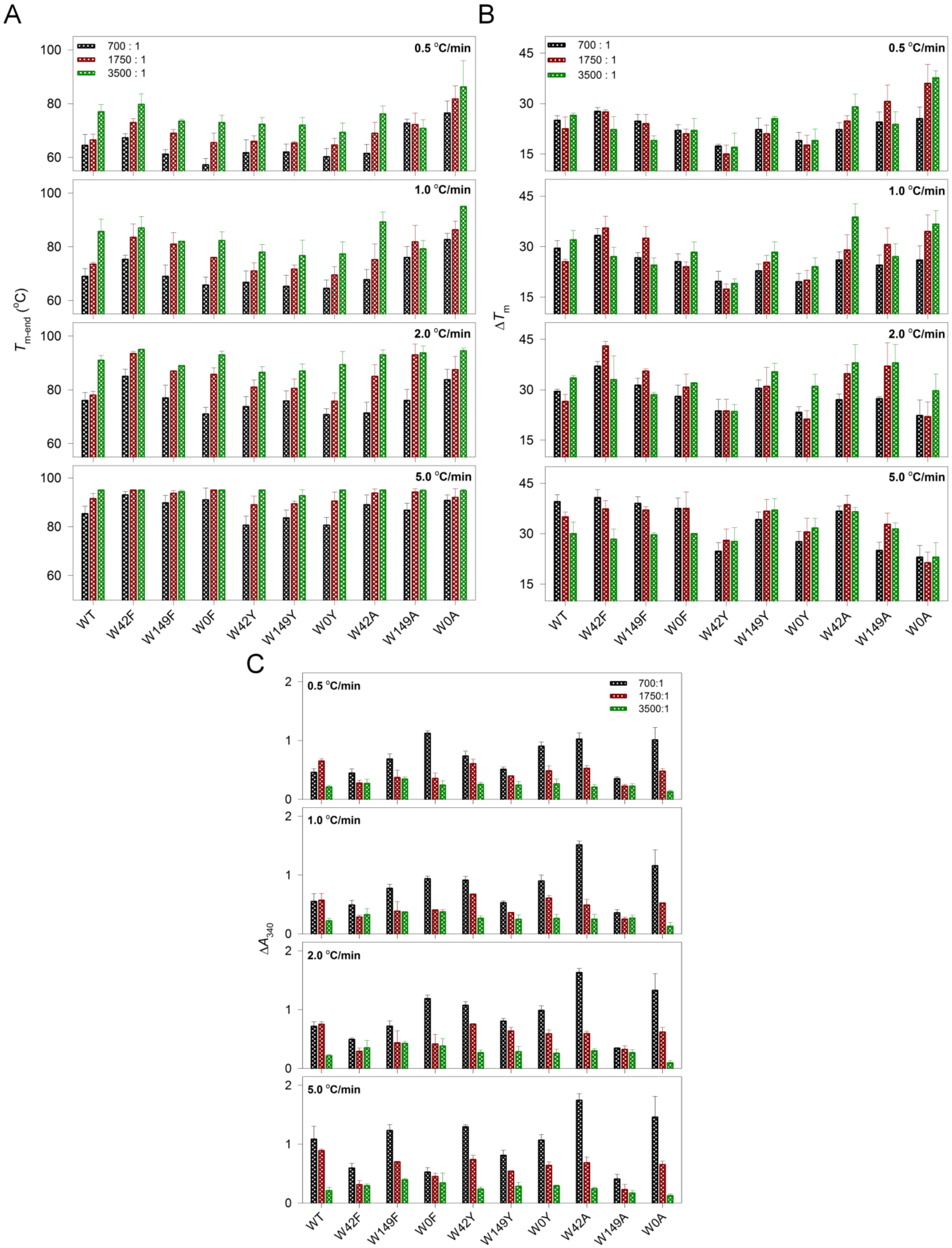

Figure S22. Trp substitution affects aggregation propensity of Ail: Inference from scattering measurements. Comparison of (A) $T_{\text{m-end}}$, (B) ΔT_m, and (C) ΔA_{340} calculated from A_{340} measurements across the Trp mutants. The thermal parameters were calculated at various DPRs (700:1, black; 1750:1,

red; and 3500:1, green) and temperature ramp rates (top to bottom panels). In all plots, errors are s. d. derived from a minimum of 2 – 3 independent experiments. Trp substitution affects aggregation propensity of Ail; this property also depends on the chemical nature of the substituted amino acid at the 42nd and 149th position. Here, the proteins with high stability (Trp→Ala mutants) also resist aggregation (i.e., show the least aggregation propensity). We observe a weak correlation between thermal stability and aggregation propensity of Ail. The highly destabilized Trp→Phe mutants (W42F and W149F) also show reduced aggregation propensity. Interestingly, the moderately stabilized Trp→Tyr mutants (W42Y and W0Y) show the highest aggregation propensity. Furthermore, we also observe that the aggregation propensity of Ail Trp mutants is also affected by the change in DPR and temperature ramp rates.

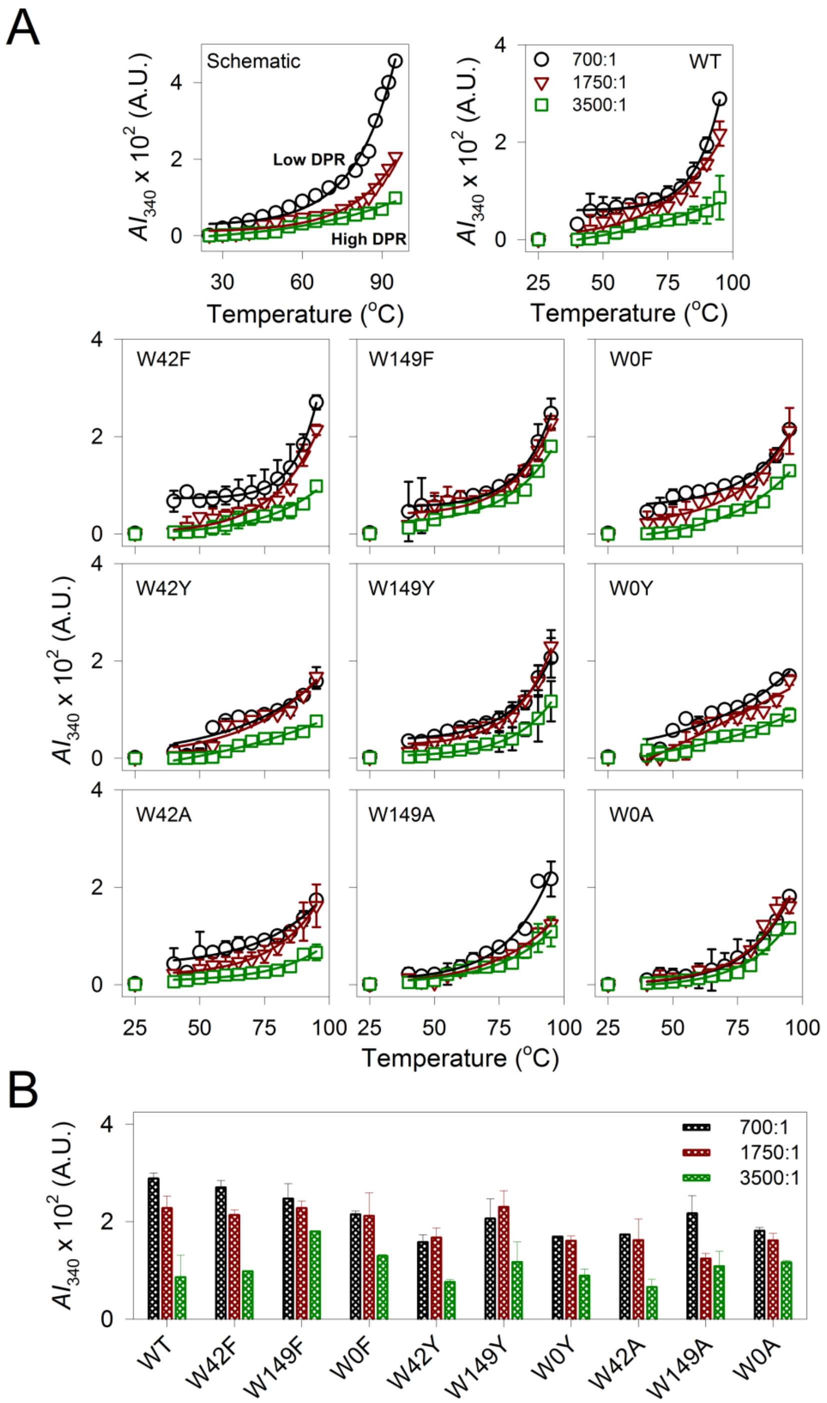

Figure S23. Aggregation index measurements for Ail Trp mutants. (A) Change in aggregation index (AI_{340}) at various DPRs (700:1, black; 1750:1, red; and 3500:1, green) with increasing temperature. The folded samples were heated at various temperatures (40 – 95 °C), and AI_{340} was calculated after cooling the samples to 25 °C. AI_{340} shows an exponential rise with increasing temperature (fits are shown as solid lines). Note that all the folded protein exhibits an aggregation index (AI_{340}) and $A_{320} \approx 0$. (B) Comparison of AI_{340} calculated at 95 °C for various Ail Trp mutants at all three DPRs. In all plots, errors are s. d. derived from a minimum of 2–3 independent experiments. The AI_{340} proportionally decreases as we increase the DPR. Interestingly, we observe that AI_{340} is strongly regulated by the amino acid present at the 42nd and 149th position. Further, the AI_{340} proportionally decreases with increase in DPR.

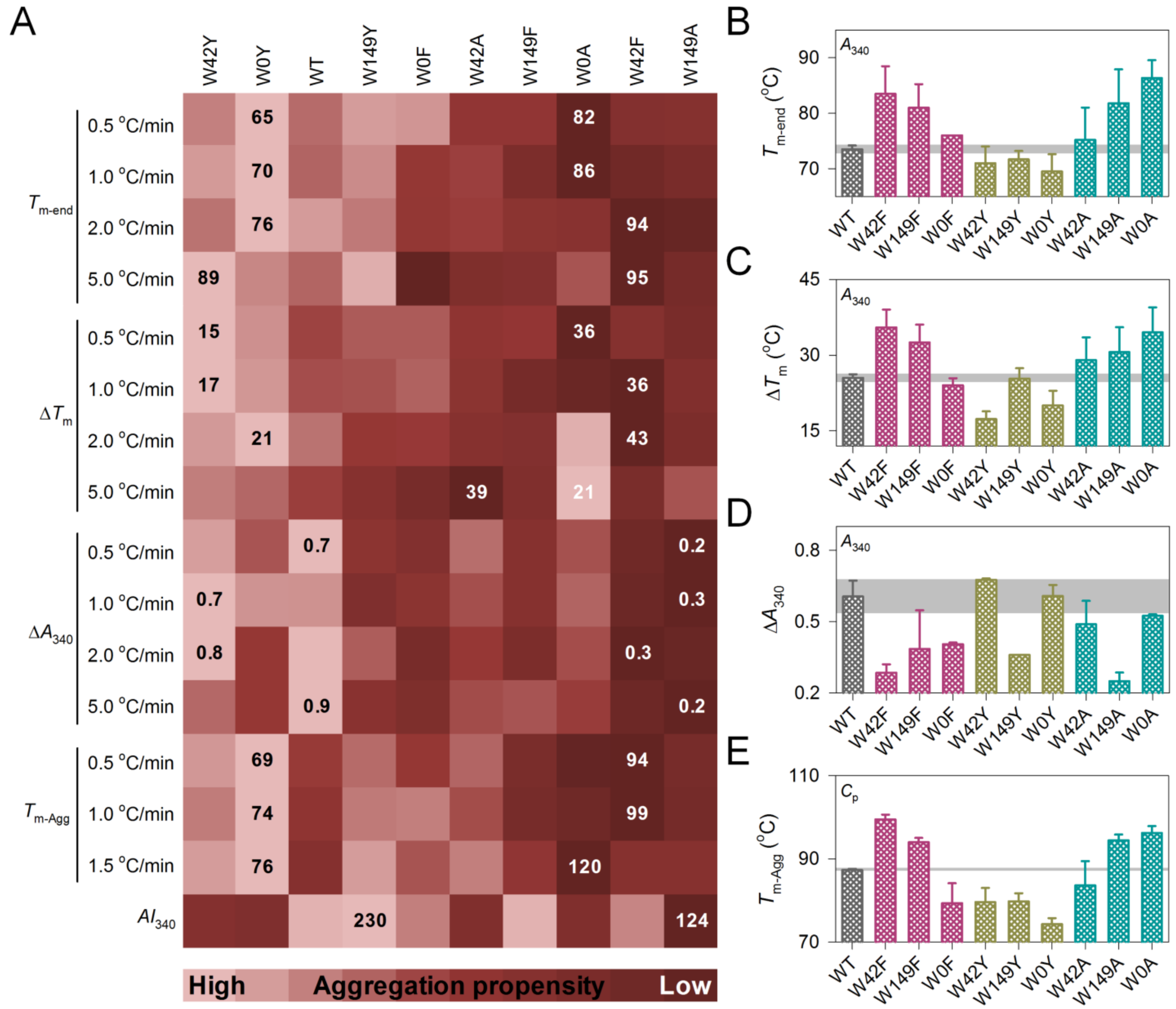

Figure S24. Trp→Tyr substitution promotes aggregation propensity of Ail: Inference from thermal denaturation study using scattering and DSC measurements. (A) Global comparison of various thermal parameters ($T_{\text{m-end}}$, ΔT_{m}, ΔA_{340}, $T_{\text{m-Agg}}$, and AI_{340}) as a measure of the aggregation propensity of Ail Trp mutants. Here, the data are sorted according to the decreasing order of aggregation propensity, and is based on the global analysis carried out at a constant DPR of 1750:1 and ramp rate of 1 °C/min (see the section on global analysis for details). Numbers within the global comparison plot indicate the lowest and highest value of each parameter (errors are not included for clarity, and are provided instead in plots B-E) (light maroon: mutants with high aggregation propensity; dark maroon: mutants with low aggregation propensity). Comparison of (B) $T_{\text{m-end-A340}}$, (C) $\Delta T_{\text{m-A340}}$, (D) ΔA_{340}, and (E) $T_{\text{m-Agg}}$ for various Ail Trp mutants at a constant DPR of 1750:1 and temperature ramp rate of 1 °C/min. A simplified color pattern is used here: WT (gray), Phe mutants (pink), Tyr mutants (yellow), Ala mutants (cyan). In all plots, errors are s. d. derived from a minimum of 2–3 independent experiments. The shaded area in all plots represents the s. d. obtained for Ail–WT. The data supports our conclusion that Trp substitution affects the aggregation propensity of Ail. Here, Trp→Phe substitution is tolerated, but Trp→Tyr substitution increases the aggregation propensity and is deleterious for the barrel.

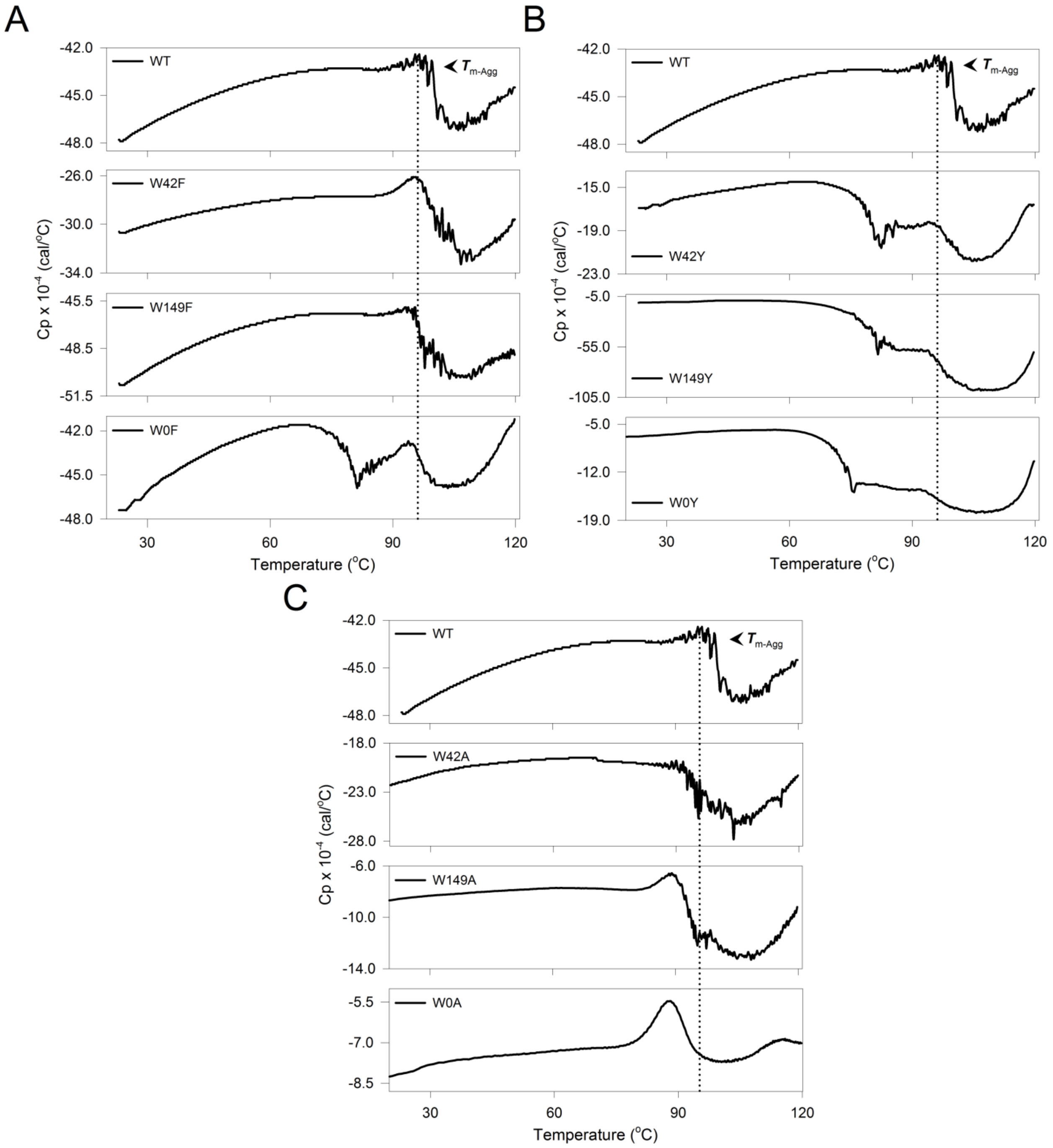

Figure S25. Thermal denaturation profiles of Ail Trp mutants monitored using differential scanning microcalorimetry. Representative DSC thermal denaturation profiles of (A) Trp→Phe, (B) Trp→Tyr, and (C) Trp→Ala mutants, at the DPR of 1750:1 and temperature ramp rate of 1 °C/min. The buffer-corrected specific (molar) heat capacity values (C_p) are plotted against increasing temperature. Data for Ail–WT are shown for comparison in the top panel of (A – C), and $T_{m\text{-}Agg}$ is denoted by an arrow. We do not observe a distinct unfolding transition for most of the proteins at the DPRs of 700:1 and 1750:1; instead we observe a single exothermic transition representing protein aggregation. Interestingly, the aggregation transition varies across the proteins (dotted line represents the unfolding transition for WT). We observe unfolding only for W42F, W149A, and W0A mutants at moderated DPR, whereas all

other proteins directly aggregate upon thermal denaturation. The $T_{m\text{-}Agg}$ for all the protein is compared in Fig. S27.

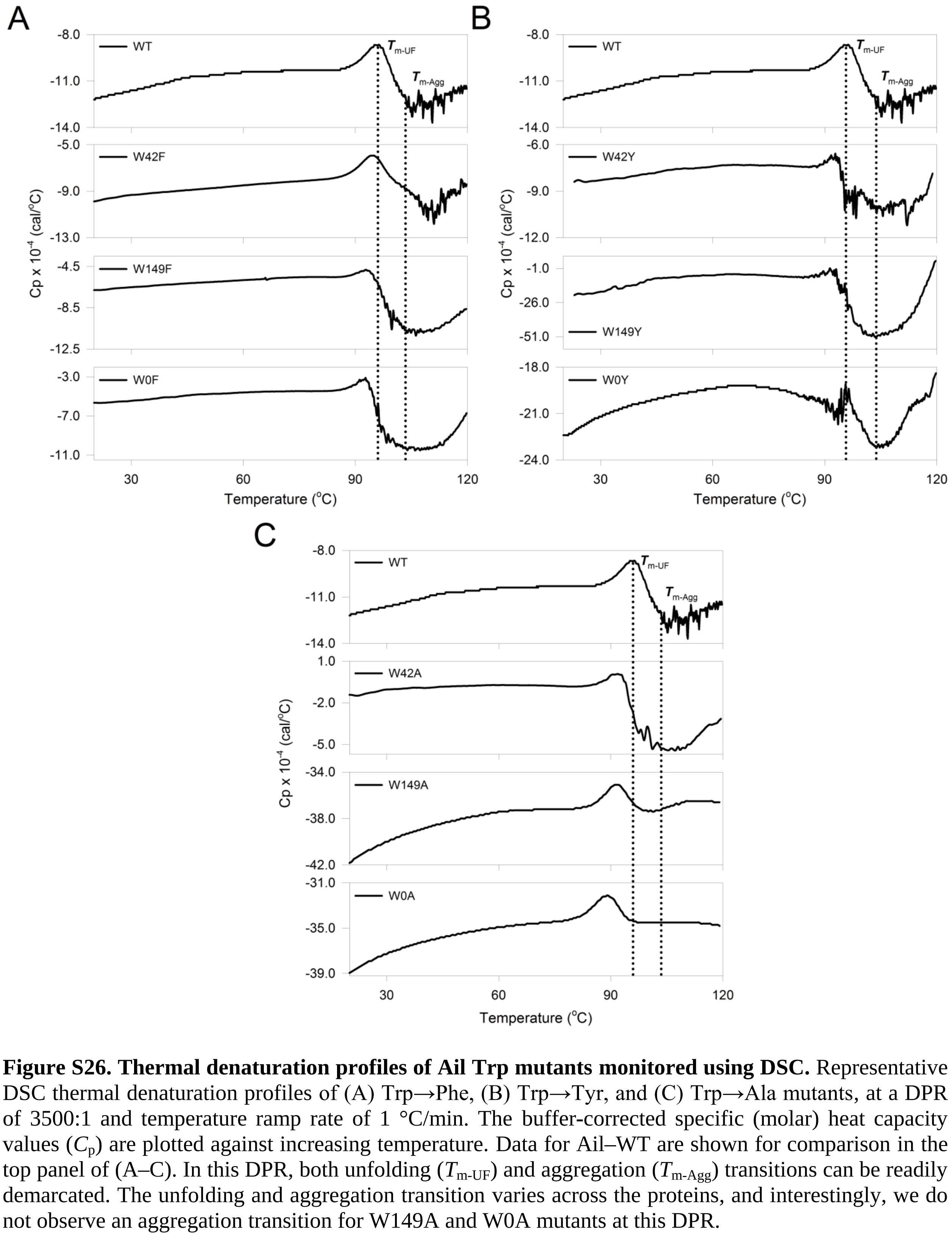

Figure S26. Thermal denaturation profiles of Ail Trp mutants monitored using DSC. Representative DSC thermal denaturation profiles of (A) Trp→Phe, (B) Trp→Tyr, and (C) Trp→Ala mutants, at a DPR of 3500:1 and temperature ramp rate of 1 °C/min. The buffer-corrected specific (molar) heat capacity values (C_p) are plotted against increasing temperature. Data for Ail–WT are shown for comparison in the top panel of (A–C). In this DPR, both unfolding ($T_{m\text{-UF}}$) and aggregation ($T_{m\text{-Agg}}$) transitions can be readily demarcated. The unfolding and aggregation transition varies across the proteins, and interestingly, we do not observe an aggregation transition for W149A and W0A mutants at this DPR.

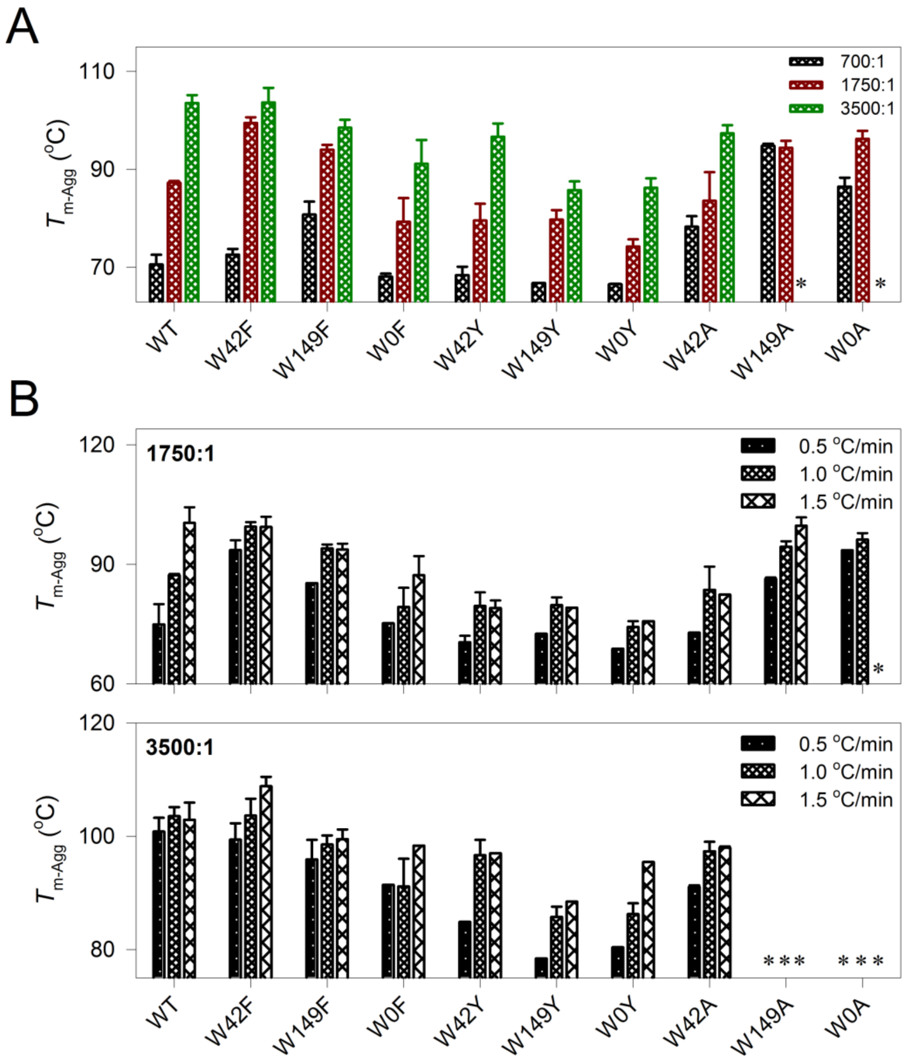

Figure S27. DSC measurements confirm varied aggregation temperatures for the Ail Trp mutants. (A) Comparison of $T_{\text{m-Agg}}$ derived from DSC measurements at various DPRs (700:1, black; 1750:1, red; and 3500:1, green) and at a constant temperature ramp rate of 1 °C/min. Ail shows a DPR-dependent stabilization and a proportional increase in $T_{\text{m-Agg}}$ upon increasing the DPR. (B) Effect of temperature ramp rates on $T_{\text{m-Agg}}$ of Ail Trp mutants at a DPR of 1750:1 (top panel) and 3500:1 (bottom panel). We observe an increase in $T_{\text{m-Agg}}$ upon increasing the temperature ramp rate, which confirms the contribution of kinetic factors in Ail stability and aggregation. Conditions where the protein did not aggregate, and $T_{\text{m-Agg}}$ could not be measured have been indicated with (*). In all plots, errors are s. d. derived from a minimum of 2–3 independent experiments. Overall, the results show that aggregation is highest in Ail upon Trp→Tyr substitution, in all the DPRs and temperature ramp rates.

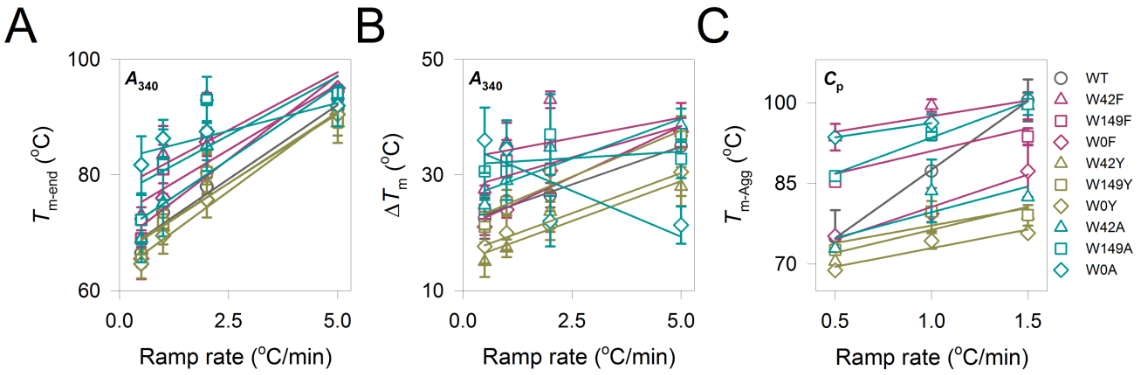

Figure S28. Ail oligomerization and aggregation increases linearly with temperature ramp rate: Inference from thermal denaturation using scattering and DSC measurements. Effect of temperature ramp rate on (A) $T_{\text{m-end}}$, (B) ΔT_{m}, and (C) $\Delta T_{\text{m-Agg}}$ measured by ME_{215} and C_{p} for various Ail Trp mutants. For simplicity, the comparison is shown at the DPR of 1750:1. Ail aggregation correlates inversely with an increase in the ramp rate for all the proteins (fits are shown as solid lines). Here, all the proteins show a proportional decrease in aggregation propensity upon increasing the temperature ramp rate. A simplified color scheme is used here: WT (gray), Phe mutants (pink), Tyr mutants (yellow), Ala mutants (cyan). In all plots, errors are s. d. derived from a minimum of 2–3 independent experiments.

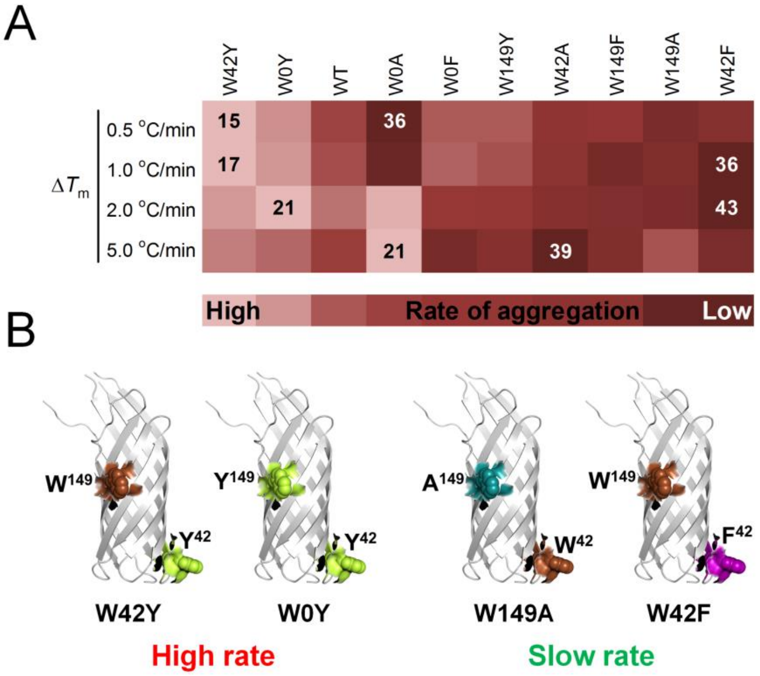

Figure S29. Trp→Tyr mutants show the highest rate and cooperativity of aggregation: Inference from thermal denaturation measurements using scattering. (A) Global comparison of cooperativity and rate of aggregation (ΔT_m) at the moderate DPR of 1750:1 and various temperature ramp rates. The mutants are arranged from a high rate of aggregation (light shade) to a low rate of aggregation (dark shade). Numbers within the global comparison plot show the data range measured for each parameter (errors are not included). The Trp→Phe/Ala mutants show the least rate of aggregation, whereas the Trp→Tyr mutants show highly cooperative aggregation. (B) Cartoon representation of Ail highlighting the mutants with the highest and lowest rate of aggregation. The colors used are: Trp, brown; Phe, pink; Tyr, yellow; Ala, cyan.

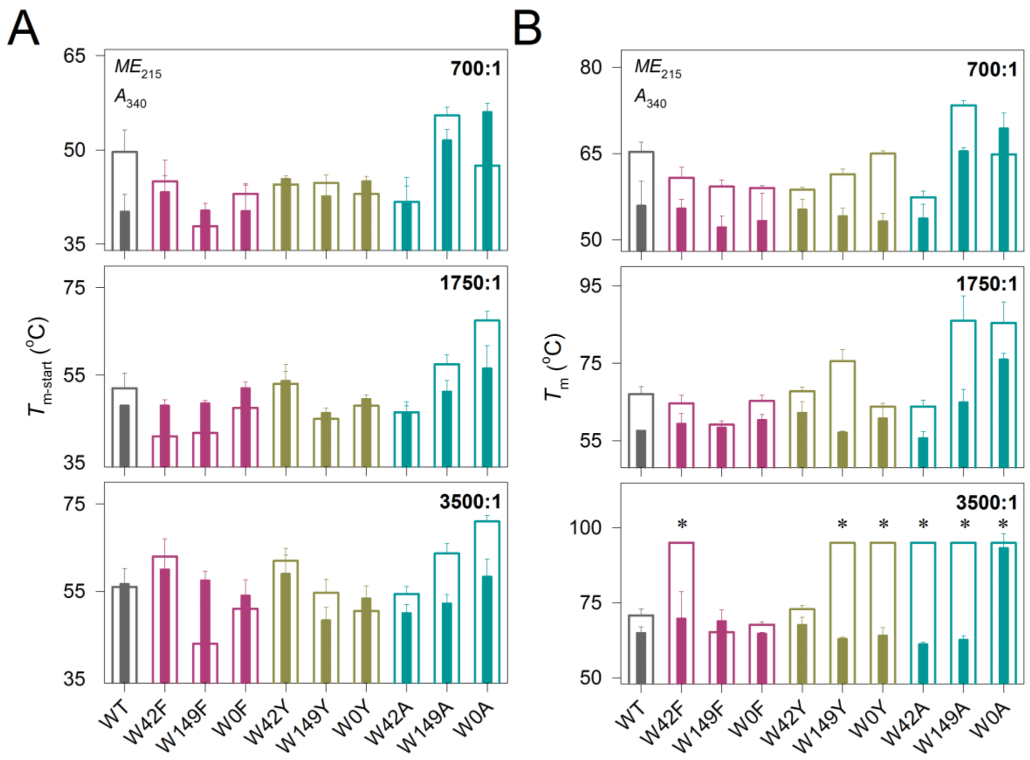

Figure S30. Ail aggregation is achieved by the formation of oligomeric assemblies: Inference from thermal denaturation analysis using far-UV CD and scattering measurements. (A) Histogram comparing $T_{\text{m-start-ME215}}$ and $T_{\text{m-start-A340}}$, to deduce the mechanism of Ail aggregation at all three DPRs (700:1, top panel; 1750:1, middle panel; and 3500:1, bottom panel), at a constant temperature ramp rate of 1 °C/min. If $T_{\text{m-start-A340}} < T_{\text{m-start-ME215}}$, then initiation of oligomerization occurs before barrel unfolding starts, whereas a $T_{\text{m-start-A340}} > T_{\text{m-start-ME215}}$ indicates that unfolding precedes oligomerization (Fig. 5A). In Ail, nearly all the proteins show a $T_{\text{m-start-A340}} < T_{\text{m-start-ME215}}$, indicating that Ail oligomerization occurs before complete loss in protein structure. (B) Histogram comparing $T_{\text{m-ME215}}$ and $T_{\text{m-A340}}$, to deduce the mechanism of Ail aggregation at all three DPRs (top to bottom), at a constant temperature ramp rate of 1 °C/min. Here again, we observe that $T_{\text{m-A340}}$ is less then $T_{\text{m-ME215}}$, supporting our conclusion that Ail oligomerization and aggregation occurs before complete unfolding or loss in secondary structure. A simplified color pattern is used here: WT (gray), Trp→Phe mutants (pink), Trp→Tyr mutants (yellow), Trp→Ala mutants (cyan). Error bars represent s. d. derived from at least 3 independent experiments. Conditions, where we were not able to measure $T_{\text{m-ME215}}$ have been denoted as (*), and we have assigned $T_{\text{m-ME215}}$ = 95 °C merely for purposes of comparison. Overall, we observe that Ail aggregation is achieved by the formation of oligomeric assemblies prior to complete unfolding or loss in secondary structure.

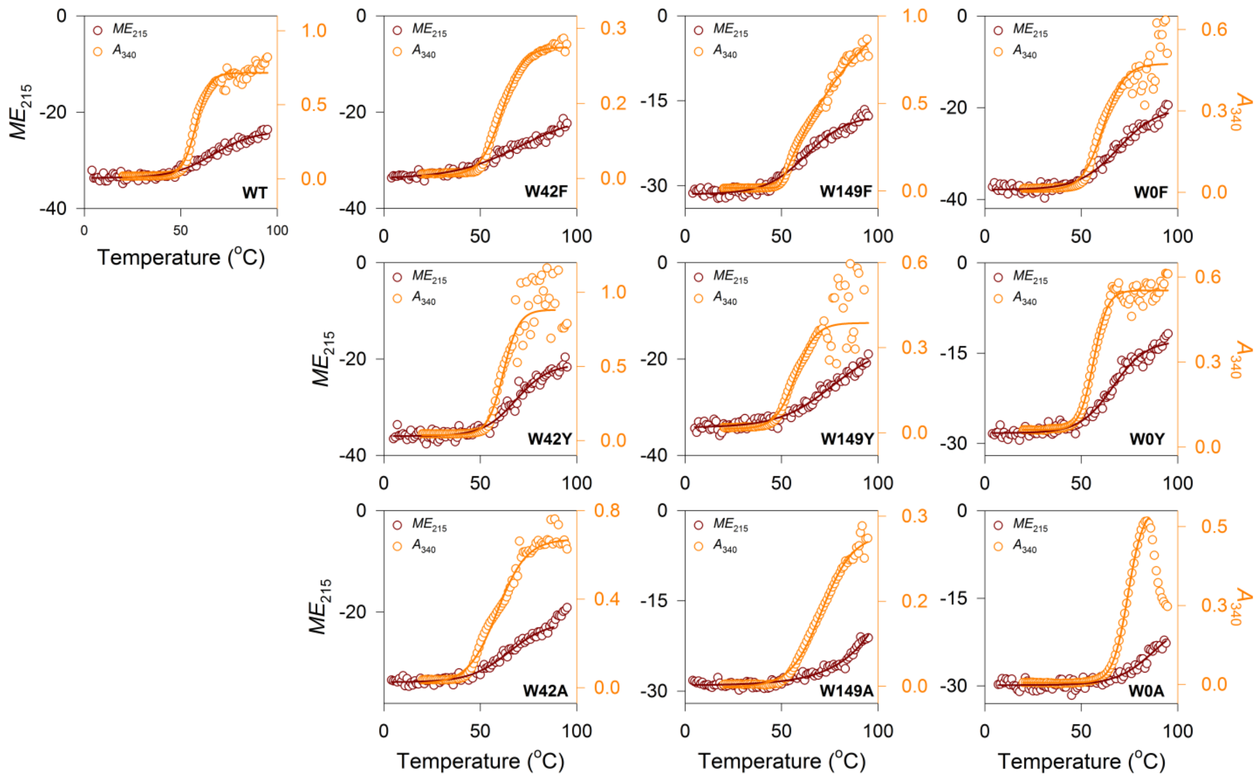

Figure S31. Ail show cooperative aggregation but slow unfolding. Representative thermal denaturation profiles of Ail measured using far-UV CD (ME_{215}) and UV-Vis spectroscopy (A_{340}). The data compares the thermal denaturation curves for the proteins folded at a constant DPR of 1750:1; all the measurements were carried out at a constant temperature ramp rate of 1 °C/min. Interestingly, all the proteins show a cooperative aggregation event (orange symbols; measured using A_{340}) but unfolding transitions that are slower (dark red symbols; measured using ME_{215}). Note that for some of the mutants (e.g., W149A and W0A), the loss in secondary structure content is nucleated only at temperatures >80 °C, and the loss in structure is incomplete at the highest recording temperature of 95 °C. The comparison reveals that Ail retains its secondary structure even at temperatures near $T_{m\text{-}A340}$. These results are consistent with our NMR measurements and confirm that Ail aggregation occurs through the association of structured oligomers.

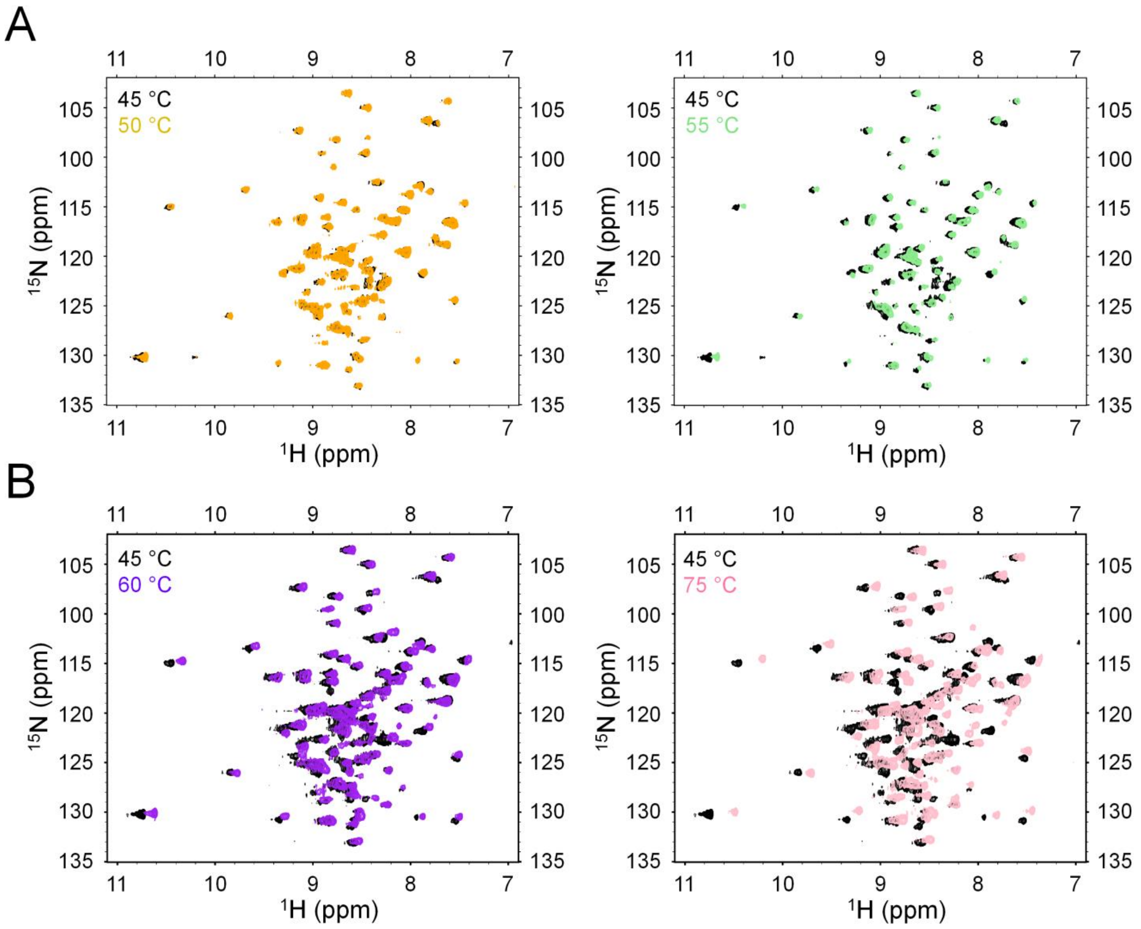

Figure S32. Ail does not unfold at higher temperatures and remains structured. Representative ^{1}H-^{15}N HSQC-TROSY spectra of folded Ail–WT (A) and W149A (B) (DPR of 1750:1), at various temperatures. Shown here is the superposition of data recorded at 45 °C (black) with data obtained at higher temperatures (indicated within each overlay for both proteins). We observed well-dispersed resonances for both the proteins, even at the temperatures near to their mid-point of thermal denaturation ($T_{m\text{-}A340}$). It is indeed plausible that the unstable unfolded state quickly aggregates and is removed from the population detected using NMR. However, NMR, being a spectroscopic technique, is sensitive to the absolute concentrations of the sample. Under identical recording conditions and processing parameters (see SI methods), a substantial reduction in the peak widths must be observed if a significant population of the sample is aggregated and causes line broadening effects. We do not observe substantial losses in S/N beyond the direct effects of temperature in our HSQC spectra. The data supports our conclusion that most of the protein is folded even at higher temperatures, and Ail aggregation occurs through the association of structured oligomers.

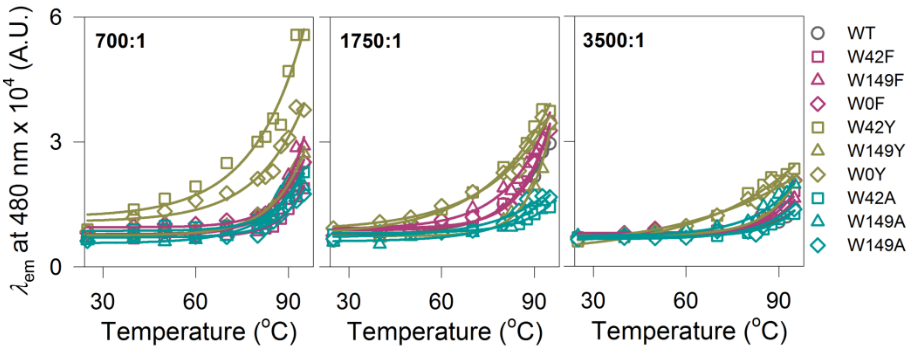

Figure S33. Ail aggregates show high binding affinity to thioflavin T dye. Ail aggregates show affinity to bind ThT dye. Here, the folded protein in different DPRs (700:1, left panel; 1750:1, middle panel; and 3500:1, right panel) was heated at specific temperatures for 10 min to prepare aggregated samples. Samples were cooled to 25 °C, and ThT dye was added to a final concentration of 10 μM. The change in ThT fluorescence at $\lambda_{em\text{-}max} \approx$ 480 nm (for aggregated samples) with increasing temperatures was then compared across the proteins. The change in ThT fluorescence increases exponentially with increasing temperature (fits are shown as solid lines), at all the DPRs. The ThT fluorescence intensity at $\lambda_{em\text{-}max} \approx$ 480 nm is influenced by Trp substitution and change in DPR. The change ThT fluorescence is also observed due to an increase or decrease in the amount of fibrillar aggregates. Overall, we find the ThT binding affinity of Ail aggregates correlates well with the aggregation propensity measurements. Here, Ail Trp→Tyr mutants show highest ThT fluorescence whereas Trp→Ala mutants show weak ThT fluorescence.

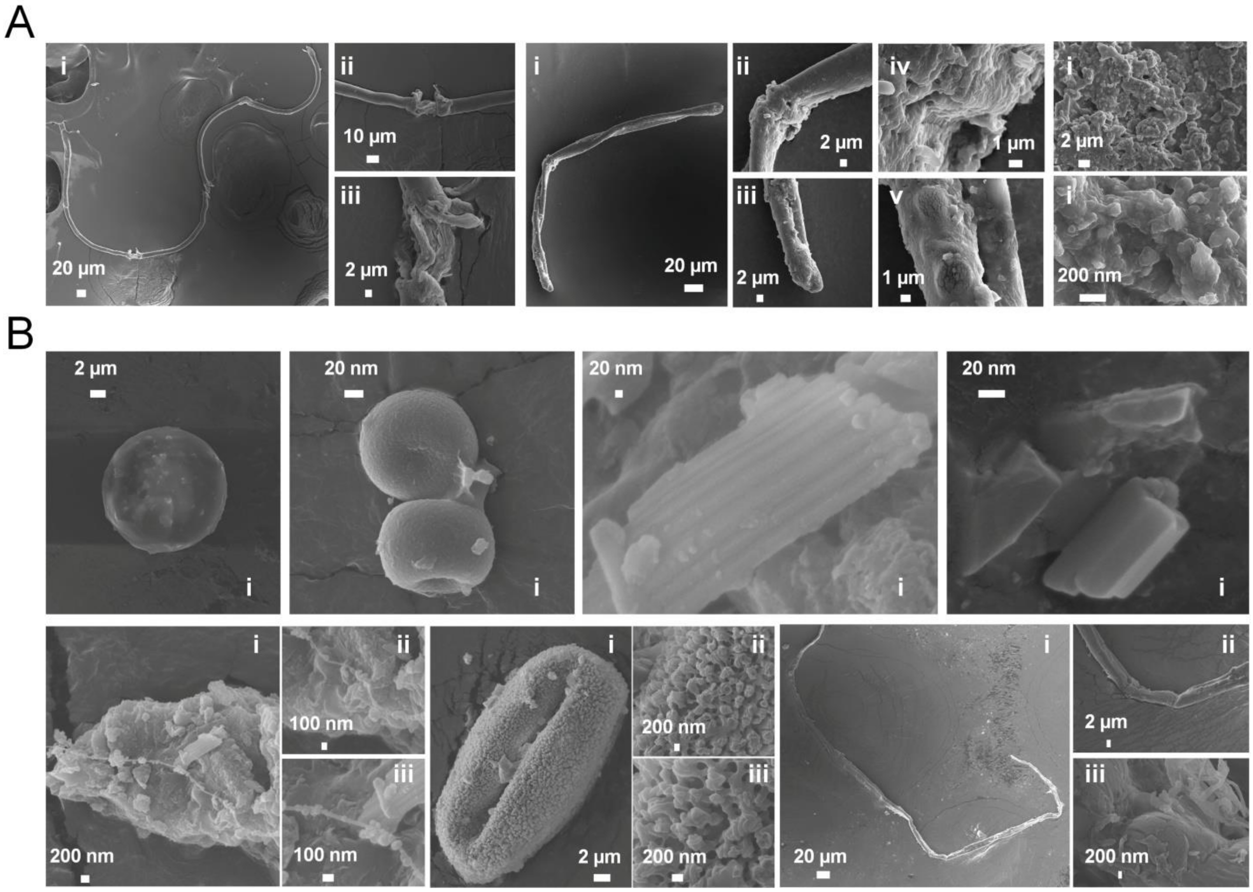

Figure S34. Scanning electron microscopy reveals dual morphologies of Ail aggregates. Representative SEM profiles of heat-denatured aggregated samples of folded Ail–WT (A) and Ail–WT resuspended in 20 mM Tris-HCl pH 8.5 (B). The aggregated sample for (A) was prepared by heating the folded sample at 95 °C for 10 min, whereas for (B), Ail powder was directly resuspended in the buffer without heating. The buffer-resuspended sample served as the control to confirm (i) that the observed morphologies are not an artifact of heating Ail protein or interference from LDAO, and (ii) whether Ail has inherent tendency to form amyloid-like β-sheet rich aggregates. The images were acquired at low magnification (panel i) and high magnification (panel ii, iii, iv, and v). Ail aggregates display both fibrillar and amorphous morphologies. Similar morphologies observed in the heat denatured folded sample and directly resuspended sample suggests that Ail has an inherent tendency to form fibrillar aggregates, and the morphologies we observe are not artifacts of heating or detergent micelles. Note how the size of the aggregates is larger by at least one magnitude in the folded sample (compare (A) and (B)). Higher order aggregates are therefore likely to form during the aggregation of folded Ail protein.

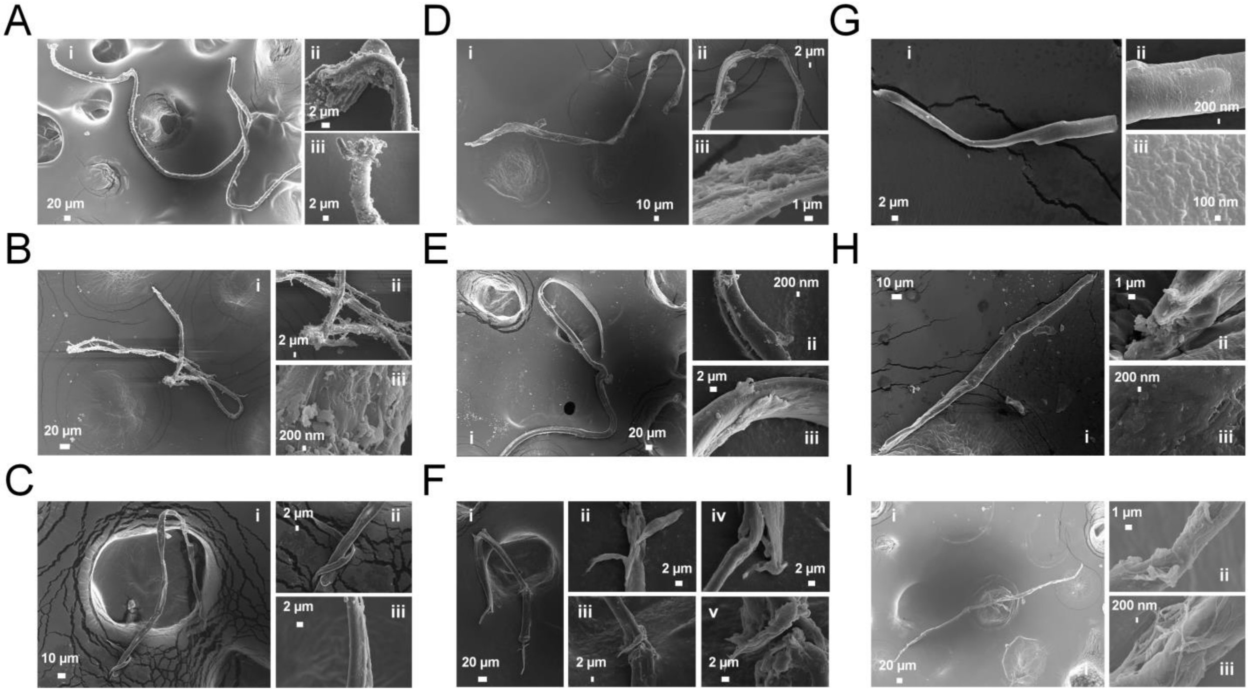

Figure S35. Ail Trp mutants show fibrillar morphology upon aggregation. (A – I) Representative SEM profiles of Ail W42F, W149F, W0F, W42Y, W149Y, W0Y, W42A, W149A, and W0A prepared by heating the folded samples at 95 °C for 10 min. The images acquired at low magnification (panel i) and high magnification (panel ii, iii, iv, and v) are shown. SEM measurements suggest that all the proteins show similar morphologies upon aggregation. Furthermore, the difference in ThT fluorescence intensity, despite the similar morphologies in SEM, directly supports our conclusion that that Trp substitution affects the extent of aggregation (i.e., total amount of aggregates obtained). This conclusion also correlates well with our results from aggregation propensity measurements.

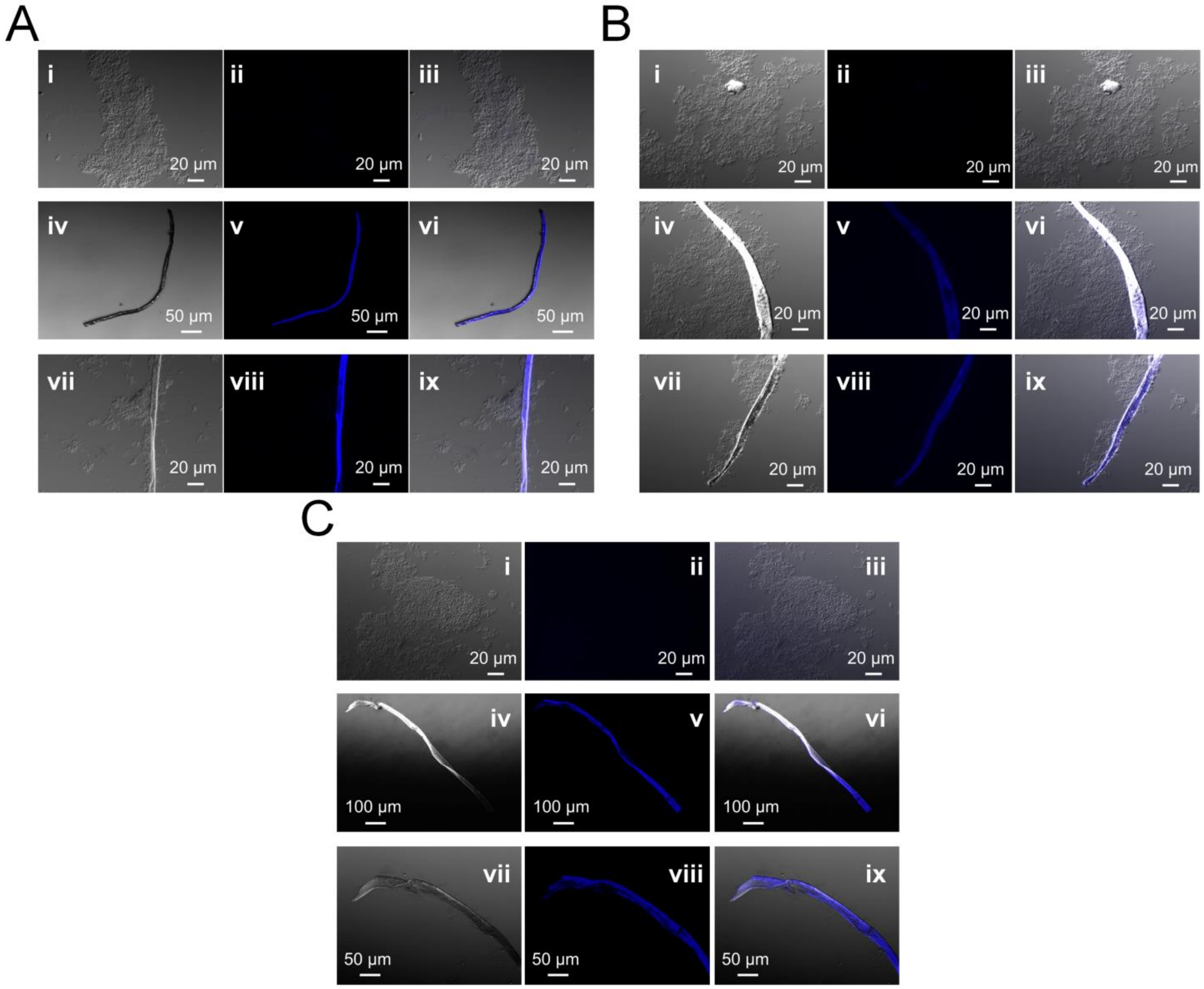

Figure S36. Fluorescence microscopy of Ail aggregates. Representative fluorescence images of WT (A), W0Y (B), and W42F (C); showing differential interference contrast images (i, iv, and vii), fluorescence images (ii, v, and viii), and overlay (iii, vi, and ix). As seen in SEM, we observe both fibrillar and amorphous morphologies of Ail aggregates. Interestingly, only the fibrillar aggregates bind to ThT, and the amorphous aggregates do not bind to ThT dye.

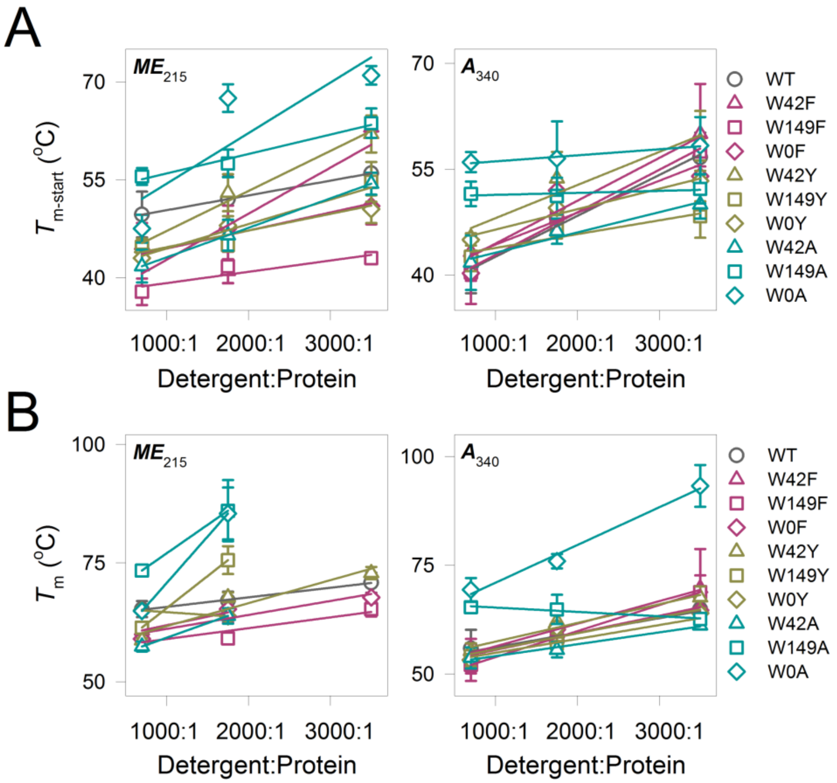

Figure S37. Detergent-protein interaction regulates Ail stability and oligomerization: Inference from thermal denaturation using ME_{215} and A_{340}. Effect of DPR on $T_{\text{m-start}}$ (A) and T_m (B) measured by ME_{215} (left panel) and A_{340} (right panel) for various Ail Trp mutants, at a constant ramp rate of 1 °C/min. The change in thermal parameters is fitted to polynomial equation and fits are shown as solid lines. For some mutants (W42F, W149Y, W0Y, W42A, W149A, and W0A) the $T_{\text{m-ME215}}$ could not be measured at the DPR of 3500:1 (unfolding was not observed at higher DPRs), and only the data for lower DPRs is shown. By and large, all the proteins exhibit a linear increase in thermal parameters upon increasing DPR, suggesting that the stability of Ail proportionally increases with increasing DPR (note the increase in $T_{\text{m-start}}$, and T_m calculated from ME_{215} and A_{340} for almost all the proteins). A simplified color scheme is used here: WT (gray), Phe mutants (pink), Tyr mutants (yellow), Ala mutants (cyan). In all plots, errors are s. d. derived from a minimum of 2–3 independent experiments, except the data for W42F and W149F in a DPR of 3500:1 wherein the result is from a single dataset.

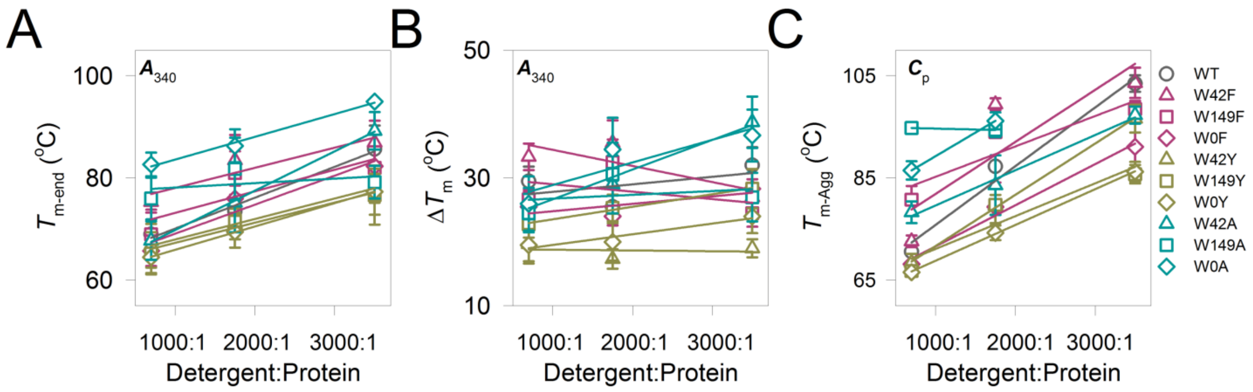

Figure S38. Ail oligomerization and aggregation decreases linearly with DPR: Inference from thermal denaturation using scattering and DSC measurements. Effect of detergent-protein ratio on $T_{\text{m-end}}$ (A), ΔT_{m} (B), and $T_{\text{m-Agg}}$ (C) measured by A_{340} and C_{p} for various Ail Trp mutants, at a constant ramp rate of 1 °C/min. The change in thermal parameters is fitted to polynomial equation and fits are shown as solid lines. We observe a linear correlation of Ail aggregation with DPR, for nearly all the proteins. Here, the proteins show a proportionally lowered aggregation propensity (evident from the increase in $T_{\text{m-end}}$ and $T_{\text{m-Agg}}$), and lowered rate of aggregation (evident from the increase in $T_{\text{m-end}}$ and ΔT_{m}) upon increasing DPR. A simplified color scheme is used here: WT (gray), Phe mutants (pink), Tyr mutants (yellow), Ala mutants (cyan). In all plots, errors are s. d. derived from a minimum of 2 – 3 independent experiments.

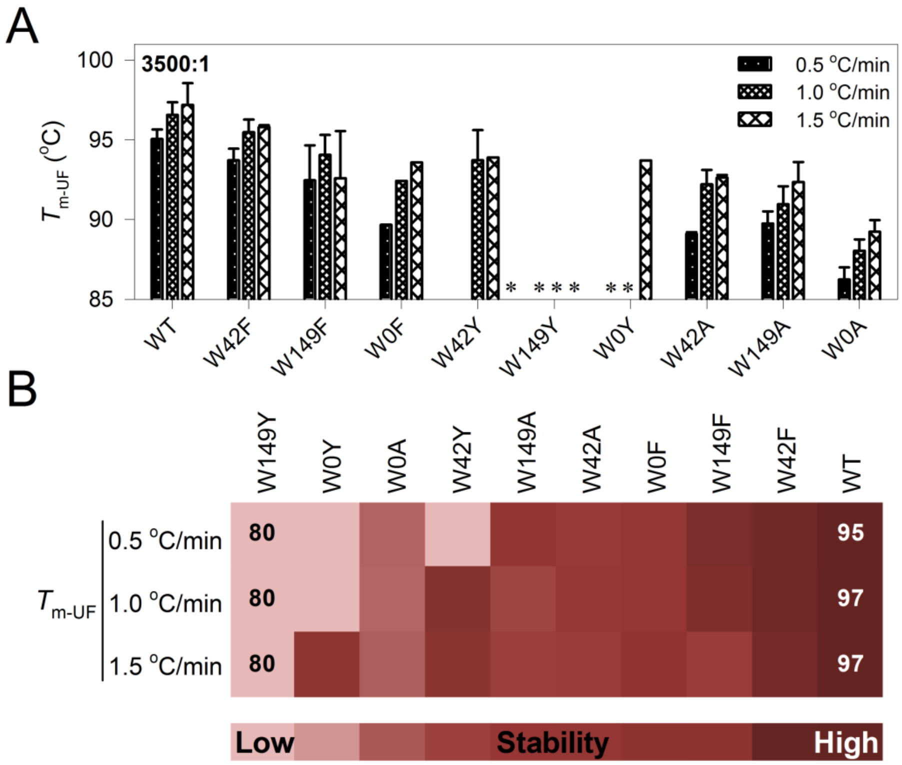

Figure S39. DSC measurements reveal global stability of Ail–WT. (A) Comparison of $T_{m\text{-UF}}$ across the Trp mutants at different temperature ramp rates (0.5 °C/min, 1.0 °C/min, and 1.5 °C/min), at a constant DPR of 3500:1. In all plots, errors are s. d. derived from a minimum of 2–3 independent experiments. Conditions where we were not able to measure $T_{m\text{-UF}}$ because the protein directly aggregates have been denoted as (*). (B) Global comparison of $T_{m\text{-UF}}$ calculated for Ail Trp mutants at various temperature ramp rates, at a constant DPR of 3500:1. Mutants with lowest (light maroon) and highest (dark maroon) overall stability are arranged in increasing order from left to right in the heat map. Numbers within the heat map show the range of each parameter (errors are not included). Ail–WT show highest $T_{m\text{-UF}}$ across all the proteins and the Trp→Tyr mutants show the lowest $T_{m\text{-UF}}$ value.

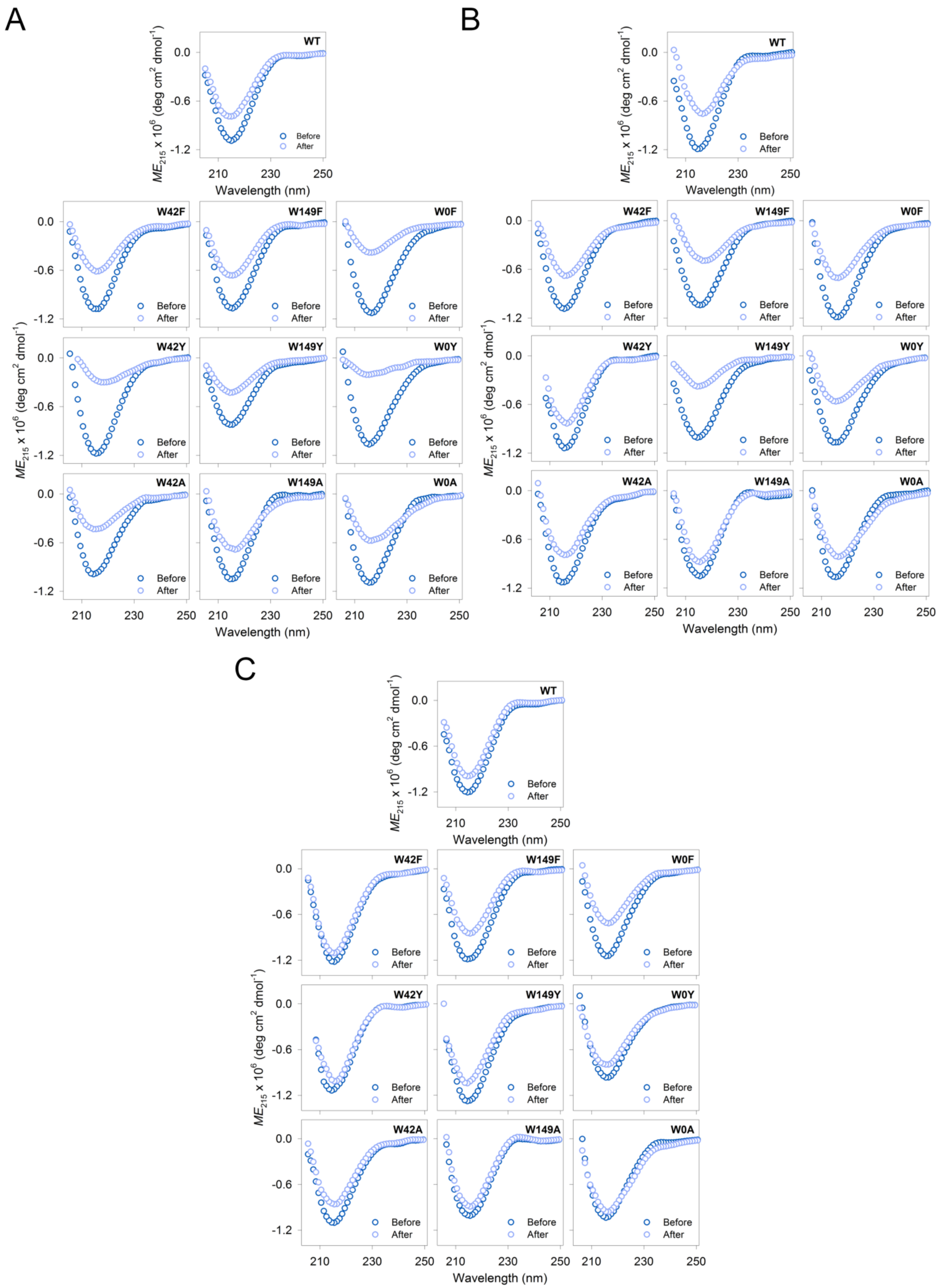

Figure S40. Ail exhibits loss in structure upon thermal denaturation: Inference from thermal denaturation measurements using far-UV CD. Representative far-UV CD profiles of before and after

thermal denaturation samples of Ail Trp mutants at DPRs of 700:1 (A), 1750:1 (B), and 3500:1 (C). The far-UV CD profiles were recorded at 25 °C, before ($ME_{215\text{-B}}$) and after ($ME_{215\text{-A}}$) thermal denaturation. As Ail shows irreversible denaturation and aggregation, we observe a decreased ME_{215} after ($ME_{215\text{-A}}$) thermal denaturation. The ratio in ME_{215} values upon thermal denaturation ($ME_{215\text{-B/A}}$) was used to calculate the extent of aggregation for all the proteins (see Fig. S41). The minimum loss in secondary structure content was seen for all the proteins at the DPR of 3500:1.

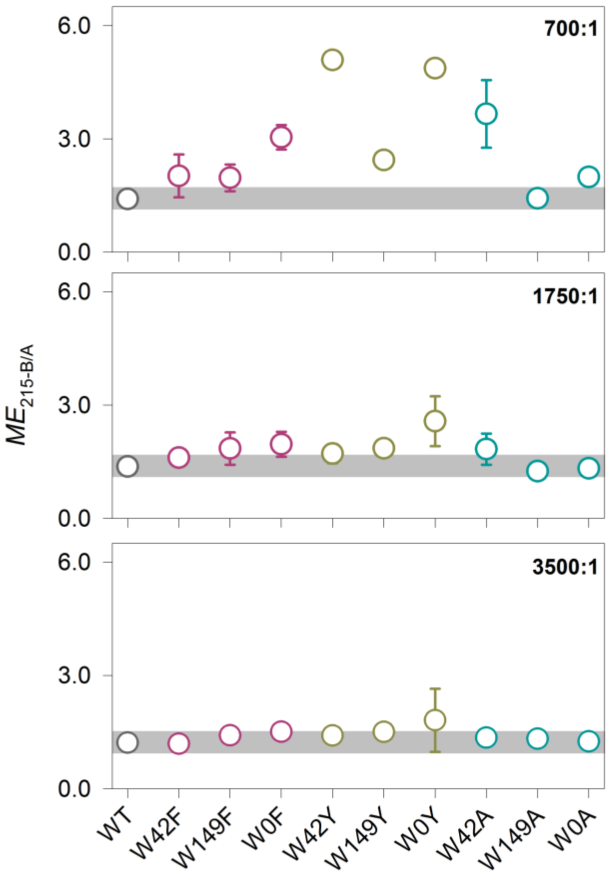

Figure S41. Unfolding and aggregation highest for Ail Tyr mutants. The extent of secondary structure loss ($ME_{215\text{-}B/A}$) and protein aggregation (amount of aggregated protein) measured for various Ail Trp mutants, at various DPRs (700:1, top panel; 1750:1, middle panel; and 3500:1, bottom panel). A simplified color pattern is used here: WT (gray), Phe mutants (pink), Tyr mutants (yellow), Ala mutants (cyan). In all plots, errors are s. d. derived from a minimum of 2–3 independent experiments, except for W42F and W149F in a DPR of 3500:1 wherein the data is from a single experiment. The shaded area in all plots represents the s. d. obtained for wild type Ail protein. The value of $ME_{215\text{-}B/A}$ is highest for the Tyr mutants when the DPR is lowered, suggesting that Trp→Tyr mutants have an intrinsically high tendency to aggregate. Further, the proteins require high DPR for enhanced stability and for unfolding and aggregation to remain minimal.

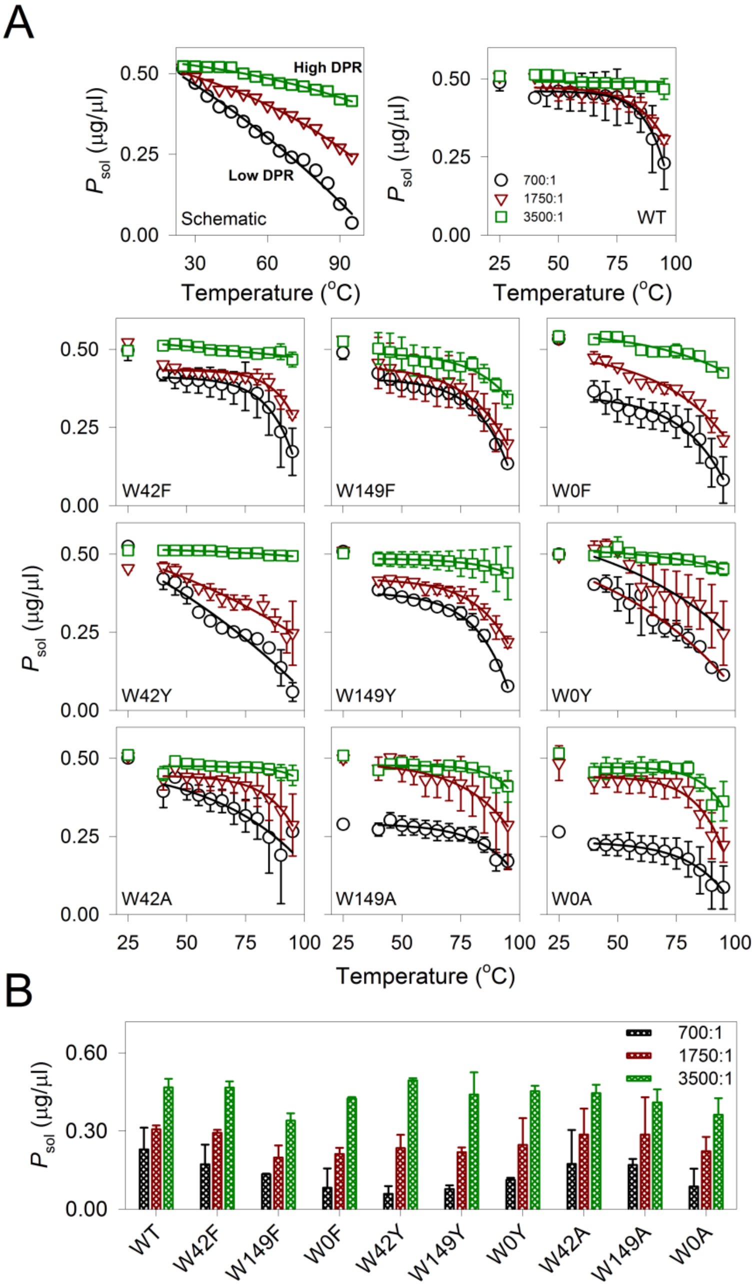

Figure S42. Extent of aggregation is lowest in Ail–WT. (A) Change in P_{sol} at various DPRs (700:1, black; 1750:1, red; and 3500:1, green) with increasing temperature. The initial protein concentration for all the samples was 0.5 µg/µl before the experiment (W149A and W0A proteins in lowest DPR of 700:1 had lower protein concentrations, as these proteins show aggregation while folding in lowest DPR of 700:1; see Fig. S4). Aggregation was induced by heating the folded sample at various temperatures (40 – 95 °C) for 10 min, followed by cooling the sample to 25 °C. The P_{sol} was then calculated after removing the aggregated sample by centrifugation at 18,000 x *g* at 10 °C for 1 h. The change in P_{sol} is fitted to an exponential decay function (fits are shown as solid lines). (B) Comparison of P_{sol} calculated at 95 °C for various Ail Trp mutants at all three DPRs. In all plots, errors are s. d. derived from a minimum of 2 – 3 independent experiments. The extent of protein aggregation decreases proportionally as we increase the DPR.

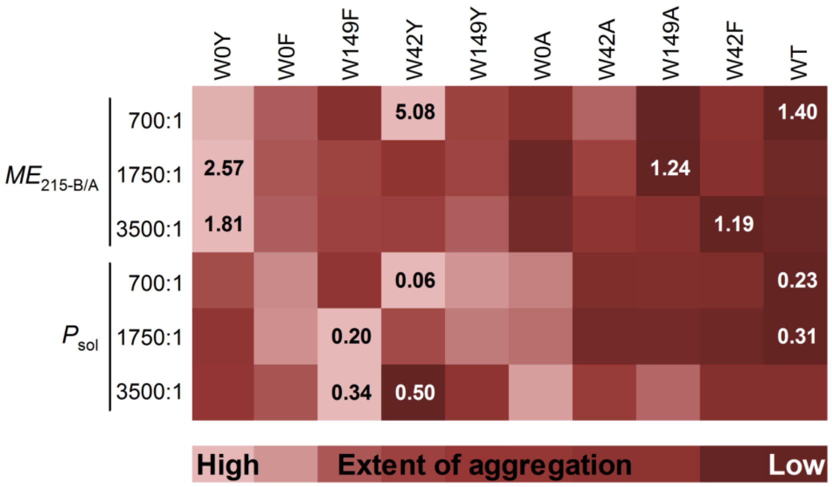

Figure S43. Lowest aggregation of Ail–WT explains evolutionary selection of interface tryptophans. Global comparison of $ME_{215\text{-B/A}}$ and P_{sol} at various DPRs (700:1, top panel; 1750:1, middle panel; and 3500:1, bottom panel). Here, we compare the extent of secondary structure loss and aggregation across the proteins. Ail–WT proteins show the highest stability and least extent of aggregation.

SI REFERENCES

1. Gupta, A., Zadafiya, P., and Mahalakshmi, R. (2014) Differential contribution of tryptophans to the folding and stability of the attachment invasion locus transmembrane beta-barrel from Yersinia pestis. *Sci. Rep.* **4**, 6508

2. Gupta, A., Iyer, B. R., Chaturvedi, D., Maurya, S. R., and Mahalakshmi, R. (2015) Thermodynamic, structural and functional properties of membrane protein inclusion bodies are analogous to purified counterparts: case study from bacteria and humans. *RSC Adv.* **5**, 1227–1234

3. Maurya, S. R., Chaturvedi, D., and Mahalakshmi, R. (2013) Modulating lipid dynamics and membrane fluidity to drive rapid folding of a transmembrane barrel. *Sci. Rep.* **3**, 1989

4. Marshall, K. E., Vadukul, D. M., Dahal, L., Theisen, A., Fowler, M. W., Al-Hilaly, Y., Ford, L., Kemenes, G., Day, I. J., Staras, K., and Serpell, L. C. (2016) A critical role for the self-assembly of Amyloid-beta1-42 in neurodegeneration. *Sci. Rep.* **6**, 30182

5. Gupta, A., and Mahalakshmi, R. (2019) Helix-strand interaction regulates stability and aggregation of the human mitochondrial membrane protein channel VDAC3. *J. Gen. Physiol.*

6. Makwana, K. M., and Mahalakshmi, R. (2014) Asymmetric contribution of aromatic interactions stems from spatial positioning of the interacting aryl pairs in beta-hairpins. *Chem. Commun.* **15**, 2357-2360

7. Plesniak, L. A., Mahalakshmi, R., Rypien, C., Yang, Y., Racic, J., and Marassi, F. M. (2011) Expression, refolding, and initial structural characterization of the Y. pestis Ail outer membrane protein in lipids. *Biochim. Biophys. Acta* **1808**, 482-489

8. Matagne, A., Jamin, M., Chung, E. W., Robinson, C. V., Radford, S. E., and Dobson, C. M. (2000) Thermal unfolding of an intermediate is associated with non-Arrhenius kinetics in the folding of hen lysozyme. *J. Mol. Biol.* **297**, 193-210
